# Neuroprotective Effects of Lycopene in Parkinson’s Disease Mice: Potential Modulation of DAT/SLC6A3-Mediated Dopaminergic Pathway

**DOI:** 10.3390/nu18142234

**Published:** 2026-07-09

**Authors:** Jun Xia, Xin-Rui Fan, Lin-Xia Lu, Ci-Li Jifu, Zhen-Yu Xu, Jing-Tao Wang

**Affiliations:** 1 College of Basic Medical Sciences, Jiamusi University, Jiamusi 154007, China; 15590856654@163.com (J.X.); lulinxia@stu.jmsu.edu.cn (L.-X.L.); jfcl127701@gmail.com (C.-L.J.); xuzhenyu@jmsu.edu.cn (Z.-Y.X.); 2 College of Biology and Agriculture, Jiamusi University, Jiamusi 154007, China; 13936598186@163.com

**Keywords:** neurodegeneration, lycopene, dopamine homeostasis, neuronal protection

## Abstract

**Background/Objectives**: Lycopene (LYC), a natural lipid-soluble antioxidant extracted from dietary plants, possesses excellent neuroprotective and metabolic regulatory activities and has shown potential preventive and therapeutic effects on neurodegenerative diseases. However, its precise molecular mechanism against Parkinson’s disease (PD) remains incompletely clarified, which limits its further application. **Methods**: In this study, a PD mouse model was established, and a series of methods, including molecular docking, untargeted metabolomics, behavioral tests, and histopathological analysis, were used. **Results**: The results showed that LYC intervention not only improved the motor coordination ability of mice and reduced dopaminergic neuron damage but also reversed the metabolic disorders caused by the disease. Importantly, LYC can significantly increase the expression level of DAT/SLC6A3 in the midbrain region, and this is evident at both the gene and protein levels. Computational simulations and surface plasmon resonance results show that LYC can form stable complexes with the DAT/SLC6A3 protein and has a high affinity. **Conclusions**: This study explored the potential anti-PD effects of LYC from multiple perspectives and observed an association between elevated DAT/SLC6A3 levels and improved dopamine (DA) homeostasis after LYC intervention. These preliminary findings may offer new experimental clues for further mechanistic study and translational research on PD prevention and treatment.

## 1. Introduction

Parkinson’s disease (PD), the second most common neurodegenerative disorder, causes a substantial loss of dopaminergic neurons, while the dopamine (DA) content in the striatum is also reduced, resulting in motor disturbances such as rigidity and bradykinesia. In addition to motor symptoms, patients with PD also exhibit non-motor symptoms, including autonomic dysfunction, which significantly impairs their quality of life [1,2,3]. As one of the most prevalent neurodegenerative disorders, Parkinson’s disease currently affects approximately 11.8 million individuals worldwide, with a global age-standardized prevalence of 138.6 per 100,000 population [4], and its disease burden is projected to double by 2050, placing a heavy socioeconomic burden on global public health [5]. PD has a complex pathogenesis, which is associated with a range of factors such as oxidative stress, mitochondrial dysfunction, neuroinflammation, and genetic susceptibility. A central factor is the disruption of DA homeostasis, largely due to solute carrier family 6 member 3 (DAT/SLC6A3) dysfunction, which leads to the vulnerability of dopaminergic neurons [6,7,8]. DAT/SLC6A3 belongs to a family of membrane transporters that mediate synaptic DA reuptake [9,10]. If the activity of DAT/SLC6A3 is reduced, DA signaling will be disturbed, and more DA will accumulate in the cytoplasm, which will lead to the formation of quinone and, consequently, oxidative stress [11]. There is a close link between DAT/SLC6A3 and dopaminergic neurodegeneration, which deserves attention in the treatment of PD.

Existing PD therapies, predominantly 3,4-Dihydroxyphenyl L-Alanine (L-DOPA) and DA agonists, provide symptomatic relief but fail to halt disease progression [12,13]. The use of L-DOPA for a long time will lead to various complications, which are related to the stimulation of DA receptors and the restoration of DA to non-physiological levels [14]. These therapies do not target upstream pathogenic cascades, such as the damage of dopaminergic terminals and DAT/SLC6A3. Therefore, there is a need to develop effective multi-target drugs that maintain the integrity of dopaminergic neurons, particularly by enhancing DAT/SLC6A3 function. Previous studies have suggested that phytochemicals can modulate PD-related pathways [15,16,17]. Lycopene (LYC) is a carotenoid, which is rich in tomatoes and can play an important role in neurodegenerative diseases [18,19].

LYC, a tetraterpene compound, can eliminate free radicals and exhibits potent antioxidant activity, enabling it to neutralize reactive oxygen species and inhibit lipid peroxidation, a key factor in the pathogenesis of PD [20]. The planar structure of LYC enables it to readily cross the blood–brain barrier, a prerequisite for its neuroprotective effects [21]. In addition to eliminating free radicals, LYC can also inhibit the NF-κB pathway and reduce the expression of pro-inflammatory factors (such as IL-1β and TNF-α) [22]. In animal models of Alzheimer’s disease, LYC can not only reduce amyloid-β toxicity but also improve cognitive deficits, and it can play the role of protecting neurons [23]. Nevertheless, few studies have explored the direct interaction between LYC and DAT/SLC6A3. Given that the dysfunction of DAT/SLC6A3 occurs before significant neuronal loss in PD and may serve as a preventive target, this research gap is particularly important.

DAT/SLC6A3 polymorphisms (e.g., SLC6A3 9/10 repeat) are linked to PD susceptibility and progression, likely through altered transporter trafficking and surface expression [24]. Postmortem studies reveal reduced DAT/SLC6A3 protein levels in the striatum of PD patients, correlating with DAergic denervation severity [25]. Pharmacological DAT/SLC6A3 inhibition exacerbates DA toxicity in vitro, while its upregulation mitigates ROS generation and apoptosis in DAergic cells [26]. The expression of DAT/SLC6A3 is regulated by transcription factors such as Nurr1 and Pitx3, which are closely associated with the pathogenesis of PD [27,28]. Studies have shown that certain natural compounds, like resveratrol, possess the potential to upregulate DAT/SLC6A3 expression, thereby exerting protective effects in PD models [29]. LYC, a potent antioxidant, has also been identified as an effective Nrf2 agonist [30], suggesting its potential regulatory role in DAT/SLC6A3 expression. However, whether LYC can directly modulate DAT/SLC6A3 and subsequently influence dopaminergic system function remains to be confirmed by research.

Despite the documented neuroprotective effects of LYC, its impact on DAT/SLC6A3-mediated DA homeostasis remains unknown. We hypothesize that the protective effect of LYC on PD is derived from its maintenance of DAT/SLC6A3 function by antagonizing oxidative stress-induced transporter inactivation and blocking the neuroinflammatory cascade reaction that damages transporter transport function, thereby exerting therapeutic effects. Supporting this hypothesis, LYC can upregulate glutathione synthesis and mitochondrial complex I activity, which are crucial for DAT/SLC6A3 function and rescue synaptic protein loss in aging models [31,32]. In addition, LYC can also trigger the activation of the PI3K/Akt pathway [33], which is a key regulatory factor for DAT/SLC6A3 membrane transport, indicating that LYC has multifaceted therapeutic potential in improving PD pathology. Verifying the effect of LYC on DAT/SLC6A3 can establish it as a disease regulator while addressing the issues of DA depletion and upstream neurodegeneration in PD.

Existing studies have confirmed that LYC exerts favorable neuroprotective effects and activates neuronal survival pathways. However, the regulatory effect of LYC on DAT/SLC6A3 remains poorly clarified. Notably, dysfunction of DAT/SLC6A3 occurs prior to overt neurodegenerative lesions in PD, representing a critical intervention window for the early prevention and treatment of PD. Considering the limitations of current clinical therapies that fail to halt PD progression, it is urgent to explore novel natural therapeutic targets and intervention strategies. Against this background, the present study aims to further clarify the therapeutic value of LYC in PD treatment via modulating DAT/SLC6A3 and maintaining neuronal survival. Specifically, this study focuses on two core objectives: to evaluate the regulatory effect of LYC on the expression and activity of DAT/SLC6A3 in PD models and to clarify the correlation between motor function improvement and DAT/SLC6A3 functional restoration after LYC intervention.

## 2. Materials and Methods

### 2.1. Animals

Male C57BL/6J mice (7–8 weeks old, 20–22 g) were purchased from Changchun Yisi Experimental Animal Technology Co., Ltd. (Changchun, China) and housed under specific pathogen-free (SPF) conditions. The mice were housed in the experimental animal center with free access to food and water, under a 12 h light/dark cycle, with the ambient temperature maintained at 23–25 °C. After one week of acclimatization, the mice were randomly divided into five groups (25 mice per group, 125 mice in total): a blank control group (Con), a vehicle control group (Vcon), a lycopene group (LYC), a PD model group (PD), and a lycopene treatment group (LYC-PD). The specific treatments were as follows: the Con group received no special treatment; the Vcon group was administered corn oil (5 mg/kg) by gavage; the LYC group was administered LYC (5 mg/kg) by gavage; the PD model group was intraperitoneally injected with 1-Methyl-4-Phenyl-1,2,3,6-Tetrahydropyridine (MPTP) (30 mg/kg) to establish a subacute PD model; and the LYC-PD group received LYC (5 mg/kg) by gavage along with intraperitoneal injections of MPTP (30 mg/kg). The above control groups were set up to exclude experimental interference caused by feeding conditions and solvent administration. From the first day of the experiment, mice in the LYC and LYC-PD groups were intragastrically administered lycopene at a fixed time daily, and the other groups received an equal volume of corn oil vehicle in parallel. Starting on day 7 of pretreatment, the PD and LYC-PD groups received consecutive intraperitoneal injections of 30 mg/kg MPTP for five days to establish a subacute Parkinson’s disease model, whereas the remaining groups were injected with an equal volume of sterile saline. A 4 h interval was maintained between modeling injection and gavage to avoid drug interaction. Following the completion of MPTP administration, gavage treatment in each group was continued until the end of the 21-day experimental cycle. The next day, anesthesia infusion was performed to collect serum and brain tissue samples for subsequent experimental testing. All animal experiments were performed in accordance with the ARRIVE Guidelines 2.0, and the completed ARRIVE Guidelines 2.0 checklist file has been submitted as Supplementary Material (Table S1).

### 2.2. Inclusion and Exclusion Criteria and Blinding Design

All experimental criteria were predefined before the formal experiment. Only healthy mice with normal mental status, feeding and drinking behavior, and body weight were included. Mice that died during feeding or modeling, developed severe complications, or exhibited abnormal physiological conditions were excluded. In addition, invalid samples caused by detection failure and abnormal data were eliminated. A stratified blinding design was adopted in this study: random grouping of mice was performed by a dedicated researcher who did not participate in subsequent experimental operations. Blinding was not feasible during modeling and administration due to the distinct differences in intervention procedures among groups. However, all behavioral and molecular indicator detections were conducted under full blinding to avoid observational bias and ensure the objectivity and reliability of experimental data.

According to the experimental plan, 30 mice were initially assigned to each group. During model establishment and feeding, only one mouse in the PD group died, and no mice died in the other groups. No animals were excluded due to severe complications or abnormal physical conditions. The final sample size for data analysis was as follows: Con group (*n* = 30), Vcon group (*n* = 30), LYC group (*n* = 30), PD group (*n* = 29), and LYC-PD group (*n* = 30). All final sample numbers are listed in the corresponding figure legends.

### 2.3. Open Field Test (OFT)

The OFT is commonly used to evaluate spontaneous activity, exploratory behavior, and anxiety-like behavior in mice [34]. The apparatus was a 40 cm × 40 cm × 40 cm open chamber. The testing environment was kept quiet with soft lighting to minimize external disturbances. Prior to testing, mice were acclimated to the laboratory for 15 min to reduce stress. Each mouse was gently placed in the same corner of the chamber, and a 5 min video recording was started immediately. Automated analysis measured the total distance traveled, locomotor speed, distance traveled in the central zone, number of entries into the central zone, and time spent in the central zone. Upon completion of the trial, the mouse was promptly returned to its home cage. The chamber was thoroughly cleaned with 75% ethanol to eliminate odor cues before testing the next subject.

### 2.4. Pole Test (PT)

The PT is commonly used to evaluate motor coordination and balance in mice [35]. The apparatus consisted of a 50 cm long, 1 cm diameter wooden pole with a rough surface, vertically fixed with a 2.5 cm diameter anti-perching ball at its top. Prior to formal testing, mice underwent a 3-day adaptation period. At the start of the test, the mouse was placed head-up on the top sphere, and timing began. The time required to complete a 180° turn and the total descent time from the top to the base where it contacted the ground were recorded. Each mouse underwent three trials, with a 30 s rest interval between each, and the average time was analyzed. If the mouse slipped off or remained stationary for an extended period during the test, the trial needed to be repeated. After each mouse completed testing, it was promptly returned to its housing cage. The pole was cleaned with 75% ethanol to eliminate odor interference.

### 2.5. Tail Suspension Test (TST)

The tail suspension test is commonly used to assess depressive-like behavior in mice [36]. Prior to the experiment, mice were acclimated to the laboratory environment for 30 min to mitigate stress induced by the new surroundings. At the start of the test, the tail was secured with medical tape approximately 1 cm from the tip; then, the mouse was suspended in a specialized tail suspension chamber with its head positioned 20–30 cm above the chamber floor. The cumulative immobility time was then recorded. After the experiment concluded, the tape was gently removed from the mouse’s tail, and the mouse was returned to its housing cage. The chamber was cleaned with 75% ethanol to eliminate odor interference.

### 2.6. Rotarod Test (RT)

The rotarod test is used to evaluate a mouse’s motor coordination, balance, and endurance [37]. Prior to testing, mice were acclimated to the laboratory environment for 30 min, followed by a 3-day adaptation training period. During the formal testing phase, the mouse was placed on the rotarod facing away from the direction of rotation. The uniform acceleration program was initiated, and the latency from start to fall was recorded. Each mouse underwent three independent tests, with the final analysis based on the average of the three latency values.

### 2.7. Hematoxylin–Eosin (H&E) Staining

Hematoxylin–Eosin staining was performed with *n* = 3 biological replicates per group, and representative images were selected for display. Following anesthesia with sodium pentobarbital, mice were transcardially perfused through the left ventricle, first with normal saline, followed by 4% paraformaldehyde for fixation. Brains were removed, and serial coronal sections between Bregma −2.96 mm and −3.06 mm were obtained. Selected sections were baked, deparaffinized, and rehydrated through a graded ethanol series. The sections were then stained with hematoxylin for 15 min, differentiated in 1% acid-alcohol for 4 s, and counterstained with eosin. Finally, sections were dehydrated, cleared, and coverslipped. The cellular morphology in the substantia nigra and striatum was observed and compared across groups under a light microscope (Olympus Corporation, Tokyo, Japan).

### 2.8. Nissl Staining

Nissl staining was performed with *n* = 3 biological replicates per group, and typical microscopic photographs were presented in figures. Serial brain sections were immersed in toluidine blue staining solution for 10 min, followed by rinsing with distilled water. The sections were then differentiated with Nissl differentiation solution under microscopic monitoring until the background became nearly colorless and the Nissl bodies appeared deep blue, at which point differentiation was immediately terminated. The sections were rinsed under running water for 5 min to thoroughly remove the differentiation solution. Subsequently, the sections were dehydrated through a graded ethanol series (70%, 85%, and 95% ethanol, 1 min each) and then in two changes of absolute ethanol (3 min each). After dehydration, the sections were cleared in two changes of xylene (5 min each). Finally, the sections were mounted with neutral gum, dried at room temperature in the dark, and observed and photographed under a light microscope.

### 2.9. Detection of Untargeted Metabolomics

Each experimental group contained five biological replicates for untargeted metabolomics analysis. Frozen samples were thawed on ice, and 50 µL of each sample was mixed with 300 µL of ice-cold extraction solution (acetonitrile:methanol = 1:4, *v*/*v*) containing internal standards. After vortexing and centrifugation, the supernatant was collected, held at −20 °C for 30 min, and centrifuged again. A 180 µL aliquot of the resulting supernatant was subjected to LC-MS analysis. Chromatographic separation was performed on a Waters ACQUITY Premier HSS T3 column (1.8 µm, 2.1 × 100 mm) using a gradient of 0.1% formic acid in water (solvent A) and 0.1% formic acid in acetonitrile (solvent B) at a flow rate of 0.4 mL/min. Mass spectrometry was conducted in both positive- and negative-ion modes with electrospray ionization, scanning *m*/*z* 75–1000 at a resolution of 35,000. Data were processed by principal component analysis (PCA) and orthogonal partial least squares–discriminant analysis (OPLS-DA). Differential metabolites were selected based on variable importance in projection (VIP) >1 and *p* < 0.05. Metabolites were annotated using the Kyoto Encyclopedia of Genes and Genomes (KEGG) compound database, and pathway enrichment analysis was performed.

### 2.10. Detection of Neurotransmitters

Four biological replicates were included in each experimental group for targeted neurotransmitter metabolomics detection. Neurotransmitter contents were detected by MetWare (http://www.metware.cn/) based on the AB Sciex QTRAP 6500 LC-MS/MS platform. Targeted neurotransmitter metabolomics detection was performed with *n* = 4 biological replicates per group. Statistical analysis was performed in R. PCA was conducted on unit variance-scaled data using the prcomp function. Hierarchical cluster analysis and Pearson correlation coefficients were calculated and visualized as heatmaps using the pheatmap package (v1.0.13, R software). Differential metabolites were selected based on the absolute value of log2 (fold change).

### 2.11. RNA-Seq Preprocessing and Transcriptomic Analysis

Six biological replicates were arranged for each group in transcriptomic sequencing. The cDNA libraries were sequenced on the Illumina sequencing platform by Metware Biotechnology Co., Ltd. (Wuhan, China). RNA-seq transcriptomic profiling was conducted with *n* = 3 biological replicates per group. Total RNA was extracted from plant and animal samples using CTAB-PBIOZOL and Trizol methods, respectively, with quality assessed via Qubit 4 Fluorometer (Thermo Fisher Scientific, Waltham, MA, USA) and Qsep400 Bio-Fragment Analyzer (BiOptic Inc., New Taipei City, Taiwan, China). mRNA libraries were constructed through poly(A) enrichment, fragmentation, and strand-specific cDNA synthesis, followed by Illumina sequencing (150-bp paired-end). After quality control with fastp, clean reads were aligned to a reference genome using HISAT2 (v2.2.1, https://daehwankimlab.github.io/hisat2/ (accessed on 26 May 2026)). Gene expression was quantified as Fragments per kilobase of transcript per million mapped reads (FPKM) with featureCounts, and differential expression analysis was performed using DESeq2. Enrichment analysis for KEGG and Gene Ontology (GO) terms was conducted via a hypergeometric test.

### 2.12. Western Blot Analysis

Tissue samples were lysed using RIPA buffer (Beyotime Biotechnology, Shanghai, China) and centrifuged, and the protein concentration was determined using a BCA protein quantification kit (Beyotime Biotechnology, Shanghai, China). Following heat denaturation in loading buffer, the proteins were separated by SDS–polyacrylamide gel electrophoresis and subsequently transferred onto a polyvinylidene difluoride membrane. The membrane was blocked with 5% non-fat milk and then incubated with primary antibodies (all primary antibodies were diluted at a ratio of 1:1000 according to the manufacturer’s instructions, Santa Cruz Biotechnology, Inc., Dallas, TX, USA at 4 °C overnight, followed by incubation with an HRP-conjugated secondary antibody (Boster Biological Technology, Shanghai, China) at room temperature for 1 h. Protein bands were finally visualized using an enhanced chemiluminescence detection system. This study adopted the Thermo PageRuler protein marker (Cat. No. 26616) for molecular weight calibration. All Western blot images were acquired by a chemiluminescence imaging system under unified parameters, with a fixed exposure time of 60 s and a gain value of 4. Each gel was captured via a single complete exposure to guarantee consistent signal acquisition conditions for all samples. All images were obtained in linear detection mode, with automatic contrast adjustment, saturation correction, and other auto-optimization functions disabled. No protein bands exhibited overexposure, and all grayscale signals were within the linear detection range of the instrument, enabling precise grayscale quantitative analysis. The band intensity of target proteins was normalized to the internal reference protein in the same lane to eliminate loading and operational errors. All Western blot assays were performed with three independent biological replicates to validate the accuracy and reproducibility of the experimental results.

### 2.13. Molecular Docking and Molecular Dynamics Simulation

The receptor protein DAT/SLC6A3 (PDB ID: 9EO4) was retrieved from the Protein Data Bank. The protein’s 3D structure was prepared for docking using PyMOL 2.3.0. For the molecular docking simulation, the three-dimensional structure of lycopene was obtained from the PubChem database (CID: 446925). Considering that lycopene possesses numerous geometric E/Z isomers, the all-trans (all-E) lycopene conformation was adopted in the present study. The all-trans form exhibits the highest thermodynamic stability and dominates in fresh dietary plants, accounting for more than 94% of total lycopene in ripe tomatoes [38]. Moreover, this configuration has been widely recognized and uniformly applied in most computational pharmacological investigations of lycopene [39], ensuring the rationality, comparability, and reproducibility of the present docking results. Its structure was subsequently optimized to its lowest energy conformation using the MMFF94 force field implemented in OpenBabel 3.1.1 software. Molecular dynamics simulations of the LYC-DAT/SLC6A3 complex were performed using Gromacs 2024.4. Protein preparation, partial charge assignment, and flexible bond definition were performed using AutoDock Tools 1.5.6. Polar hydrogens were added to the protein and ligand structures, and all rotatable bonds of the ligand were defined before being saved as PDBQT files. The docking grid was centered at X = 133.2, Y = 129.5, Z = 127.5 with a grid size of 74 × 61 × 61 Å, which fully covered the canonical substrate-binding pocket of DAT/SLC6A3 according to previously reported structural characteristics. Semi-flexible docking was applied, and the Lamarckian genetic algorithm was used with an exhaustiveness value of 15. All docking simulations were performed using AutoDock Vina 1.2.0 to obtain the optimal binding conformation and corresponding binding free energy.

### 2.14. Surface Plasmon Resonance (SPR) Assay

SPR binding assays were performed with *n* = 3 independent chip biological replicates. SPR assays were performed at 25 °C using a Biacore T200 system with CM5 chips in HBS-EP+ buffer (pH 7.4, 0.05% Tween-20). Considering the strong lipophilicity of lycopene, all working solutions were freshly prepared via DMSO gradient dilution with identical final DMSO concentrations to eliminate solvent interference and avoid molecular aggregation. DAT/SLC6A3 and BSA were amine-coupled to the detection and reference channels, respectively, and the DAT/SLC6A3 immobilization level was supplemented in the revised manuscript. Serially diluted lycopene samples were injected at 30 μL/min for binding and dissociation measurements, followed by chip regeneration with glycine–HCl solution. All sensorgrams were processed with blank subtraction and double reference correction to remove background and non-specific signals. Data were fitted to a 1:1 Langmuir binding model, and the fitting residual, kon, koff, and KD values were calculated. Multiple independent biological replicates were conducted, and the corresponding statistical results are provided. BSA reference and blank buffer controls were included throughout the experiment to exclude non-specific binding, ensuring specific and reliable binding signals.

### 2.15. Statistical Analysis

All statistical analyses were performed using SPSS (v19.0, IBM Corporation, Armonk, NY, USA) and GraphPad Prism (v10, GraphPad Software, San Diego, CA, USA). Prior to statistical comparison, the normality of data distribution and homogeneity of variance were systematically tested. For normally distributed data with homogeneous variance, one-way analysis of variance (ANOVA) was used for intergroup comparisons, followed by Tukey’s post-hoc multiple comparison test for pairwise comparisons. Longitudinal repeated-measured data, including body weight, food and water intake, and behavioral indicators detected at multiple time points, were analyzed using repeated-measures ANOVA to eliminate the interference of time-dependent confounding factors. For omics data analysis, the false discovery rate (FDR) method was applied for multiple-testing correction, and the adjusted *p*-values were calculated and reported to control false positives and ensure the reliability of omics results. All quantitative data were presented as mean ± standard deviation. A value of *p* < 0.05 was considered statistically significant.

## 3. Results

### 3.1. Effect of LYC on Basic Physiological Parameters in PD Mice

**Figure 1:**
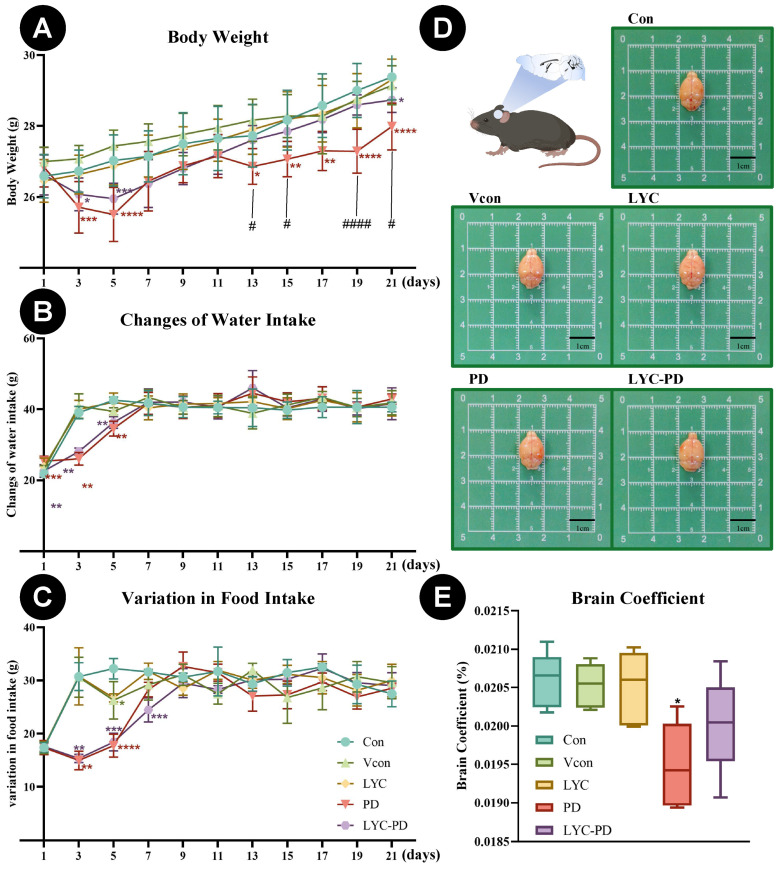
Effect of LYC on basic physiological parameters in PD mice. (**A**) Body weight change curves of mice in each group during the 21-day experimental period. (**B**) Daily water intake change curves of mice in each group during the 21-day experimental period. (**C**) Daily food intake change curves of mice in each group during the 21-day experimental period. (**D**) Representative photographic images of the whole brains isolated from mice in each group, showing the effects of different treatments on the macroscopic morphology of the brain. Scale bar = 1 cm. (**E**) Statistical analysis of the brain coefficient. *n* = 10 mice per group. Asterisks (*) denote comparisons within the same group versus Day 1: * *p* < 0.05, ** *p* < 0.01, *** *p* < 0.001, **** *p* < 0.0001. Hash symbols (#) represent comparisons at the same time point versus the blank group: # *p* < 0.05, #### *p* < 0.0001.

Final sample sizes for different detections differed: ten mice per group were randomly selected for dynamic monitoring of body weight, water intake, and food intake; all surviving animals were subjected to brain anatomical observation and brain coefficient calculation. The number of surviving animals was as follows: Con (*n* = 30), Vcon (*n* = 30), LYC (*n* = 30), PD (*n* = 29), and LYC-PD (*n* = 30). As shown in Figure 1A, body weight analysis showed that compared with the PD group, the weight of the LYC-PD group increased significantly on the 21st day, but it was still lower than that of the Con group (*p* < 0.05). The results indicate that LYC intervention can partially improve the weight loss of the PD model. Figure 1B,C present the changes in water intake and food intake. During intraperitoneal injection of MPTP, the amount of water intake and food intake in the PD and LYC-PD groups was significantly decreased. However, with the passage of time, the two parameters showed a gradual recovery trend, but there was no significant difference. Figure 1D shows that there are no macroscopic morphological differences in brain anatomical structures between treatment groups compared with the Con group. Figure 1E presents the results of the brain coefficient. The results show that the PD group is significantly decreased compared with the Con group, but the LYC-PD group is not significantly different from that of the Con group. Notably, the coefficient of the LYC-PD group is significantly increased compared with that of the PD group (*p* < 0.05). The above results show that LYC can effectively alleviate the weight loss and organ coefficient decline trend of PD mice, and it indicates a protective effect on the physiological parameters of PD mice.

### 3.2. Effect of LYC on Behavioral Indicators in PD Mice

**Figure 2:**
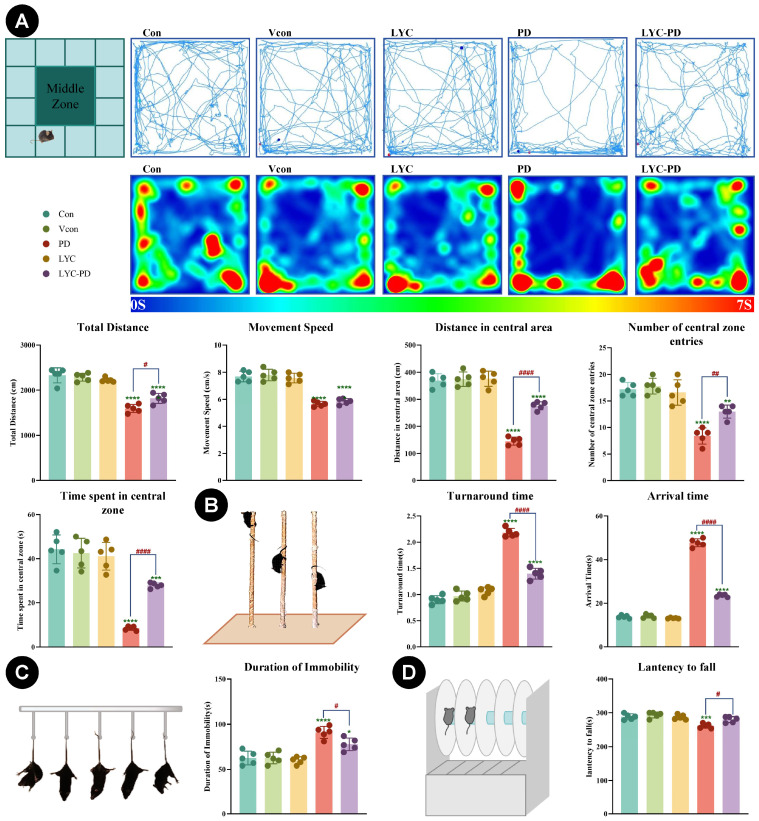
Effect of LYC on behavioral indicators in PD mice. (**A**) OFT: The OFT panel adopts a top-to-bottom layout. The upper part displays the representative motion trajectories of mice and the activity intensity heatmap, in which the color gradient from blue to red indicates the gradual increase in mouse activity frequency and exploration intensity. The lower part contains quantitative statistical bar charts arranged from left to right, showing the total movement distance, average speed, distance traveled in the central area, number of central zone entries, and time spent in the central area. (**B**) PT: Column charts for the statistical analysis of mouse turning time and downward arrival time. (**C**) TST: Column chart showing the statistical results of mouse immobility duration. (**D**) RT: Column chart presenting the statistical results of mouse fall latency. *n* = 5 mice per group for all behavioral indicators. Different colored bars represent different experimental groups: blue = Con group, green = Vcon group, yellow = LYC group, red = PD group, purple = LYC-PD group. Asterisks denote comparisons with the Con group: * *p* < 0.05, ** *p* < 0.01, *** *p* < 0.001, **** *p* < 0.0001. Hash symbols (#) represent comparisons versus the PD group: # *p* < 0.05, ## *p* < 0.01, #### *p* < 0.0001.

In order to evaluate the effect of LYC on the behavior of PD mice, we conducted OFT, PT, TST, and RT to see if there were any changes in the behavior of PD model mice. For behavioral tests (OFT, PT, TST, and RT), five mice were randomly selected from each group for detection and statistical analysis. As shown in Figure 2A, we evaluated the motor activity, exploratory behavior, and anxiety level of mice by OFT. Compared with the PD group, the total distance, distance to the central area, number of entries to the central area, and time spent in the central area in the LYC-PD group were significantly increased. These results indicate that although LYC cannot completely restore the normal behavior pattern, it can partially reduce the behavioral injury of PD mice. Figure 2B presents results from the PT evaluating turnaround time and arrival time. Compared with the Con group, the turnaround time and arrival time of the PD group were significantly increased. whereas the LYC-PD group showed a significant decrease in turnaround time and arrival time compared with the PD group. The LYC-PD group exhibited a significant decrease in immobility time and a marked increase in active movement time compared to the PD group (*p* < 0.0001). While showing potential in counteracting PD-related deficits, the LYC-PD group did not reach the baseline levels observed in the Con group. Figure 2C shows the results of the TST. Compared with the Con group, the duration of immobility in the PD group was significantly increased (*p* < 0.0001). Compared with the PD group, the immobility time of the LYC-PD group was significantly decreased (*p* < 0.05). However, this reduction did not restore levels to those observed in the Con group. Figure 2D shows the results of the RT. Compared with the Con group, the fall latency of the PD group was significantly shorter, indicating impaired motor function. The fall latency of the LYC-PD group was longer than that of the PD group, suggesting that LYC improved motor performance in the PD mice. The behavioral results demonstrate that LYC played a positive role in alleviating motor deficits in PD mice.

### 3.3. Impact of LYC on Midbrain Histopathology in PD Mice

**Figure 3:**
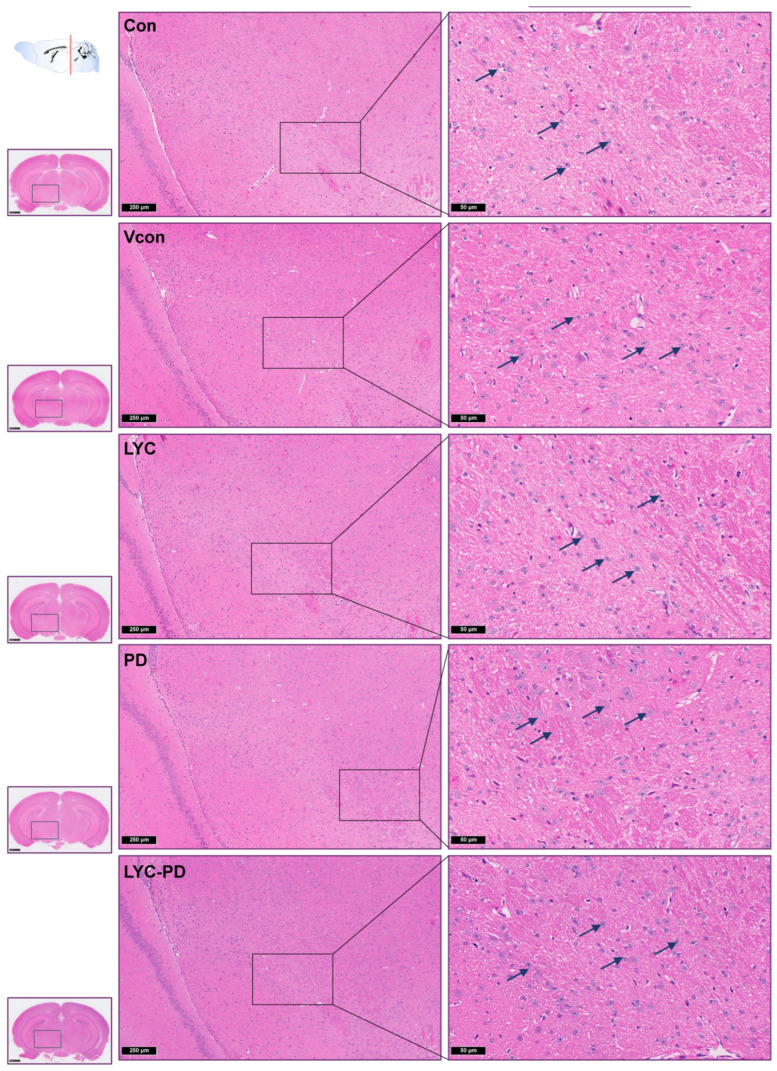
H&E staining of midbrain tissue in mice. Arrows indicate typical pathological changes. *n* = 3 biological replicates per group for histological staining. Arrows indicate damaged neurons.

**Figure 4:**
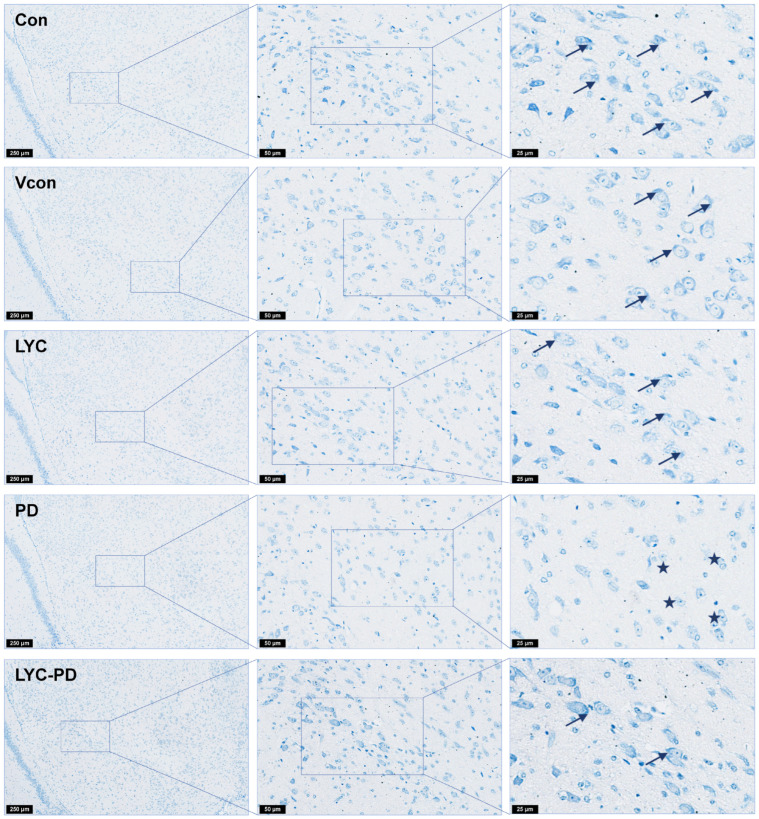
Nissl staining of midbrain tissue in mice. *n* = 3 biological replicates per group for histological staining. Arrows indicate normal Nissl bodies, and pentagrams represent damaged Nissl bodies.

To further investigate the effect of LYC on midbrain neuropathology in PD mice, we performed H&E and Nissl staining to evaluate histopathological changes in the midbrain. H&E/Nissl staining analysis of midbrain tissues was conducted with *n* = 3 biological replicates per group. As shown in Figure 3, H&E staining revealed that the midbrain structure remained unchanged in the Con, Vcon, and LYC groups. In contrast, the PD group exhibited obvious neuropathological changes, including abnormal neuronal morphology (pyknosis and vacuolar degeneration) and decreased cell density. Notably, these pathological lesions were significantly attenuated in the LYC-PD group. As shown in Figure 4, Nissl staining results showed that neurons in the Con group were plump in shape and rich in Nissl bodies, while Nissl bodies in the PD group were reduced in density, dissolved in large amounts, and stained lightly. The density of Nissl bodies in the LYC-PD group was significantly restored. The results showed that LYC had a potential neuroprotective effect on midbrain pathological injury.

### 3.4. The Regulatory Effects of LYC on Untargeted Serum Metabolomics in PD Mice

**Figure 5:**
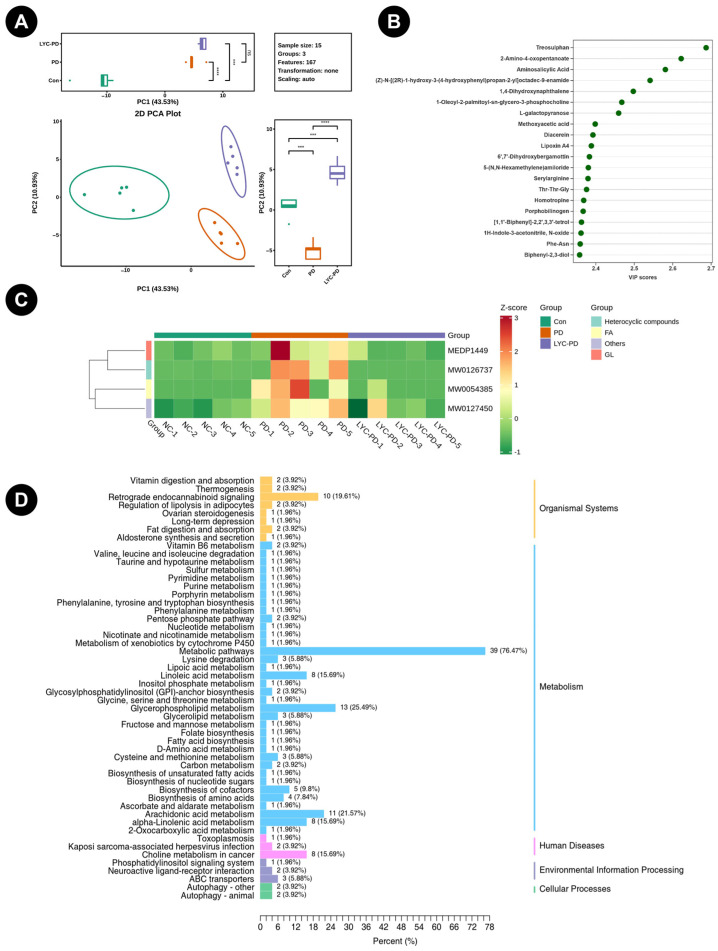
Untargeted metabolomic analysis of serum samples from mice across different experimental groups. (**A**) PCA score plot. (**B**) VIP score plot. (**C**) Differential abundance metabolite heatmap. The index of MW0126737 is tetrahydropapaveroline. The index of MW0054385 is Lipoxin A4. The index of MW0127450 is Diisopropyl sulfide. The index of MEDP1449 is MG (0:0/22:6/0:0). (**D**) KEGG pathway enrichment analysis. Statistical significance was defined as FDR-adjusted *p* < 0.05. Metabolomic profiling was performed with *n* = 5 biological replicates per group. Compared with the Con group, *** *p* < 0.001, **** *p* < 0.0001; ns, no significant difference.

Metabolomic analysis was performed using serum samples from five biological replicates per group. Given the close association between disease progression and multiple biological processes, we hypothesized that LYC might mediate the pathological progression of PD by modulating metabolite levels. To validate this hypothesis, untargeted metabolomic profiling was performed on serum samples from mice across experimental groups. As illustrated in Figure 5A, PCA revealed tight intra-group clustering, indicating good experimental reproducibility, while inter-group comparisons exhibited a clear separation trend, suggesting significant metabolic differences. The NC and PD groups were completely separated along the PC1 axis, demonstrating that disease induction led to substantial metabolic disturbances. After intervention, the LYC-PD group was located between the PD and NC groups in the plot and showed clear separation from the PD group along the PC2 axis. These results indicate that the intervention could partially reverse the disease-induced metabolic abnormalities, shifting the overall metabolic profile toward the normal state. Untargeted metabolomics identified 167 differentially abundant metabolites, predominantly categorized into 19 classes, including benzene and substituted derivatives, organic acids and derivatives, heterocyclic compounds, glycerophospholipids (GP), amino acids and their metabolites, and fatty acids (FA). The top 20 metabolites with VIP > 1 are displayed in Figure 5B. We further set the thresholds of *p* < 0.05 and VIP > 2, and we performed one-way analysis of variance (ANOVA). As a result, four metabolites with highly significant changes were screened out (Figure 5C). Detailed statistical parameters of significantly differential metabolites are provided in Table S2. As shown in Figure 5D, KEGG pathway enrichment analysis revealed that the differentially expressed metabolites were predominantly enriched in pathways associated with “Metabolism,” accounting for 76.5% (39 pathways) of all enriched pathways. Among these, glycerophospholipid metabolism (25.5%, 13 pathways) and arachidonic acid metabolism (21.6%, 11 pathways) exhibited the highest degree of enrichment. Additionally, the altered metabolites were also involved in other metabolic pathways, including linoleic acid metabolism, alpha-linolenic acid metabolism (both 15.69%, eight pathways), choline metabolism in cancer (13.73%, seven pathways), and cysteine and methionine metabolism (7.84%, four pathways). Subsequently, we mapped the metabolites identified in Figure 3 to the KEGG pathway map in Figure 5 and found that these four metabolites were enriched in the highly proportioned pathways of arachidonic acid metabolism and glycerophospholipid metabolism. These results suggest that LYC may exert its effects through broader biological processes, including the modulation of neuroprotective signaling.

### 3.5. Effects of LYC on Targeted Neurotransmitter Metabolomics in PD Mice

**Figure 6:**
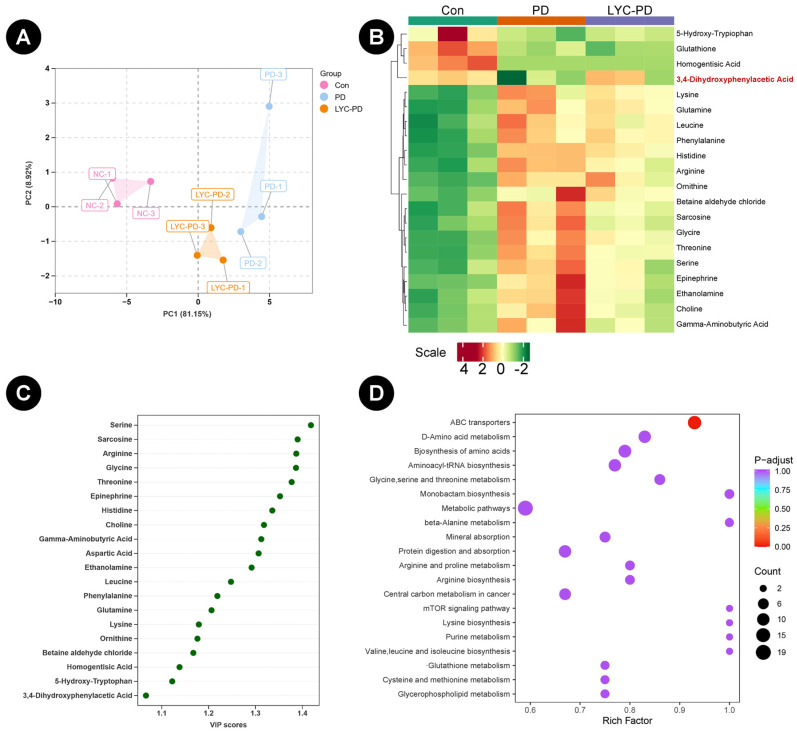
Targeted neurotransmitter metabolomics analysis. (**A**) PCA score plot. (**B**) Heatmap showing differential abundance of 21 neurotransmitters. (**C**) VIP score plot. (**D**) KEGG pathway enrichment analysis. *n* = 3 biological replicates per group for targeted neurotransmitter metabolomics; FDR-adjusted *p* < 0.05 for statistical significance.

Neurotransmitters play an important role in many physiological processes, including motor function, cognitive learning, memory, emotional regulation, and so on. Neurotransmitter heterogeneity is often closely related to the occurrence and development of neurodegenerative diseases such as PD. Targeted neurotransmitter metabolomics was performed with *n* = 3 biological replicates per group. Neurotransmitters play an important role in many physiological processes, including motor function, cognitive learning, memory, emotional regulation, and so on. Figure 6 shows the analysis results of targeted neurotransmitter metabonomics. As shown in Figure 6A, the PCA score plot revealed tight clustering of samples within each group, indicating good intra-group reproducibility. The intergroup sample discrimination is good, and there are significant metabolic differences among the groups. The results showed that there was a significant deviation between the PD group and the Con group, while the LYC-PD group approached the Con group. Figure 6B identifies 21 differentially expressed neurotransmitters in midbrain tissues of murine models, with Figure 6C presenting those meeting the VIP threshold >1. 3,4-Dihydroxyphenylacetic acid (DOPAC), a major DA metabolite, plays a pivotal role in regulating oxidative stress and neuronal signaling. As the main metabolite of DA, the level of DOPAC can reflect the integrity of the DA system. In PD mice, DOPAC levels were significantly decreased, which may be related to the degeneration of DA neurons, and the decline of DOPAC levels was significantly reduced after LYC supplementation, which may have a neuroprotective effect by regulating DA metabolism. Detailed statistical parameters of significantly differential metabolites are provided in Table S3. Figure 6D shows the KEGG classification bar chart of metabolites with significant differences. After the KEGG enrichment analysis, it was found that there were multiple metabolic pathways associated with target neurotransmitter metabolomics. In the analysis of KEGG pathway classification, differential metabolites are mainly concentrated in the categories of metabolism and human disease. Notably, the tyrosine metabolism pathway (metabolism category) and the dopaminergic synapse pathway (organismal system category) are closely related to DOPAC and significantly enriched. The former is the direct metabolic pathway of DOPAC, while the latter is the physiological site where DOPAC exerts its function. The above results suggest that LYC intervention may be related to the tyrosine–DA metabolic network and interfere with the DA neurotransmission system, which provides a new context for the analysis of the neuroprotective mechanism.

### 3.6. Effect of LYC on Midbrain Gene Expression in PD Mice

**Figure 7:**
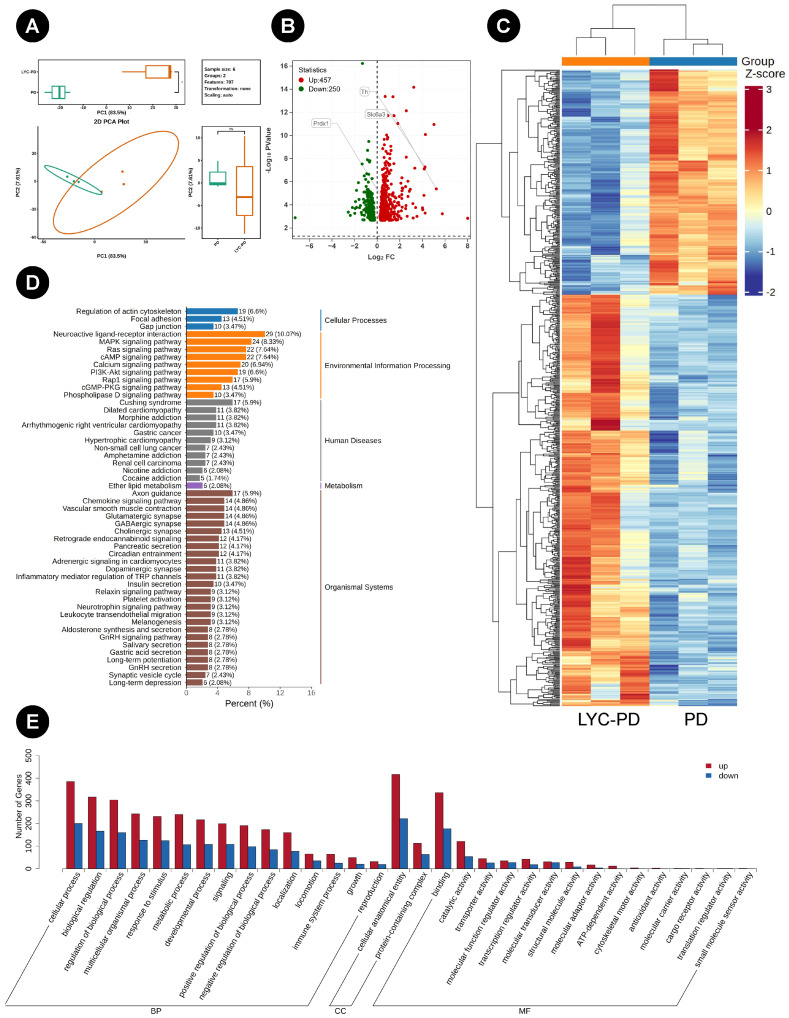
Transcriptomic analysis. (**A**) PCA score plot. (**B**) Volcano plot illustrating differentially expressed genes (DEGs). (**C**) Heatmap displaying the expression profiles of DEGs. (**D**) KEGG pathway enrichment analysis. (**E**) GO enrichment analysis. GO terms were categorized into three main branches: Biological Process (BP) refers to the series of biological events carried out by gene products; Cellular Component (CC) represents subcellular structures and cellular locations where genes exert functions; Molecular Function (MF) describes the biochemical molecular activities of gene products. *n* = 3 biological replicates per group for RNA-seq; FDR-adjusted *p* < 0.05 was considered statistically significant. Asterisks denote comparison with the PD group, * *p* < 0.05, and ns means no significant difference.

To explore the potential biological functions of LYC, we performed RNA sequencing (RNA-seq) analysis on midbrain tissues from mice in the Con, PD, and LYC-PD groups. RNA-seq transcriptomic analysis was performed with *n =* 3 biological replicates per group. Transcriptomic PCA (Figure 7A) revealed that PC1 and PC2 cumulatively accounted for 91.11% of the total expression variance. The PD and LYC-PD groups exhibited distinct separation along PC1 with tight intra-group clustering, indicating that the interventions induced substantial alterations in genome-wide transcription, accompanied by excellent sample reproducibility and reliable downstream differential expression analysis. Differential expression analysis of the transcriptomic data (Figure 7B,C) identified 707 differentially expressed genes (DEGs) between the PD and LYC-PD groups, with 457 genes significantly upregulated and 250 genes downregulated. Detailed statistical parameters of significantly differentially expressed genes are listed in Table S4. Integrated with metabolomic results, the pathways exhibiting significant metabolic alterations were all associated with the SLC transporter superfamily. Further analysis revealed the top five most significantly altered SLC transporters, including Slco2a1 (FDR *p*-value = 7.4 × 10^−13^), Slc17a7 (FDR *p*-value = 1.1 × 10^−11^), DAT/SLC6A3 (FDR *p*-value = 4.1 × 10^−6^), Slc45a3 (FDR *p*-value = 1.0 × 10^−5^), and Slc12a2 (FDR *p*-value = 1.3 × 10^−5^) (Table S5). Notably, DAT/SLC6A3, which is responsible for the reuptake of DA from the synaptic cleft into dopaminergic neurons, may contribute to synaptic DA imbalance and accelerated neuronal degeneration when dysfunctional. Transcriptomic results demonstrated that the LYC-PD group significantly upregulated DAT/SLC6A3 expression levels. The upregulation of DAT/SLC6A3 expression in the LYC-PD group serves as a highly positive and significant molecular indicator of LYC’s neuroprotective effects. This suggests that LYC may not only preserve the structural integrity of dopaminergic neurons but may also help restore their critical function: DA reuptake. As a natural dietary supplement, LYC demonstrates promising potential for auxiliary intervention or prevention in PD.

KEGG pathway enrichment and GO analyses further elucidated the functional mechanisms of the differentially expressed genes. The KEGG analysis (Figure 7D) showed that the differentially expressed genes were significantly enriched in pathways related to neural regulation, signal transduction, and metabolism. Among these, the neuroactive ligand–receptor interaction pathway (10.1%) exhibited the highest degree of enrichment, suggesting that LYC may influence PD progression by modulating synaptic transmission. Enrichment in environmental information processing-related pathways, including MAPK (8.3%), Ras (7.6%), and cAMP (7.6%) signaling, indicated that LYC is involved in the regulation of cell proliferation and apoptosis, providing pro-survival signals that help neurons resist degenerative changes. The enrichment of the ether lipid metabolism pathway (2.1%) among metabolic pathways may be associated with its neuroprotective effects. Enriched pathways linked to human diseases, such as cardiomyopathy and addiction pathways, may reflect molecular intersections with PD comorbidities. The activation of cytoskeleton regulation (6.6%) and focal adhesion (4.5%) pathways suggests that LYC may improve neuronal structural stability. Collectively, these findings reveal that LYC not only directly enhances DA reuptake by upregulating the DAT/SLC6A3 but also exerts neuroprotective effects that alleviate PD pathology through the coordinated modulation of three key molecular networks: neurotransmission, pro-survival signaling, and cytoskeletal stability, thereby acting at a systemic level.

To elucidate the molecular basis of LYC action, we performed GO functional enrichment analysis on differentially expressed genes (Figure 7E). The results revealed that the intervention effect of LYC exhibited significant coordinated changes across the three categories: biological process, cellular component, and molecular function. At the biological process level, the differentially expressed genes were predominantly enriched in categories such as cellular processes, biological regulation, and response to stimulus, with upregulated genes being predominant. This indicates that LYC can broadly activate biological programs related to cellular homeostasis maintenance and stress response. In terms of cellular components, gene products were significantly concentrated in the “cellular anatomical entity” category, suggesting that the primary targets of LYC are localized to key subcellular structures such as the cell membrane, organelles, and cytoplasmic matrix. This provides a structural basis for explaining its effects on maintaining neuronal morphology and synaptic integrity. Regarding molecular function, the differentially expressed genes were mainly involved in “binding” and “catalytic activity” functions, particularly those associated with ATP-dependent activities. This implies that LYC may exert its subtle regulatory roles by modulating specific protein–protein interactions and the activities of key enzymes, such as kinases or antioxidant enzymes. In summary, GO analysis systematically demonstrates from a functional perspective that the neuroprotective effect of LYC stems from its multi-level coordinated regulation of molecular interactions, subcellular structural integrity, and core cellular activities. These findings are consistent with and corroborate the results from transcriptomic and KEGG pathway analyses.

### 3.7. Effect of LYC on the Expression of Key Proteins in PD Mice

**Figure 8:**
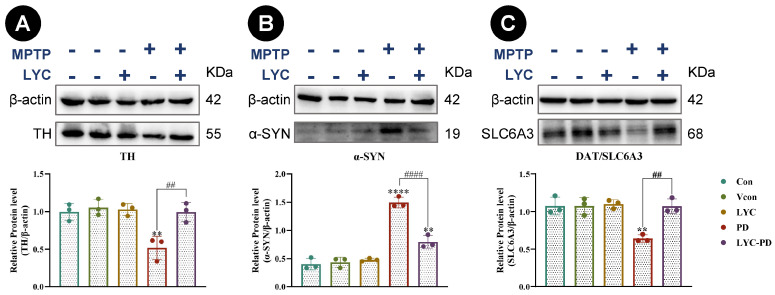
Western blot analysis of protein expression. (**A**) Protein levels of TH. (**B**) Protein levels of α-SYN. (**C**) Protein levels of DAT/SLC6A3. *n* = 3 biological replicates per group for Western blot assays. Asterisks (*) represent comparisons with the Con group: ** *p* < 0.01, **** *p* < 0.001. Hashes (#) denote comparisons with the PD group: ## *p* < 0.01, #### *p* < 0.0001.

To validate the results of our prior omics analyses at the protein level, we performed quantitative analysis of core proteins within the midbrain dopaminergic pathway using Western blot. Western blot validation was performed with *n* = 3 biological replicates per group. Protein expression was normalized to β-actin and presented as relative intensity ratios. As shown in Figure 8A and Figure S1, MPTP administration significantly suppressed the expression of tyrosine hydroxylase (TH), the rate-limiting enzyme in DA biosynthesis, compared to the Con group. LYC intervention significantly restored TH expression levels. The neurodegenerative marker α-synuclein (α-SYN) was markedly upregulated in the PD group compared to controls, and this increase was significantly attenuated by LYC treatment (Figure 8B and Figure S2). Furthermore, MPTP exposure substantially reduced SLC6A3 expression, while LYC intervention notably increased DAT/SLC6A3 expression levels (Figure 8C and Figure S3). These findings demonstrate that LYC alleviates MPTP-induced dysregulation of dopaminergic proteostasis through the coordinated modulation of TH, α-SYN, and DAT/SLC6A3. The results corroborate the therapeutic potential of LYC as a neuromodulatory agent. Specifically, our data underscore LYC’s capacity to maintain the homeostasis of dopaminergic transport proteins, suggesting that its neuroprotective efficacy may originate from its multifaceted regulation of neuronal salvage mechanisms.

### 3.8. The Results of Molecular Docking and Molecular Dynamics Simulations Between LYC and the DAT/SLC6A3 Protein

**Figure 9:**
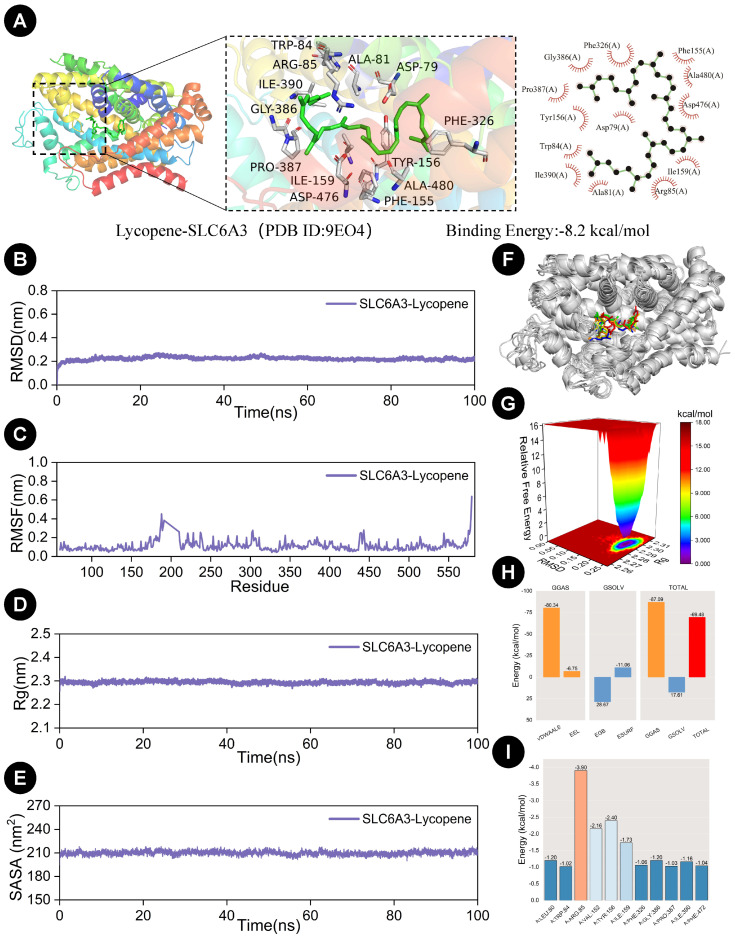
Molecular dynamics simulation of LYC binding to the DAT/SLC6A3 protein. (**A**) Molecular docking and 100 ns molecular dynamics simulation of LYC-SLC6A3 complex. The protein structure of SLC6A3 was displayed with rainbow gradient coloring in PyMOL, where different colors only distinguish sequential polypeptide segments without specific biological implications. The green stick model represents LYC ligand, and gray sticks denote key amino acid residues in the binding pocket. (**B**) Root mean square deviation (RMSD) trajectories.The RMSD curve of the LYC-SLC6A3 complex fluctuated within 1 nm throughout the simulation. The curve plateaued after 10 ns equilibration and showed no drastic fluctuations from 10 to 100 ns, demonstrating favorable structural stability of the complex from the RMSD perspective. (**C**) Root mean square fluctuation (RMSF) analysis.The RMSF curve of the LYC-SLC6A3 complex fluctuated within 1 nm without obvious drastic changes. These results revealed that lycopene exerted minimal influence on the stability of amino acid residues in SLC6A3, and the formed complex exhibited excellent stability. (**D**) Radius of gyration (Rg) profiles. The Rg curve of the LYC-SLC6A3 complex fluctuated around 2.3 nm and remained steady without obvious drastic shifts during the whole simulation. This suggested that lycopene bound tightly and stably to SLC6A3, and the addition of lycopene did not induce significant overall structural changes of the protein. (**E**) Solvent-accessible surface area (SASA) variations.The SASA curve of the LYC-SLC6A3 complex remained stable throughout the simulation with minor fluctuations around 210 nm^2^, reflecting high stability of the complex. (**F**) Complex conformations at representative time points.Structural comparison of the lycopene-SLC6A3 complex at five simulation time points (0, 25, 50, 75 and 100 ns). The small molecules in red, green, blue, yellow and orange represent the conformations of lycopene at 0, 25, 50, 75 and 100 ns, respectively. (**G**) Free energy landscape. Dark purple/blue spots in the figure represent the minimum energy states, corresponding to the most stable conformations; in contrast, red/yellow spots indicate unstable structures. A distinct low-energy cluster was observed in the free energy landscape of the LYC-SLC6A3 complex, which further verified the high stability of this complex. (**H**) Key residue contacts. VDWAALS, EEL, EGB, ESURF, GGAS, GSOLV and TOTAL stand for van der Waals energy, electrostatic energy, polar solvation energy, nonpolar solvation energy, gas-phase molecular mechanics energy, solvation free energy and total average binding free energy, respectively. (**I**) Energy contribution analysis of key amino acid residues of SLC6A3 protein binding to LYC. LYC can stably bind to the ARG-85 residue of the SLC6A3 protein with a binding energy of −3.90 kcal/mol, indicating that ARG-85 serves as a key amino acid site for their interaction. Moreover, the binding site is highly consistent with the results of molecular docking force analysis, verifying the stable binding position between the small molecule and the protein and the high reliability of the simulation results.

The molecular docking results (Figure 9A) revealed a binding energy of −8.2 kcal/mol between DAT/SLC6A3 and LYC, indicating a high binding affinity of LYC for the DAT/SLC6A3 protein. Visualization of the molecular docking conformation demonstrated that LYC forms multiple hydrophobic interactions (depicted as red eyelashes) with key amino acid residues in the DAT/SLC6A3 binding pocket, including ALA-81, TRP-84, ARG-85, and PHE-155. These extensive hydrophobic engagements suggest that DAT/SLC6A3 is likely to influence the structural conformation and biological activity of LYC, potentially modulating its functional properties through this specific binding pattern.

In order to further analyze the stability of LYC binding to DAT/SLC6A3 and analyze the interaction between the two, a molecular dynamics simulation was carried out for 100 ns. The root mean square deviation (RMSD) curve is a key indicator for evaluating the stability of protein ligand complexes. As shown in Figure 9B, the RMSD trajectory of the DAT/SLC6A3-LYC complex remained within 1.0 nm throughout the entire simulation process. After a 10 ns equilibrium period, the system achieved a stable trajectory with minimal fluctuations during the 10–100 ns production phase. From the above analysis, we can know that the complex has good stability, and its conformation is relatively stable under dynamic conditions.

The root mean square fluctuation (RMSF) curve reflects the degree of conformational flexibility of individual amino acid residues in a protein during molecular dynamics simulations. As shown in Figure 9C, the RMSF profile of the DAT/SLC6A3-LYC complex exhibited residue-wise fluctuations within 1.0 nm, with no pronounced peaks observed across the protein structure. The root mean square fluctuation of each residue is small, which indicates that the binding of LYC to the DAT/SLC6A3 protein has little impact on the conformational dynamics of the protein, and the protein structure remains stable during dynamic simulation.

The radius of gyration (Rg) can reflect the stability of the structure. According to Figure 9D, the Rg trajectory of the complex was no more than 2.3 nm, exhibiting minimal variation throughout the 100 ns simulation. The results demonstrate that LYC binding induces no significant structural perturbation in DAT/SLC6A3, with the complex maintaining a tightly packed and conformationally stable architecture.

The solvent accessible surface area (SASA) is one of the factors studied in protein structural folding and stability. Structurally stable proteins often exhibit more stable SASA curves. Figure 9E shows that the SASA curve fluctuations of the DAT/SLC6A3 protein–LYC complex remained stable throughout the entire process, with no significant variations. The fluctuation range was approximately 210 nm^2^, indicating high stability of the complex.

Conformational structures captured at five time points were compared to assess the stability of the LYC–DAT/SLC6A3 complex during molecular dynamics simulations. As shown in Figure 9F, LYC consistently binds to the same site of the DAT/SLC6A3 protein at 0, 25, 50, 75, and 100 ns without significant positional shifts, indicating high binding stability of the complex. In the free energy landscape of the DAT/SLC6A3-LYC complex (Figure 9G), a minimum energy cluster was formed, further demonstrating the excellent stability of the complex formed between the DAT/SLC6A3 protein and LYC.

After the system reached equilibrium, the MM/GBSA method was employed to calculate the average binding free energy between the DAT/SLC6A3 protein and LYC. As shown in Figure 9H, the average binding free energy of the DAT/SLC6A3-LYC complex was determined to be −69.48 kcal/mol, suggesting a strong binding affinity between the two entities.

Figure 9I reveals that LYC forms a strong interaction with the amino acid residue ARG-85 in DAT/SLC6A3, with a binding energy of −2.90 kcal/mol, indicating that ARG-85 plays a critical role in mediating the binding between LYC and the protein. Notably, the binding sites identified here align with those observed in the molecular docking interaction analysis, confirming that the binding position of the small molecule to the protein remains consistent throughout simulation and exhibits high binding stability.

### 3.9. Validation of LYC Binding to DAT/SLC6A3

**Figure 10:**
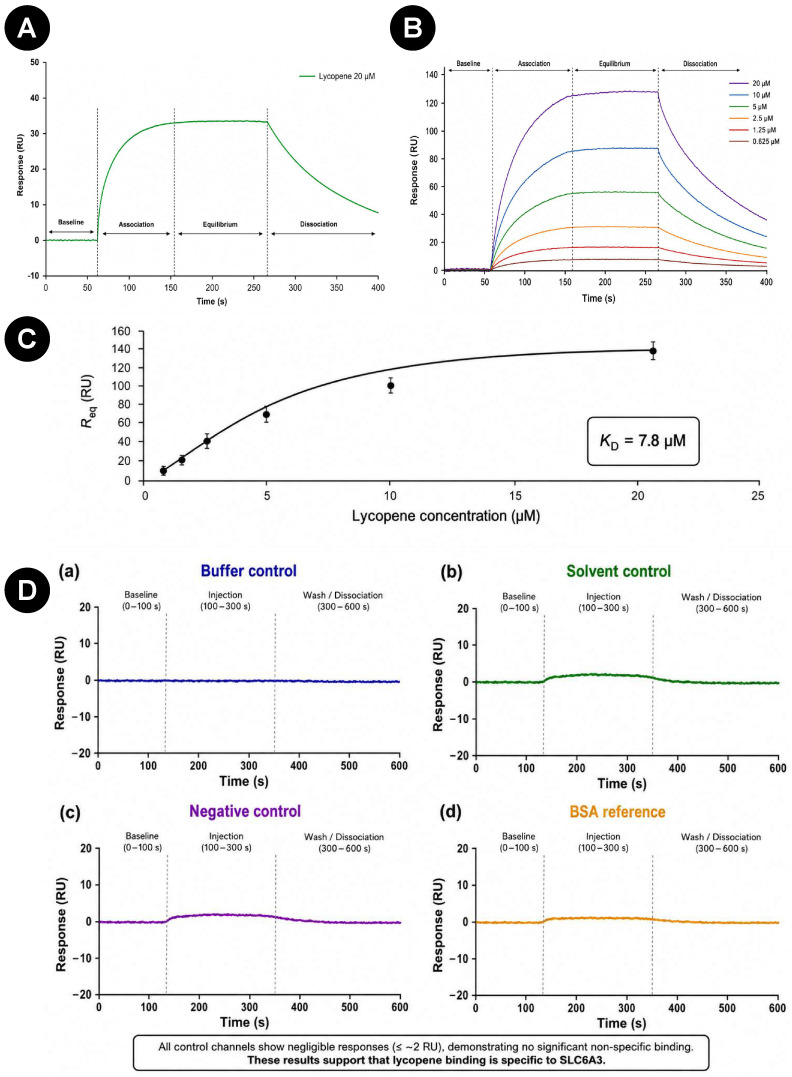
Validation of LYC binding to DAT/SLC6A3. (**A**) Sensorgram of LYC-DAT/SLC6A3. (**B**) Multiple concentrations overlay sensorgram of LYC-DAT/SLC6A3. (**C**) Equilibrium binding curve of LYC-DAT/SLC6A3. (**D**) Specificity assay plot of LYC binding to DAT/SLC6A3. (**a**) Buffer control; (**b**) Solvent control; (**c**) Negative protein control; (**d**) BSA reference channel. All controls exhibited negligible SPR signals, ruling out bulk refractive interference and non-specific binding of LYC. *n* = 3 independent chip replicates for SPR detection; all data were analyzed following uniform statistical and significant digit standards.

SPR binding assays were performed with *n* = 3 independent chip biological replicates to verify the direct interaction between LYC and the DAT/SLC6A3 protein. The direct interaction between LYC and the DAT/SLC6A3 protein was verified using SPR. Figure S5 shows the global dynamic fitting and parallel experimental validation of lycopene and DAT/SLC6A3. The gradient concentration fitting results showed that the two followed the classic 1:1 binding mode, with three highly overlapping independent repeat curves and stable binding kinetic parameters, confirming that lycopene can specifically bind to DAT/SLC6A3 and that the detection system has good repeatability and reliability. Figure 10A shows the sensorgram of LYC-DAT/SLC6A3. The results show that LYC can specifically bind to the DAT/SLC6A3 protein immobilized on the sensor chip, and there is a significant signal response change during the binding process, with a stable decrease in signal during the dissociation stage. Figure 10B shows the multiple concentrations overlay sensorgram of LYC-DAT/SLC6A3. The results showed that the DAT/SLC6A3 multiple concentrations curve had the highest response at 20 µM and exhibited a dose-dependent effect. Figure 10C shows the equilibrium binding curve of LYC-DAT/SLC6A3. The results showed that the steady-state affinity analysis of DAT/SLC6A3 yielded a KD of approximately 7.8 µM, indicating moderate binding affinity. Figure 10D shows the specificity assay plot of LYC binding to DAT/SLC6A3. The four groups of DAT/SLC6A3 control signals remained within the low RU range, and no effective binding was observed, confirming that LYC specifically binds to DAT/SLC6A3.

## 4. Discussion

PD is a common neurodegenerative disorder characterized by the progressive loss of dopaminergic neurons in the substantia nigra of the midbrain, leading to a significant reduction in striatal DA levels and subsequent motor and non-motor symptoms [40,41]. Currently, DA replacement therapy is the main treatment in clinical practice, especially levodopa therapy; however, long-term use is often associated with complications, drug tolerance, and dyskinesia [42,43,44]. In this context, it is imperative to identify additional natural compounds that can exert neuroprotective effects in preventing the deterioration of the disease. LYC, a potent natural antioxidant, has demonstrated protective potential in various models of neurological disorders [45,46,47]. Nevertheless, in the treatment of PD, the specific mechanism of its action has not been fully elucidated, and whether it can maintain the stability of the dopaminergic system remains unknown. To address this research gap, we combined behavioral assays, histopathology, multi-omics profiling, and computational simulations. This study assessed LYC’s neuroprotective activity in an MPTP-induced PD mouse model and explored its molecular target and downstream signaling networks.

This study evaluated the protective effects of LYC on the MPTP-induced mouse model of PD at the whole-animal level. The results demonstrated that LYC could alleviate the decline of body weight and brain weight ratio caused by MPTP, which was enough to show that LYC could mitigate the decline of systemic physiological functions of PD model mice, and had a positive effect on the occurrence of neuroprotective action. This constitutes a preliminary observation regarding the physiological basis of LYC’s efficacy. More behavioral tests were carried out in this study. It was found that LYC could effectively relieve the motor dysfunction of PD model mice, alleviate anxiety-like behaviors, and improve the hypo-exploratory phenotype in PD model mice. Although these parameters did not fully restore to control levels, these data substantiate the capacity of LYC to alleviate core behavioral phenotypes in PD. In the process of behavior improvement, the results of histopathological analysis can reflect the importance of evidence support. In this study, it was found that LYC intervention could play a role in protecting neurons, and the injury to the midbrain could be alleviated, which could not only relieve vacuolation but also reduce the occurrence of nuclear pyknosis and abnormal morphology. Upon Nissl staining, it was found that LYC could help neurons maintain the number of Nissl bodies, and the distribution was relatively uniform, which also indicated that neurons could maintain a certain synthetic function.

Multi-omics analysis revealed the mechanism by which LYC regulates the homeostasis of dopaminergic neurons. Transcriptome analysis identified significant differential expression of the key DA transporter DAT/SLC6A3. This alteration has also been validated at the protein level. The upregulation of DAT/SLC6A3 by LYC corresponds coherently with the recovery of TH expression—the rate-limiting enzyme in DA synthesis—and the improvement in levels of the key metabolite DOPAC. Dysregulated midbrain amino acids and monoamine neurotransmitters are recognized as characteristic pathological biomarkers of neurodegeneration. Systematic metabolomic detection of these small molecules, especially the DA metabolite DOPAC, is commonly used to decipher metabolic disorders associated with dopaminergic neuron impairment [48,49]. Together, these findings illustrate that LYC likely repairs and stabilizes dopaminergic neurotransmission at a systemic level by coordinately modulating multiple key steps in the DA pathway: “synthesis (TH)—release—reuptake (DAT/SLC6A3)—metabolism (DOPAC).” However, the upregulation of DAT/SLC6A3 is not only a direct reflection of restored dopaminergic function but also acts as a critical node that integrates and activates broader neuroprotective and homeostatic programs [50,51,52]. Transcriptomic KEGG analysis revealed that LYC significantly enriched pro-survival signaling pathways such as MAPK, Ras, and cAMP. These pathways not only directly inhibit apoptosis and promote neuronal survival but also provide the necessary intracellular signaling environment for DAT/SLC6A3 upregulation. Meanwhile, the remodeling of glycerophospholipid and arachidonic acid metabolism, revealed by untargeted metabolomics, helps optimize neuronal membrane fluidity, integrity, and signal transduction efficiency. This may furnish the essential lipid microenvironment for the proper membrane insertion, stable presence, and transport function of the DAT/SLC6A3 protein. Located on the presynaptic membrane of dopaminergic neurons, DAT/SLC6A3 is responsible for reuptaking DA from the synaptic cleft back into the neuron, playing a critical role in precisely regulating the duration and intensity of dopaminergic signaling [53,54]. In the early stages of PD, impaired or deficient DAT/SLC6A3 function leads to abnormal accumulation of DA in the synaptic cleft, exacerbating oxidative stress and excitotoxicity, thereby accelerating neuronal degeneration [55,56]. Thus, the LYC-induced upregulation of DAT/SLC6A3 may not only directly enhance DA reuptake efficiency and restore the dynamic balance of synaptic DA but also serve as an important indicator of the maintained functional integrity of dopaminergic neurons. Effective DAT/SLC6A3 operation relies on healthy synaptic structure [57]. The enrichment of pathways such as “neuroactive ligand-receptor interaction” and “focal adhesion” suggests that LYC can improve postsynaptic signal reception and cell–matrix adhesion, collectively maintaining overall synaptic structural and functional integrity. The fact that LYC can play the role of “neuroactive ligand” in this way makes it possible to improve cell–matrix adhesion and promote the reception of postsynaptic signals, which is an important prerequisite for the maintenance of synaptic function and the improvement of synaptic structure. It can also lay the foundation for the rapid reuptake of DA through DAT/SLC6A3. Moreover, the expression of DAT/SLC6A3 and TH can be restored at the same time to make the synthesis and reuptake of DA synchronized, so that DA will not accumulate abnormally in the synaptic cleft and will not be exhausted, and the homeostasis of DA can be restored [58]. Collectively, LYC can regulate the expression of DAT/SLC6A3 and reshape the membrane lipid microenvironment to maintain the activity of transport proteins, thereby improving DA metabolism abnormalities.

In addition, computational biology provides key evidence that LYC can interact with DAT/SLC6A3 at the atomic level. Molecular docking revealed that LYC can embed into a specific hydrophobic pocket of the DAT/SLC6A3 protein with high binding affinity (docking score: –8.2 kcal/mol), forming stable hydrophobic interactions with key residues such as ALA-81 and ARG-85. Molecular dynamics simulation comprehensively evaluated the dynamic stability of the complex: the stable RMSD, low RMSF, compact RG, and smooth SASA curve showed that the LYC–DAT/SLC6A3 complex maintained a stable conformation in the simulated environment and did not induce severe disturbance of protein structure. Binding free energy calculation results show that the interaction has high thermodynamic feasibility (MM/GBSA result: −69.48 kcal/mol). These data predicted the direct and stable physical binding between LYC and the DAT/SLC6A3 protein. Nevertheless, the docking result merely represents static interactions under computational simplifications and fails to recapitulate the dynamic binding features in real physiological conditions. This represents an inherent limitation of the current docking analysis for membrane transporter proteins. To further verify the reliability of theoretical predictions, this study conducted SPR in vitro binding experiments. The concentration gradient of LYC showed a regular binding curve, and the interaction was concentration-dependent. The steady-state affinity analysis yielded a KD of 7.8 µM, directly confirming the direct and specific in vitro interaction between LYC and DAT/SLC6A3.

This finding provides a complementary explanation for the aforementioned multi-omics and immunoblotting results. Numerous prior studies have validated the neuroprotective potency of lycopene in MPTP- and rotenone-induced PD animal models, with most investigations focusing primarily on antioxidative, anti-inflammatory, and behavioral improvements. Although these findings support the protective effects of LYC, the underlying molecular regulatory pathways and core target mechanisms contributing to DA homeostasis remain to be further elaborated upon [20,59]. We observed that LYC intervention not only significantly upregulated DAT/SLC6A3 expression at both the transcriptional and protein levels but may also directly bind to the DAT/SLC6A3 protein, acting in a manner analogous to a “molecular chaperone” or pharmacological chaperone. Such binding could stabilize the native conformation of DAT/SLC6A3 and reduce its misfolding or ubiquitin-mediated degradation, thereby enhancing DAT/SLC6A3 protein stability and prolonging its half-life, ultimately leading to cumulative increases in protein expression at the cellular level. Distinct from previous phenotypic observations, the present study established a multi-layered research framework integrating multi-omics screening, molecular dynamics simulation, and in vitro protein–ligand interaction validation to identify DAT/SLC6A3 as a core target of lycopene. We further preliminarily clarified that LYC maintains DAT/SLC6A3 expression and functional stability through dual regulatory mechanisms, including transcriptional modulation and pharmacological chaperone-like post-translational stabilization. This study complements the current understanding of lycopene’s neuroprotective actions and provides experimental and theoretical support for future in-depth mechanistic exploration in PD research. In other words, LYC may influence DAT/SLC6A3 abundance not only by regulating gene transcription but also by stabilizing and protecting DAT/SLC6A3 at the post-translational level through direct protein–ligand interaction.

Specifically, LYC not only ameliorates motor deficits and midbrain pathological damage in PD mouse models at a holistic level but also specifically upregulates the expression and function of DAT/SLC6A3, potentially through direct and stable physical interaction akin to a “pharmacological chaperone” that stabilizes DAT/SLC6A3. Meanwhile, LYC broadly modulates tyrosine metabolism, activates pro-survival signaling pathways, preserves synaptic and cytoskeletal integrity, and reshapes the lipid metabolic profile. Thus, the upregulation of DAT/SLC6A3 function directly strengthens the core process of dopaminergic signaling, whereas the extensive neuroprotective and metabolic adjustments provide the essential molecular environment and systemic support for the stable expression and functioning of DAT/SLC6A3 and even the survival of dopaminergic neurons. Although this study yielded a series of findings, certain limitations remain. Future studies will focus on how LYC specifically upregulates DAT/SLC6A3 expression, investigating the upstream signal transduction pathways and key transcription factors involved.

Most previous studies have confirmed the neuroprotective effects of LYC in PD models such as MPTP and rotenone, but related research has mostly been limited to macroscopic phenotype observations, such as antioxidant, anti-inflammatory, and behavioral improvement, lacking systematic molecular mechanism elucidation and core target identification, and the key regulatory cascade governing dopamine homeostasis has not been clarified. Unlike previous single-phenotype studies, the innovative value of this study lies not only in demonstrating the targeted regulation of SLC6A3 by LYC but also in a multidimensional research framework through multiomics analysis, molecular dynamics simulation, and in vitro interaction assays. At the same time, this study preliminarily revealed that LYC can maintain the expression and functional stability of the SLC6A3 protein through dual pathways of transcriptional regulation and pharmacological chaperoning, which, to some extent, addresses the shortcomings of previous research on the relatively single mechanism and unclear regulatory network. It can provide certain experimental references and theoretical insights for further in-depth analysis of the molecular mechanism of LYC in combating PD.

Although this study yielded a series of findings, certain limitations remain. The study used controllable gavage administration and a single experimental dose, which differed from the daily dietary intake pattern of the human body. Meanwhile, LYC has strong lipophilicity, limited blood–brain barrier penetration efficiency, and effective brain exposure, and animal experimental doses cannot directly correspond to human equivalent doses. In addition, the experiment only simulates subacute neuronal injury and cannot fully replicate the pathological characteristics of the chronic progression of Parkinson’s disease in humans. Further improvement of the administration method and dosage exploration can be carried out in the future to verify its in vivo bioavailability and brain exposure level, thereby continuously supplementing its application potential. In addition, we will further explore the specific molecular mechanism of LYC upregulating SLC6A3 expression and delve into the upstream signaling pathways and key transcription factors involved in its regulation.

## 5. Conclusions

LYC treatment relieved motor impairment and reduced midbrain pathological injury in our PD mouse model, demonstrating prominent neuroprotective activity. We found that LYC increased the expression of DAT/SLC6A3 and formed direct physical binding with the DAT/SLC6A3 protein. This interaction could stabilize the DAT protein through a pharmacological chaperone-like effect, which may support the balance of dopamine synthesis, reuptake, and metabolism. Apart from modulating DAT/SLC6A3, LYC also protected the synaptic structure and cytoskeleton. It activated pro-survival signaling pathways and remodeled lipid metabolism to maintain neuronal integrity. All these biological changes centered on DAT/SLC6A3 may jointly account for the overall neuroprotection afforded by lycopene against PD neuronal damage. On the basis of these in vivo and molecular observations, SLC6A3/DAT emerges as a promising molecular target associated with LYC’s protective effect. Further functional experiments are still required to clarify the precise regulatory mechanism.

## Data Availability

The data supporting this article are included as part of the Supplementary Information. The raw transcriptome sequencing data have been deposited in the NCBI GEO database (accession number: GSE336581; URL: https://www.ncbi.nlm.nih.gov/geo/query/acc.cgi?acc=GSE336581 (accessed on 26 May 2026); scheduled public release date: 24 June 2030). The full raw mass spectrometry data of untargeted metabolomics were submitted to the MetaboLights database (accession number: MTBLS14829; URL: https://www.ebi.ac.uk/metabolights/MTBLS14829 (accessed on 26 May 2026)). The complete raw mass spectrometry data for targeted neurotransmitter metabolomics were uploaded to MetaboLights (accession number: MTBLS14806; URL: https://www.ebi.ac.uk/metabolights/MTBLS14806 (accessed on 26 May 2026)).

## Supplementary Materials

The following supporting information can be downloaded at https://www.mdpi.com/article/10.3390/nu18142234/s1: Table S1: The ARRIVE guidelines 2.0: author checklist. Figure S1: Full-length Western blot image showing TH protein expression in midbrain tissues. Figure S2: Full-length Western blot image showing α-SYN protein expression in midbrain tissues. Figure S3: Full-length Western blot image showing DAT/SLC6A3 protein expression in midbrain tissues. Figure S4: Schematic layout of the 15-well SDS-PAGE gel. Figure S5: Surface plasmon resonance detection of global dynamics fitting curve and repeatability of LYC-DAT/SLC6A3. Table S2: Statistical results of all features/metabolites in untargeted metabolome differential analysis. Table S3: Statistical results of all detected metabolites in targeted metabolome differential analysis. Table S4: Statistical results of all genes in transcriptome differential analysis. Table S5: Transcriptomic profile of LYC intervention in a PD model: Upregulation of key transporter genes implicated in neuroprotective potential.

## Abbreviations

The following abbreviations are used in this manuscript:

LYC	Lycopene
PD	Parkinson’s Disease
DA	Dopamine
DAT/SLC6A3	Solute Carrier Family 6 Member 3
L-DOPA	3,4-Dihydroxyphenyl L-Alanine
NF-κB	Nuclear Factor Kappa-Light-Chain-Enhancer of Activated B Cells
IL-1β	Interleukin-1 Beta
TNF-α	Tumor Necrosis Factor Alpha
ROS	Reactive Oxygen Species
Nurr1	Nuclear Receptor Related 1 Protein
Pitx3	Paired-Like Homeodomain Transcription Factor 3
Nrf2	Nuclear Factor Erythroid 2-Related Factor 2
PI3K/Akt	Phosphoinositide 3-Kinase/Protein Kinase B
SPF	Specific Pathogen-Free
MPTP	1-Methyl-4-Phenyl-1,2,3,6-Tetrahydropyridine
OFT	Open Field Test
PT	Pole Test
TST	Tail Suspension Test
RT	Rotarod Test
H&E	Hematoxylin–Eosin
PCA	Principal Component Analysis
OPLS-DA	Orthogonal Partial Least Squares–Discriminant Analysis
VIP	Variable Importance in Projection
KEGG	Kyoto Encyclopedia of Genes and Genomes
GO	Gene Ontology
RIPA	Radioimmunoprecipitation Assay
BCA	Bicinchoninic Acid
SDS	Sodium Dodecyl Sulfate
PVDF	Polyvinylidene Difluoride
HRP	Horseradish Peroxidase
SPR	Surface Plasmon Resonance
GP	Glycerophospholipids
FA	Fatty acids
DOPAC	3,4-Dihydroxyphenylacetic Acid
RNA-seq	RNA Sequencing
DEGs	Differentially Expressed Genes
SLC	Solute Carrier
MAPK	Mitogen-Activated Protein Kinase
Ras	Rat Sarcoma Virus Protein
ATP	Adenosine Triphosphate
BP	Biological Process
CC	Cellular Component
MF	Molecular Function
TH	Tyrosine Hydroxylase
α-SYN	α-synuclein
RMSD	Root Mean Square Deviation
RMSF	Root Mean Square Fluctuation
Rg	Radius of Gyration
SASA	Solvent Accessible Surface Area
FDR	False Discovery Rate
FPKM	Fragments per Kilobase of Transcript per Million Mapped Reads
