# Supplementary material for: Neuroprotective Effects of Lycopene in Parkinson’s Disease Mice: Potential Modulation of DAT/SLC6A3-Mediated Dopaminergic Pathway
Source: Nutrients. 2026 Jul 9;18(14):2234. doi: 10.3390/nu18142234 (PMC13416145; doi:10.3390/nu18142234)
Supplement: Supplementary file 1 [file nutrients-18-02234-s001.zip › nutrients-4378203-supplementary.pdf]

Supplemental Materials

Supplementary Table S1: The ARRIVE guidelines 2.0: author checklist

Item	Recommendation	Section/line number, or reason for not reporting
Study design	For each experiment, provide brief details of study design including:<br>a. The groups being compared, including control groups. If no control group has been used, the rationale should be stated.<br>b. The experimental unit (e.g. a single animal, litter, or cage of animals).	a. Animals were randomly divided into five groups: blank control group (Con), vehicle control group (Vcon), lycopene (LYC) group, PD group, and LYC - PD combination group. Blank control C57BL/6J mice received standard chow without any intervention; vehicle control mice were given the identical solvent for dissolving LYC and PD with the same administration regimen; the LYC group was treated with lycopene alone; the PD group received single PD administration; the combined group was co-administered with lycopene and PD at respective doses. Control groups were predefined prior to animal experiments to eliminate the interference of basal feeding conditions and solvent.<br>b. A single C57BL/6J mouse was defined as one independent experimental unit.
Sample size	a. Specify the exact number of experimental units allocated to each group, and the total number in each experiment. Also indicate the total number of animals used.<br>b. Explain how the sample size was decided. Provide details of any a priori sample size calculation, if done.	a. Animals were randomly allocated into five groups, with 25 C57BL/6J mice per group. One single mouse represented one independent experimental unit. The total number of experimental animals used in this study was 125 (5 groups × 25 mice/group).<br>b. Sample size was determined referring to published literatures about lycopene against PD, combined with multiple experimental endpoints including behavioral tests, biochemistry, qPCR and western blot detection. Considering unavoidable animal loss from PD modeling mortality, accidental death and sample sacrifice for multi-index detection, we set the initial sample size as n=30 per group. After excluding dead/invalid mice during modeling and feeding, sufficient remaining animals were available to complete all experimental detections, ensuring adequate statistical power for statistical analysis and reducing type Ⅱ error.
Inclusion and exclusion criteria	a. Describe any criteria used for including and excluding animals (or experimental units) during the experiment, and data points during the analysis. Specify if these criteria were established a priori. If no criteria were set, state this explicitly.<br>b. For each experimental group, report any animals, experimental units or data	a. All inclusion and exclusion criteria for experimental animals and data points were established a priori before the start of animal modeling and formal experiments. Only healthy male C57BL/6J mice with normal mental status, regular food and water intake, and consistent body weight range were included in the study. Animals that died accidentally during feeding, died due to failure of PD model establishment, exhibited severe postoperative complications, or showed abnormal physiological conditions that interfered with experimental results were excluded from subsequent experiments and data analysis. Data points with obvious operation errors, detection failures, or unmeasurable indicator values were also excluded during statistical analysis.

	points not included in the analysis and explain why. If there were no exclusions, state so.<br>c. For each analysis, report the exact value of n in each experimental group	b. No artificial subjective elimination was performed in any experimental group. A small number of mice were excluded only due to unavoidable objective factors, including PD modeling failure, accidental death during breeding, and invalid samples caused by experimental operation errors. All excluded animals and invalid data were fully recorded. No outliers were arbitrarily removed. All valid remaining samples after objective screening were included in the final statistical analysis.<br>c. The initial animal sample size of each group was 25 C57BL/6J mice. After excluding invalid and dead individuals during modeling and feeding, the final valid sample number n of each group used for statistical analysis was consistent and sufficient for all experimental indicators. The exact n values for each group are presented in the corresponding figure legends and analysis results.
Randomisation	a. State whether randomisation was used to allocate experimental units to control and treatment groups. If done, provide the method used to generate the randomisation sequence.<br>b. Describe the strategy used to minimise potential confounders such as the order of treatments and measurements, or animal/cage location. If confounders were not controlled, state this explicitly	a. Randomisation was strictly applied to allocate experimental mice to all control and treatment groups. Before grouping, all mice were numbered sequentially according to their body weight. A simple random sampling method was used to generate the random allocation sequence via SPSS 26.0 statistical software. Mice were randomly assigned to five experimental groups in accordance with the generated random sequence to avoid subjective grouping bias and ensure that baseline physiological conditions including body weight and mental state were comparable among all groups.<br>b. Unified standardized experimental strategies were adopted to minimize potential confounding factors throughout the study. All mice were housed in identical specific pathogen-free (SPF) animal rooms with consistent ambient temperature (22–25 °C), humidity (50%±10%), and 12 h light/dark cycle. Regular diet and sterile drinking water were provided ad libitum for all groups. The administration and behavioral testing order of mice from different groups were cross-balanced and randomized daily to eliminate time-dependent differences. Cage positions were rotated regularly every three days to avoid environmental light and positional interference. All experimental operations, including modeling, drug administration, and sample detection, were performed by the same trained operator following unified protocols to ensure consistent experimental conditions.
Blinding	Describe who was aware of the group allocation at the different stages of the experiment (during the allocation, the conduct of the experiment, the outcome assessment, and the data	Blinding status was clearly defined at each experimental stage. At the allocation stage, one researcher generated the random sequence and performed the grouping and was aware of the group assignment, while all other experimenters were blinded. During experimental conduct (modeling and drug administration), blinding was not maintained because different groups required distinct treatment regimens, which made complete blinding operationally

	analysis).	impossible. During outcome assessment, including behavioral tests, biochemical and molecular measurements, all testers were fully blinded to group allocation to avoid observer bias.
Outcome measures	a. Clearly define all outcome measures assessed (e.g. cell death, molecular markers,<br>or behavioural changes).<br>b. For hypothesis-testing studies, specify the primary outcome measure, i.e. the outcome measure that was used to determine the sample size.	a. A comprehensive set of outcome measures was assessed in this study to systematically evaluate the protective effects of lycopene against Parkinson' s disease-related injuries in mice. The detected indicators covered multiple dimensions including physiological phenotypic changes, behavioral function, omics profiling, molecular biomarker expression, and cerebral pathological morphology. Specifically, the assessed outcomes included general physiological indexes (body weight changes, daily food and water intake, and organ coefficients of major organs), neurobehavioral alterations (motor and cognitive behavioral performance of mice), multi-omics profiling (untargeted tissue metabolomics, targeted neurotransmitter metabolomics, and transcriptomics analysis), neuronal biomarker expression (the expression levels of key neuronal functional and injury markers), and cerebral histopathological morphological changes.<br>b. This study was a hypothesis-testing animal experiment. The primary outcome measure predefined for sample size calculation was thebehavioral functional recovery of PD model mice, which was the core indicator for judging the protective effect of lycopene intervention. Considering the relatively large biological variation of behavioral data and the potential attrition rate of PD modeling, a relatively large sample size (n = 25 per group) was adopted to ensure sufficient statistical power for the primary outcome and supporting secondary molecular and pathological indicators.
Statistical methods	a. Provide details of the statistical methods used for each analysis, including<br>software used.<br>b. Describe any methods used to assess whether the data met the assumptions of<br>the statistical approach, and what was done if the assumptions were not met.	a. All quantitative data in this study, including physiological indexes, behavioral results, omics data, molecular biomarker expression and pathological statistical results, were analyzed by one-way analysis of variance (one-way ANOVA). Multiple comparisons between different experimental groups were performed using Tukey' s post-hoc test. All statistical analyses were conducted using SPSS 26.0 and GraphPad Prism 9.0 software. All data were presented as mean ± standard deviation (SD). A p-value < 0.05 was defined as the threshold of statistically significant difference.<br>b. Prior to one-way ANOVA analysis, Shapiro-Wilk normality test and Levene's homogeneity of variance test were performed to verify whether the data met the prerequisite assumptions of one-way ANOVA (normal distribution and homogeneous variance). All datasets involved in this study conformed to normal distribution and homogeneous variance. No data violated the statistical assumptions of the parametric test, so no additional non-parametric

		correction or data conversion was required, and one-way ANOVA was directly adopted for all analyses.
Experimental animals	a. Provide species-appropriate details of the animals used, including species, strain and substrain, sex, age or developmental stage, and, if relevant, weight.<br>b. Provide further relevant information on the provenance of animals, health/immune status, genetic modification status, genotype, and any previous procedures.	a. All experimental animals used in this study were male C57BL/6J inbred mice (substrain: C57BL/6J). The mice were aged 7–8 weeks at the beginning of the experiment, with an initial body weight range of 18–22 g. Animals with consistent age and body weight were selected to ensure uniform baseline conditions among all groups.<br>b. The experimental mice were purchased from a qualified professional laboratory animal supplier. All mice were wild-type (non-genetically modified) and maintained in a specific pathogen-free (SPF) health status. No genetic modification, drug pretreatment, or prior experimental procedures were performed on the animals before this study. All mice were acclimatized to the laboratory environment for one week before formal modeling and intervention experiments to adapt to the feeding environment and eliminate stress interference.
Experimental procedures	For each experimental group, including controls, describe the procedures in enough detail to allow others to replicate them, including:<br>a. What was done, how it was done and what was used.<br>b. When and how often.<br>c. Where (including detail of any acclimatisation periods).<br>d. Why (provide rationale for procedures).	a. This study included five experimental groups: blank control group, vehicle control group, LYC single treatment group, PD model group, and LYC + PD combined treatment group. For the PD model group and the combined group, a classic PD mouse model was established by corresponding modeling reagents and standardized modeling methods. The lycopene (LYC) intervention solution was prepared with conventional solvent, and the same solvent without LYC was used for the vehicle control group. Mice in the LYC group received intragastric administration of lycopene solution alone; the PD group received only PD modeling treatment without drug intervention; the combined group was subjected to PD modeling and simultaneous lycopene intragastric intervention; the blank control group received no modeling or drug treatment and was given normal feeding only. All experimental instruments and reagents used in modeling and administration were consistent among groups.<br>b. After one week of acclimatization, formal modeling was performed at a fixed time every day. The modeling procedure was carried out continuously according to the established model construction cycle. Drug intervention was conducted once daily at the same time point throughout the whole intervention period to ensure consistent treatment frequency and avoid circadian rhythm interference.<br>c. All animal experiments were completed in a standardized SPF-grade animal laboratory. Before the formal experiment, all mice underwent a 7-day adaptive acclimatization period under consistent housing conditions (temperature 22–25 °C, humidity 50%±10%,

		12 h light/dark cycle) with free access to food and water. All subsequent modeling, drug administration and sample collection procedures were performed in the same laboratory environment.<br>d. The grouping and intervention procedures were designed strictly based on classic PD animal models and nutritional intervention research paradigms. The blank control group reflected the physiological baseline of normal mice. The vehicle control group excluded the potential interference of solvent components on mouse physiology and experimental results. The single LYC and single PD groups were set to separately verify the independent effect of lycopene and the pathological changes induced by PD modeling. The combined group was used to explore the synergistic protective effect of LYC against PD injury. Unified operation time, environment and frequency ensured high reproducibility and eliminated external confounding factors.
Results	For each experiment conducted, including independent replications, report:<br>a. Summary/descriptive statistics for each experimental group, with a measure of variability where applicable (e.g. mean and SD, or median and range).<br>b. If applicable, the effect size with a confidence interval.	a. All quantitative data obtained in this study, including physiological indices, behavioral test results, multi-omics data, molecular biomarker expression levels, and pathological statistical results, were presented as mean ± standard deviation (SD). Standard deviation was used as the variability indicator to reflect the degree of data dispersion. All experimental groups adopted the same unified descriptive statistical method to ensure consistent data presentation. The detailed descriptive statistical results of each index in every group are shown in the corresponding figures and tables.<br>b. Effect size and confidence interval analysis was not applicable for the present study. This study was a conventional in vivo animal intervention experiment with multiple groups of comparative observation. The core statistical evaluation was based on one-way ANOVA with post-hoc multiple comparisons, which is the standard and widely recognized statistical method for nutritional intervention and animal model studies in Nutrients and related biomedical journals.

Supplementary Figure S1: Full-length Western blot image showing TH protein expression in midbrain tissues.

Western blot analysis of TH (55 kDa) and β-actin (42 kDa) proteins was performed on mouse midbrain tissues with three independent biological replicates. For each panel, the upper row shows the original images of transferred membranes, and the lower row displays images captured at different exposure times. The protein bands of β-actin and TH from the three independent replicates are arranged sequentially from top to bottom. The densitometric quantification results are consistent with those presented in Figure 8A of the main text.

WB_Rep1_β-actin

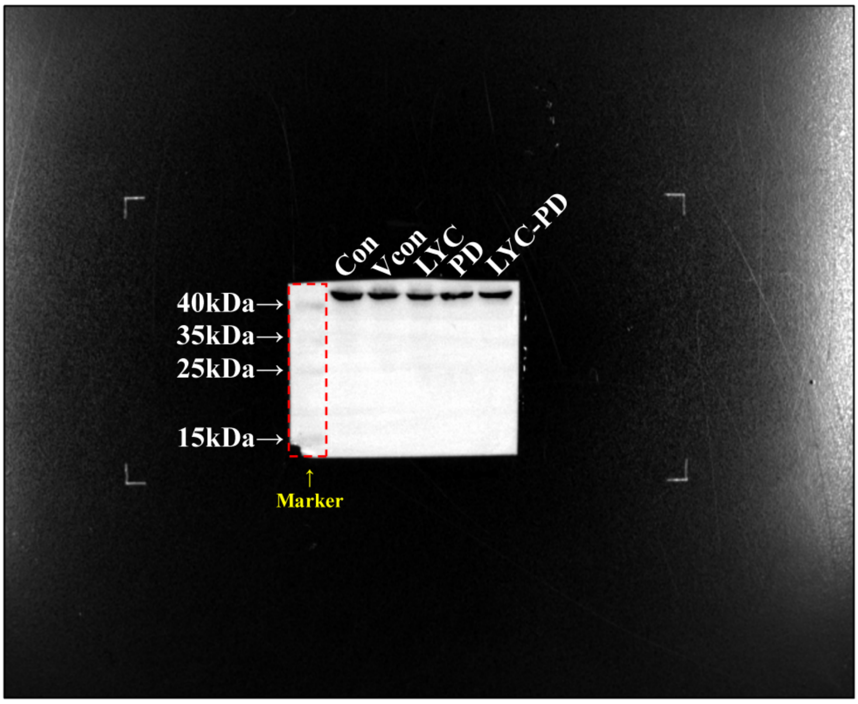

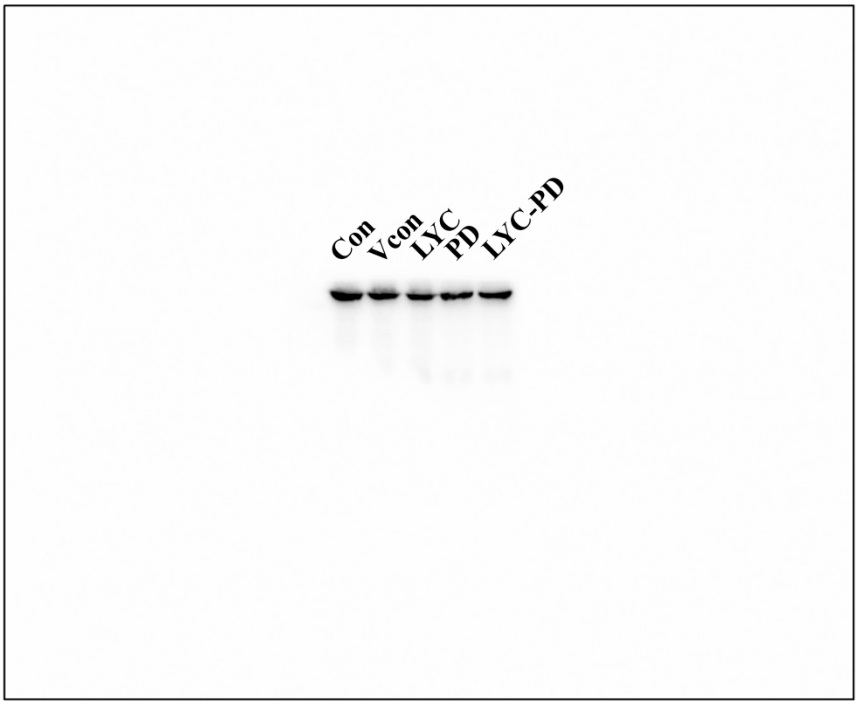

WB_Rep1_TH

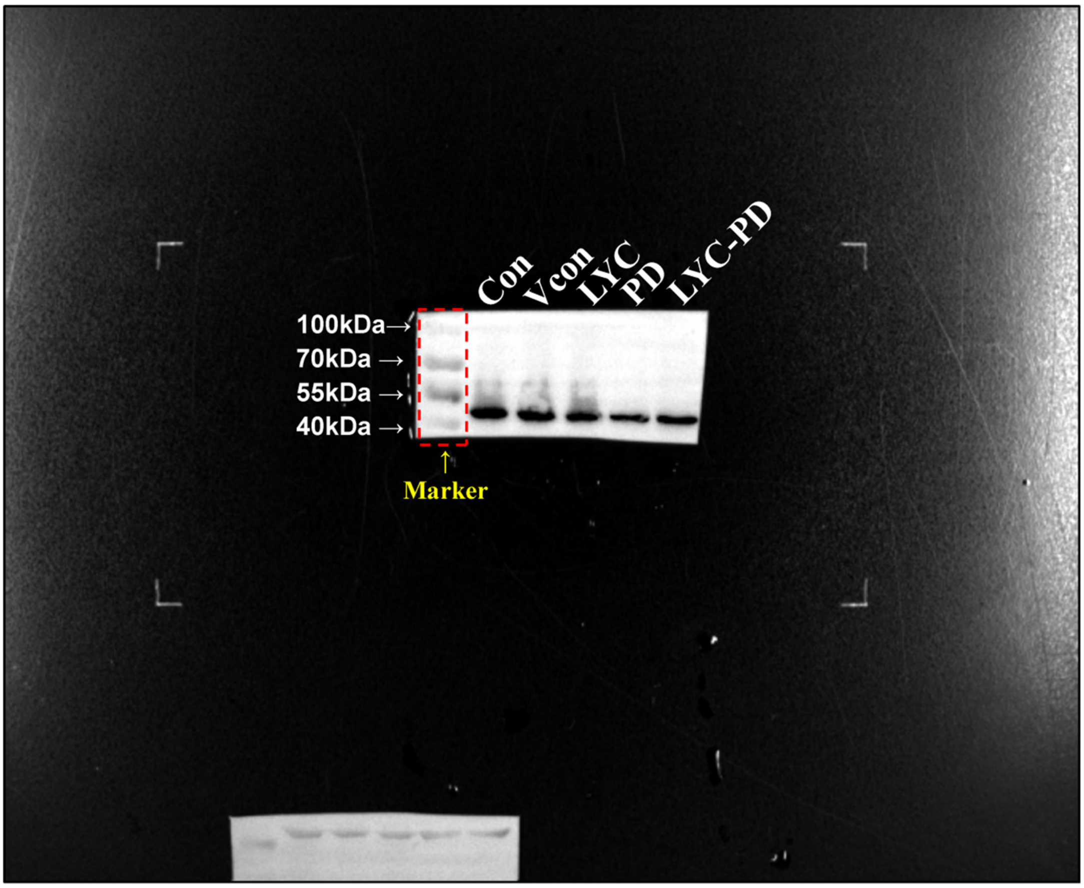

Con
Vcon
LYC
PD
LYC-PD
100kDa→
70kDa →
55kDa →
40kDa →
↑
Marker

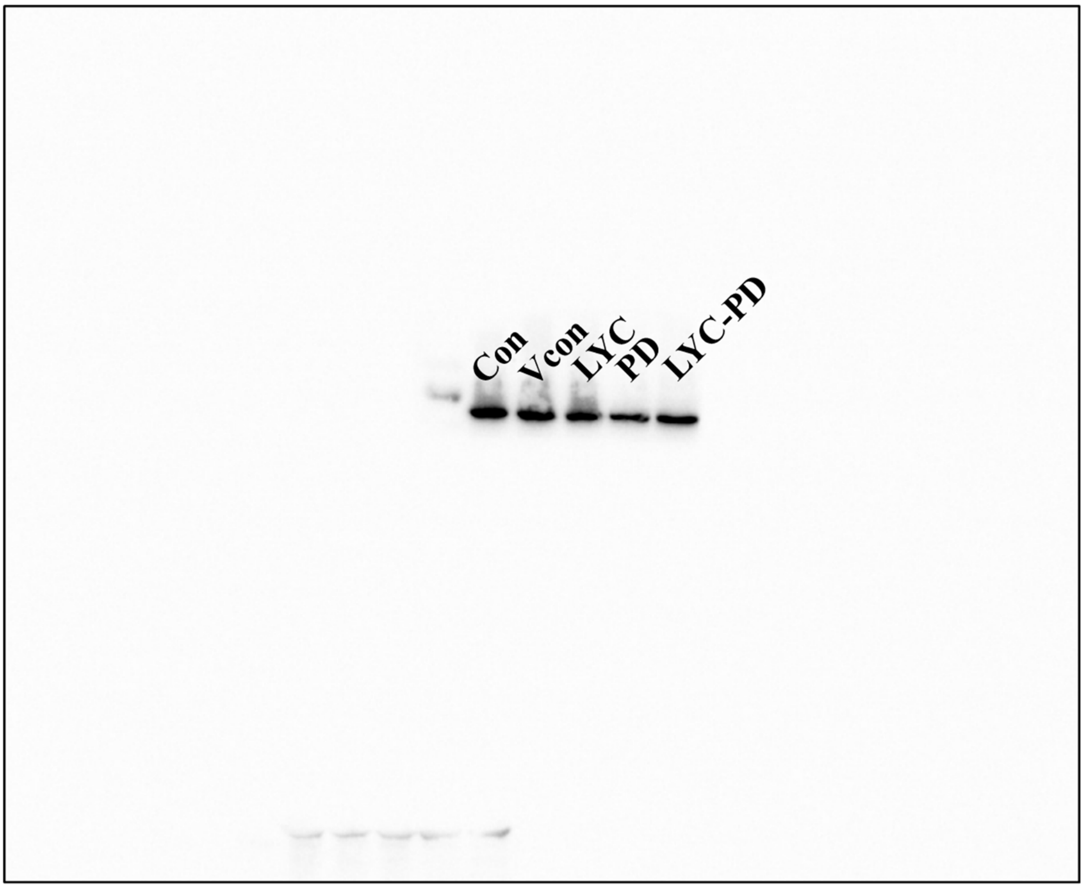

Con
Vcon
LYC
PD
LYC-PD

WB_Rep2_β-actin

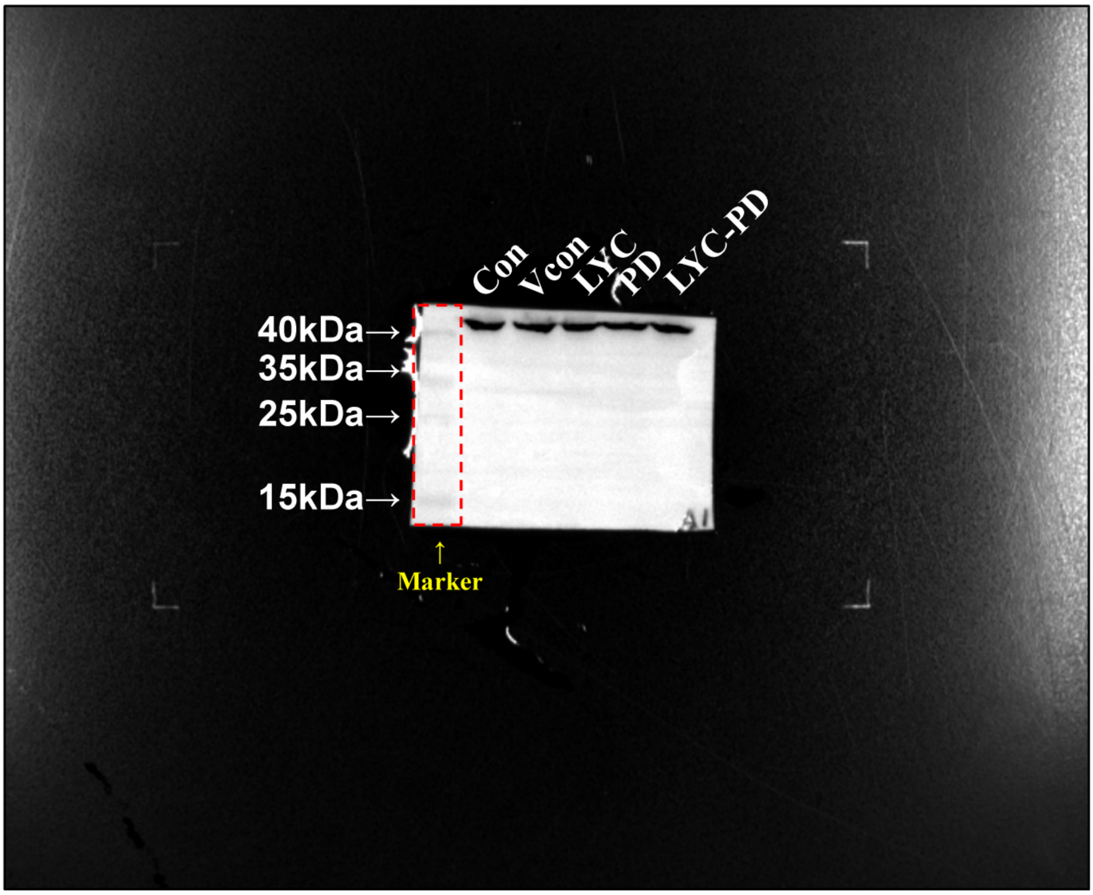

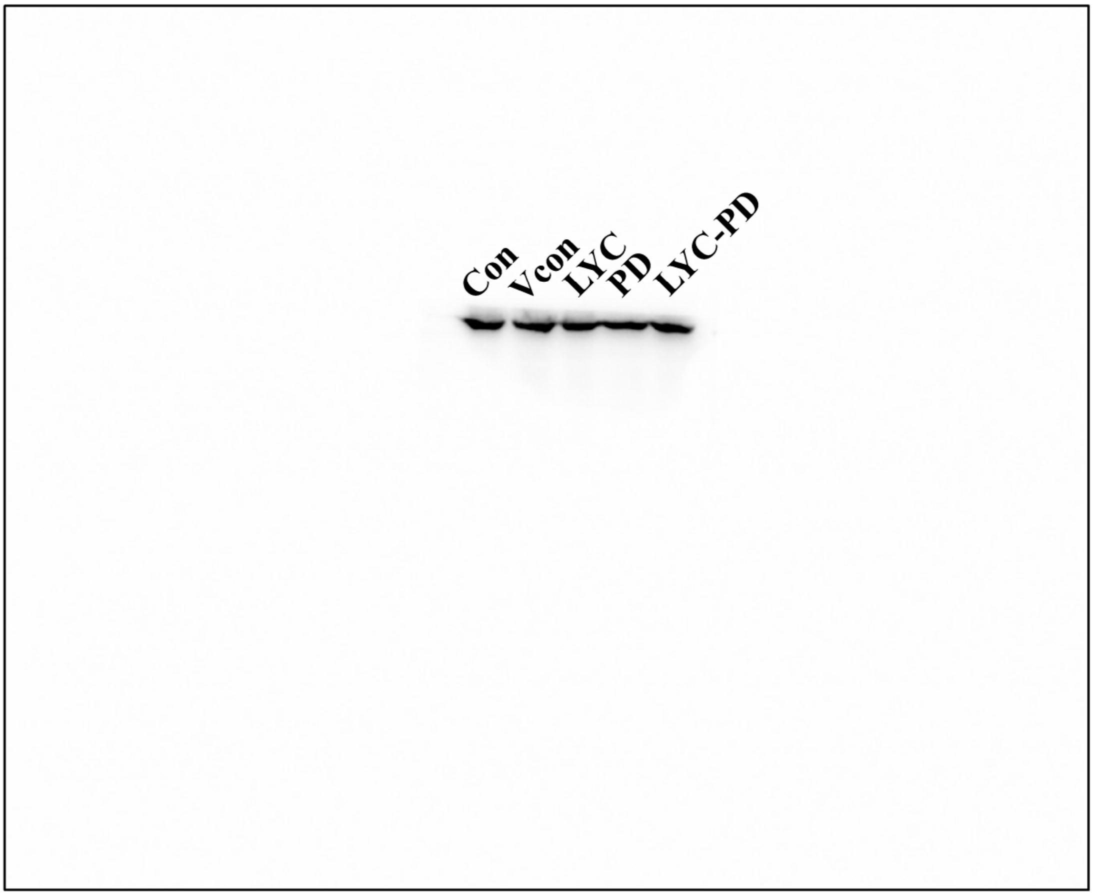

WB_Rep2_TH

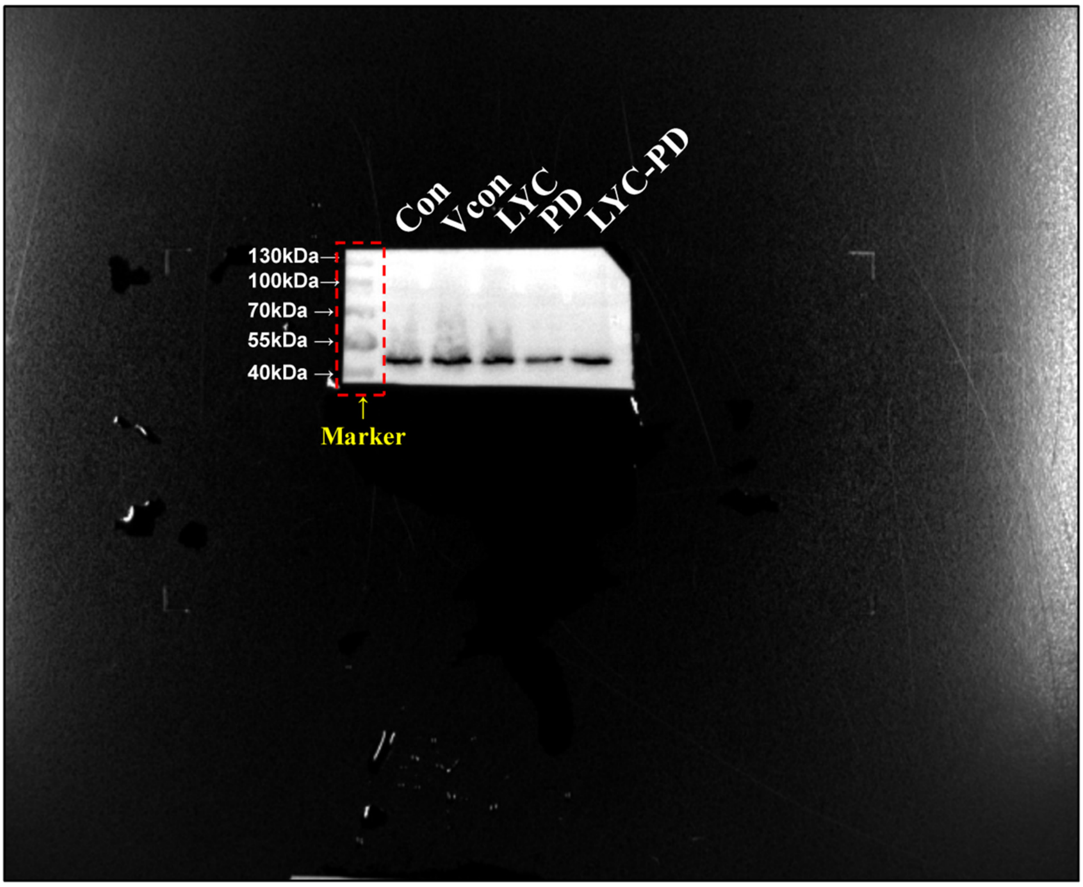

Con
Vcon
LYC
PD
LYC-PD
130kDa
100kDa
70kDa
55kDa
40kDa
Marker

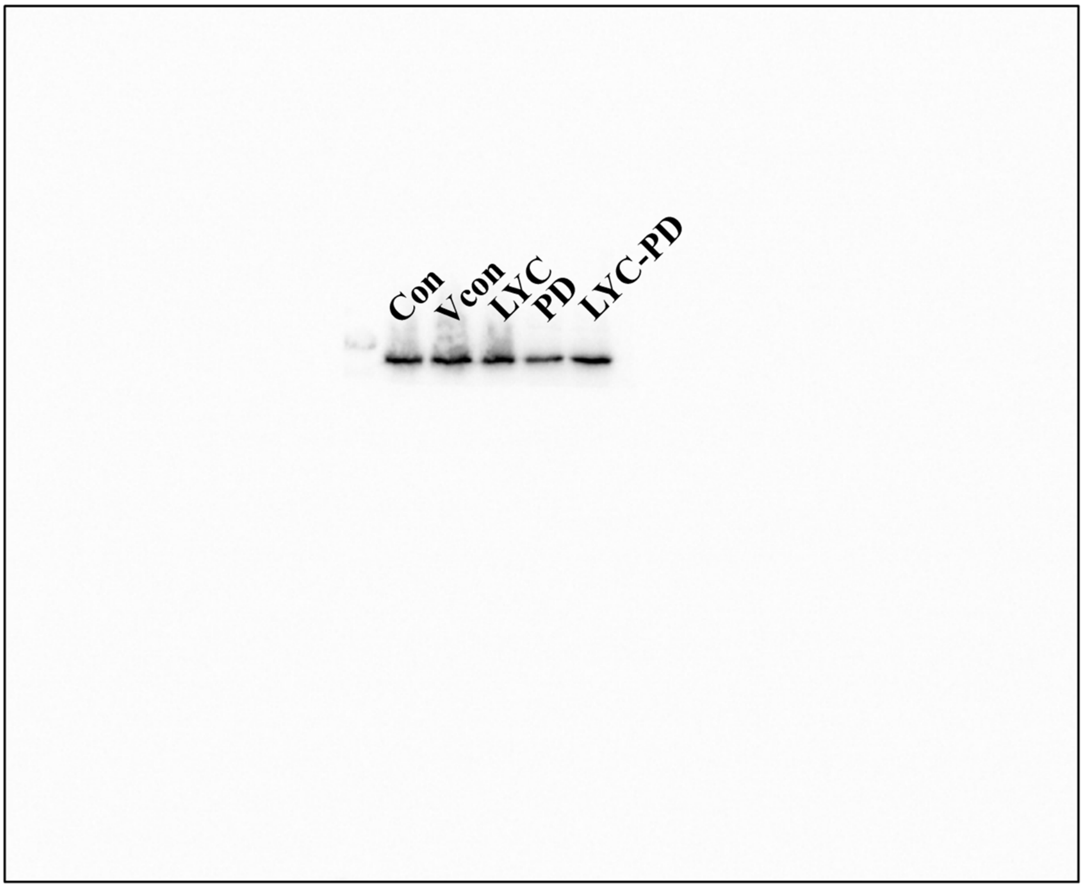

Con
Vcon
LYC
PD
LYC-PD

WB_Rep3_β-actin

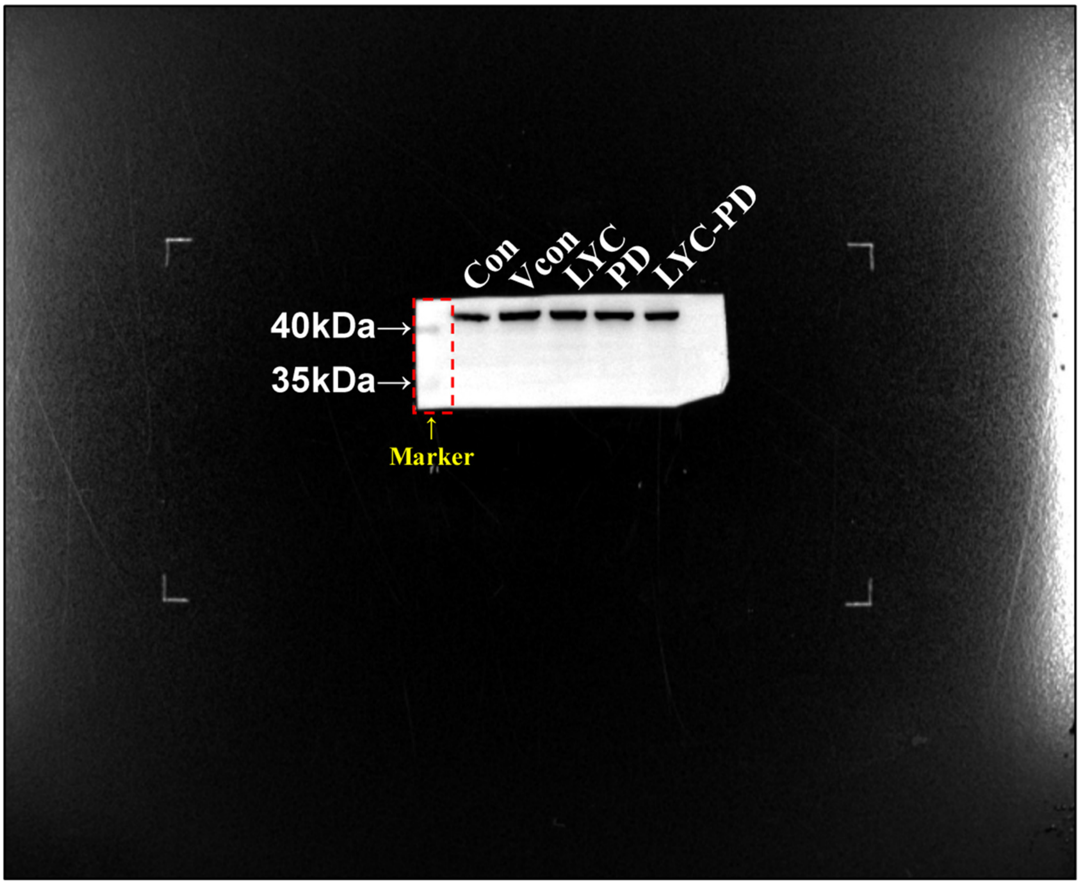

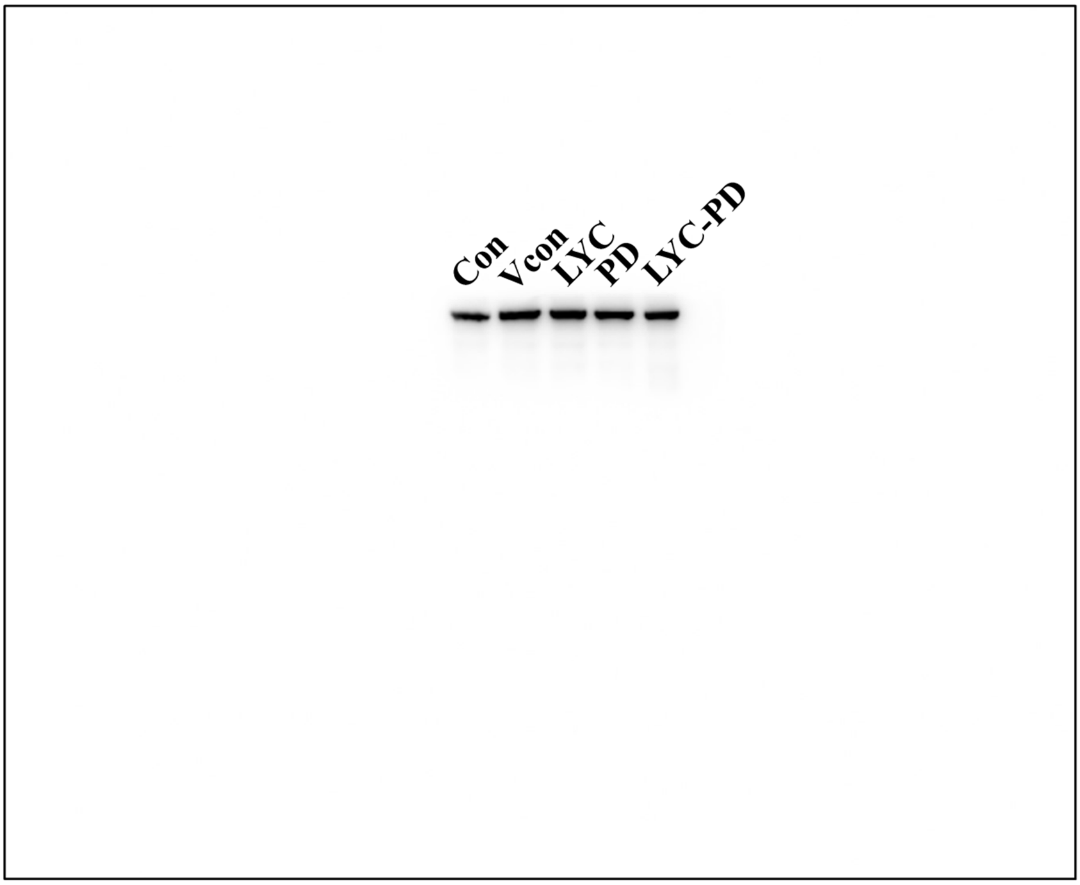

WB_Rep3_TH

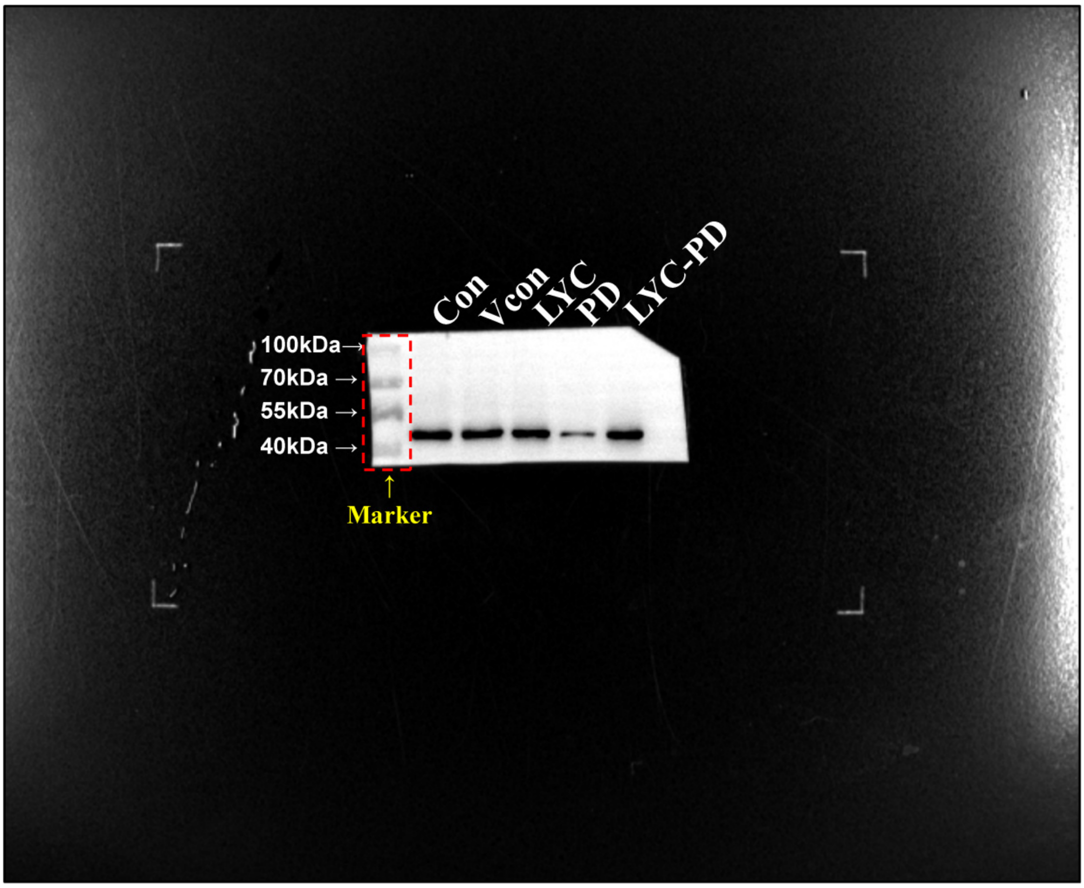
Con
Vcon
LYC
PD
LYC-PD
100kDa→
70kDa →
55kDa →
40kDa →
↑
Marker

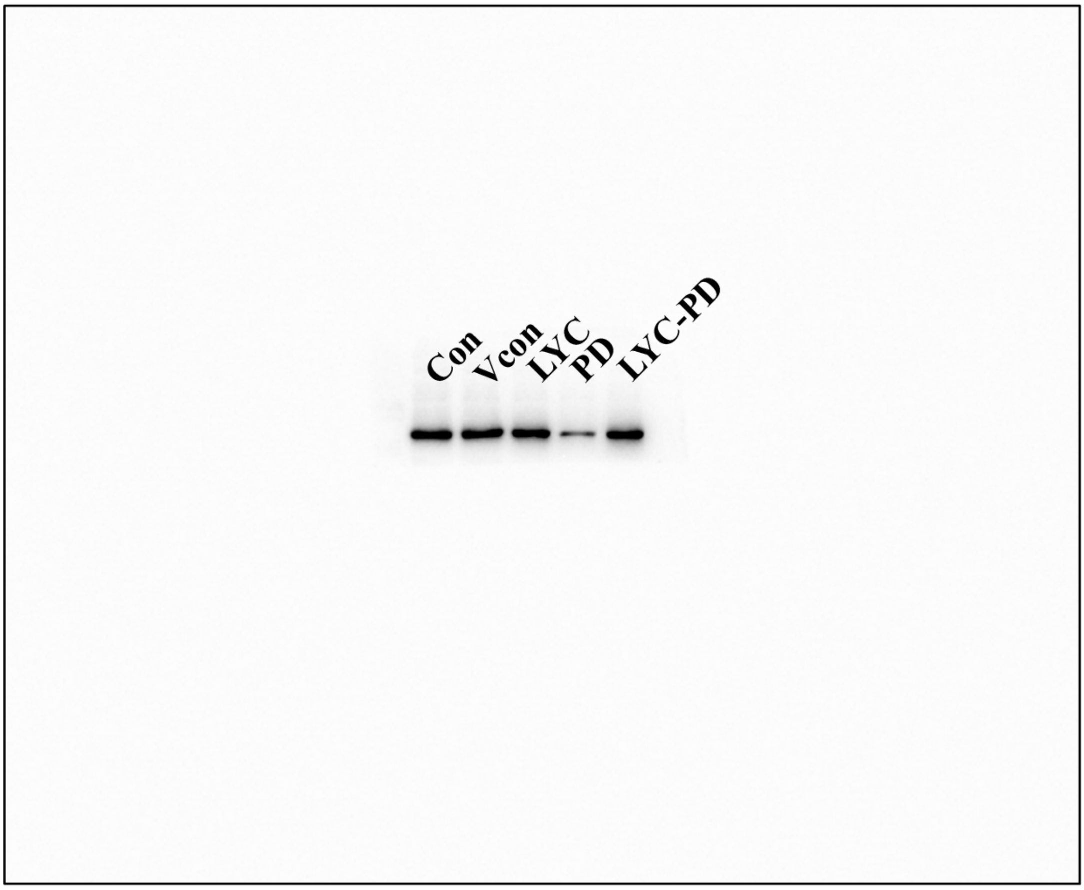
Con
Vcon
LYC
PD
LYC-PD

Supplementary Figure S2: Full-length Western blot image showing α-SYN protein expression in midbrain tissues.

Western blot analysis of α-SYN (19 kDa) and β-actin (42 kDa) proteins was performed on mouse midbrain tissues with two independent biological replicates. For each panel, the upper part shows the original images of transferred membranes, and the lower part presents images captured at different exposure times. The protein bands of β-actin and α-SYN from two independent replicates are arranged sequentially from top to bottom. The densitometric quantification results are consistent with those presented in Figure 8B of the main text.

WB_Rep1_β-actin and WB_Rep2_β-actin

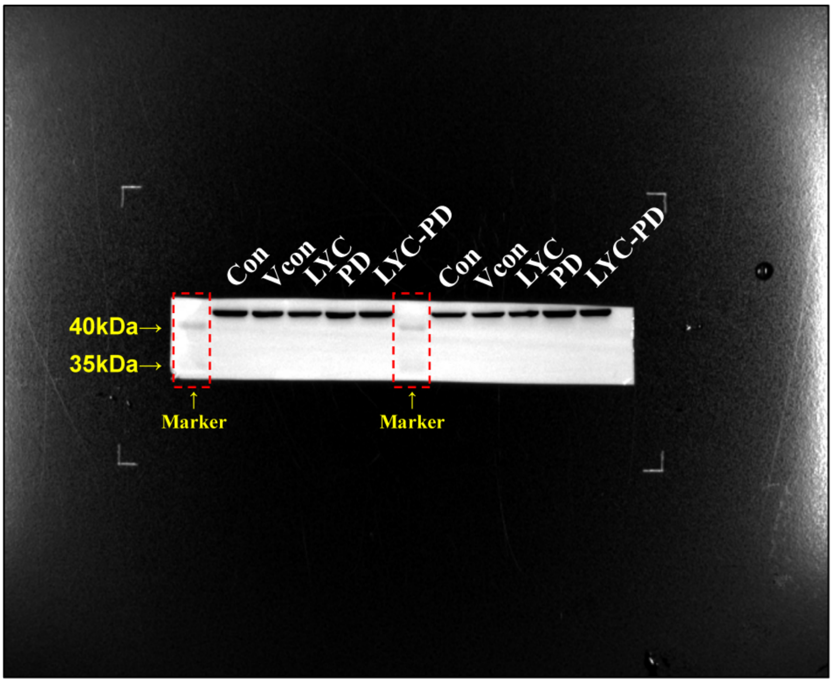

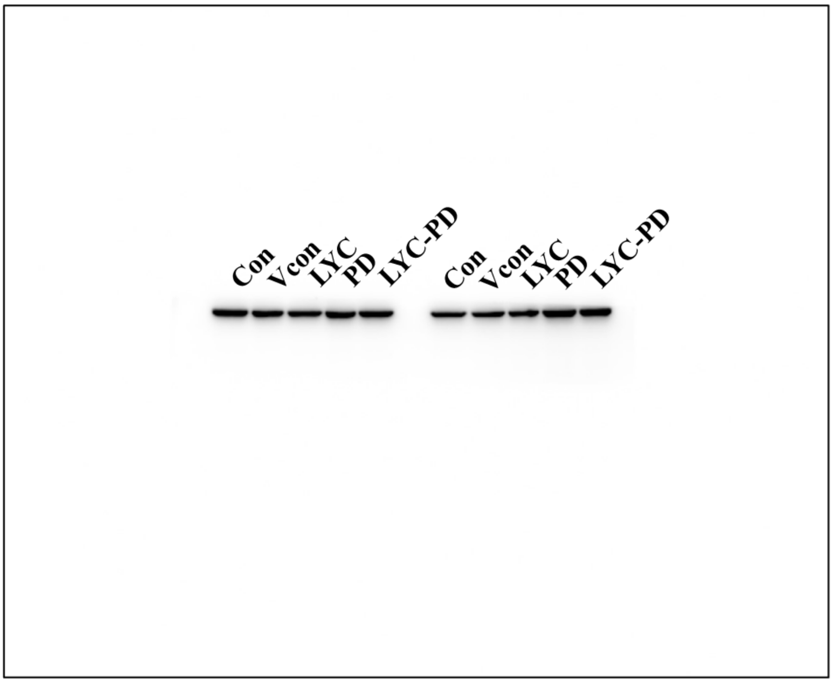

WB_Rep1_α-SYN and WB_Rep2_α-SYN

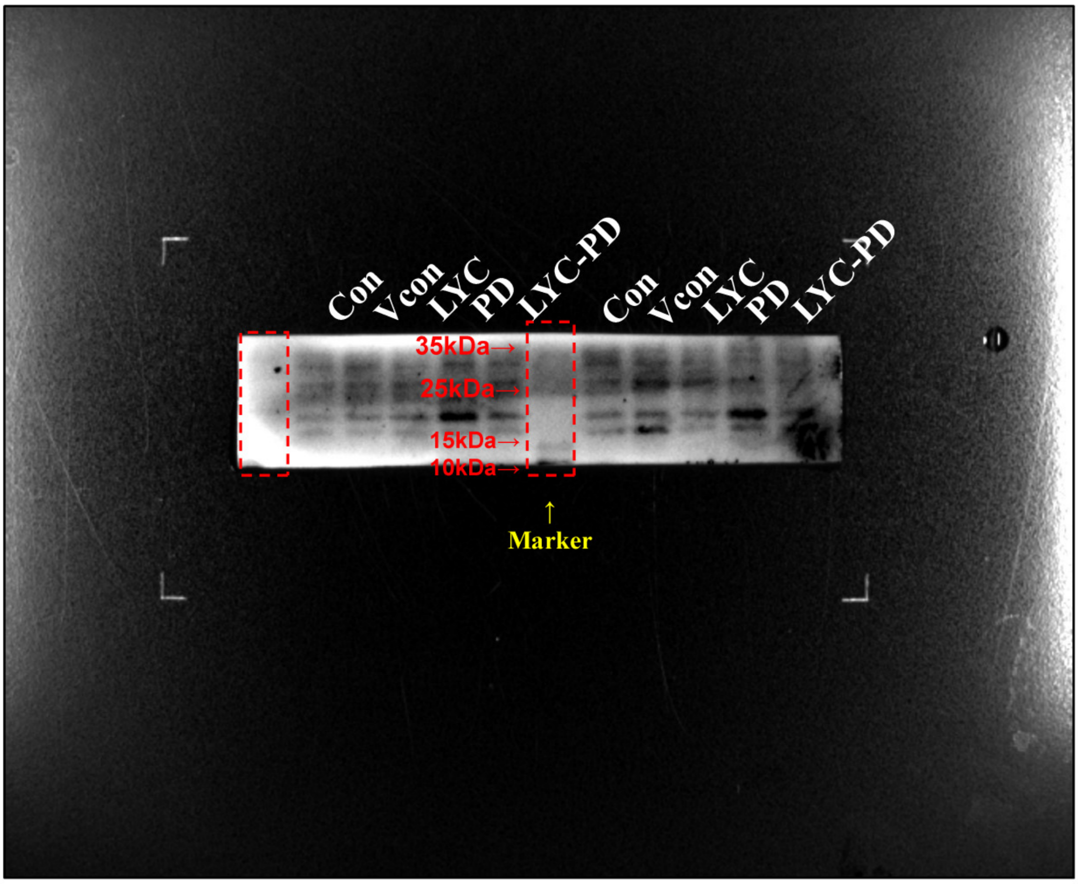

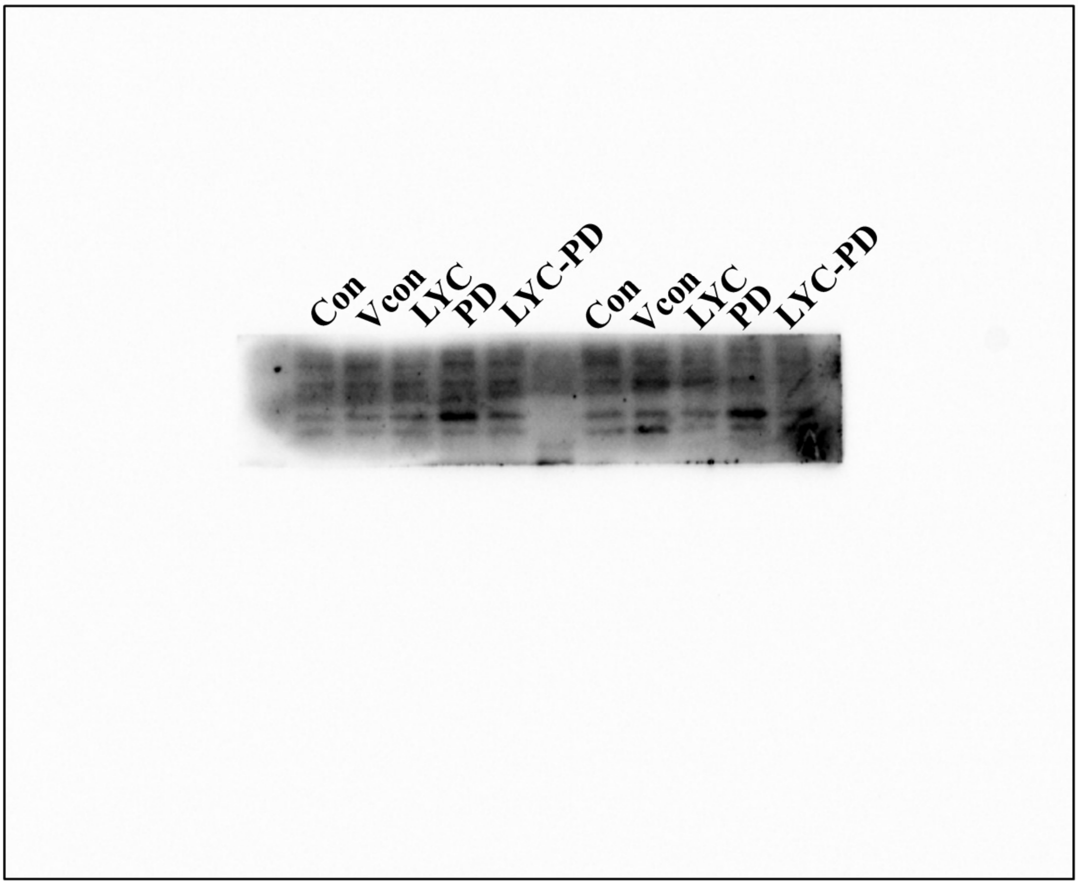

Supplementary Figure S3: Full-length Western blot image showing DAT/SLC6A3 protein expression in midbrain tissues.

Western blot analysis of DAT/SLC6A3 (68 kDa) and β-actin (42 kDa) proteins was performed on mouse midbrain tissues with two independent biological replicates. For each panel, the upper part shows the original images of transferred membranes, and the lower part presents images captured at different exposure times. The protein bands of β-actin and DAT/SLC6A3 from two independent replicates are arranged sequentially from top to bottom. The densitometric quantification results are consistent with those presented in Figure 8C of the main text.

WB_Rep1_β-actin and WB_Rep2_β-actin

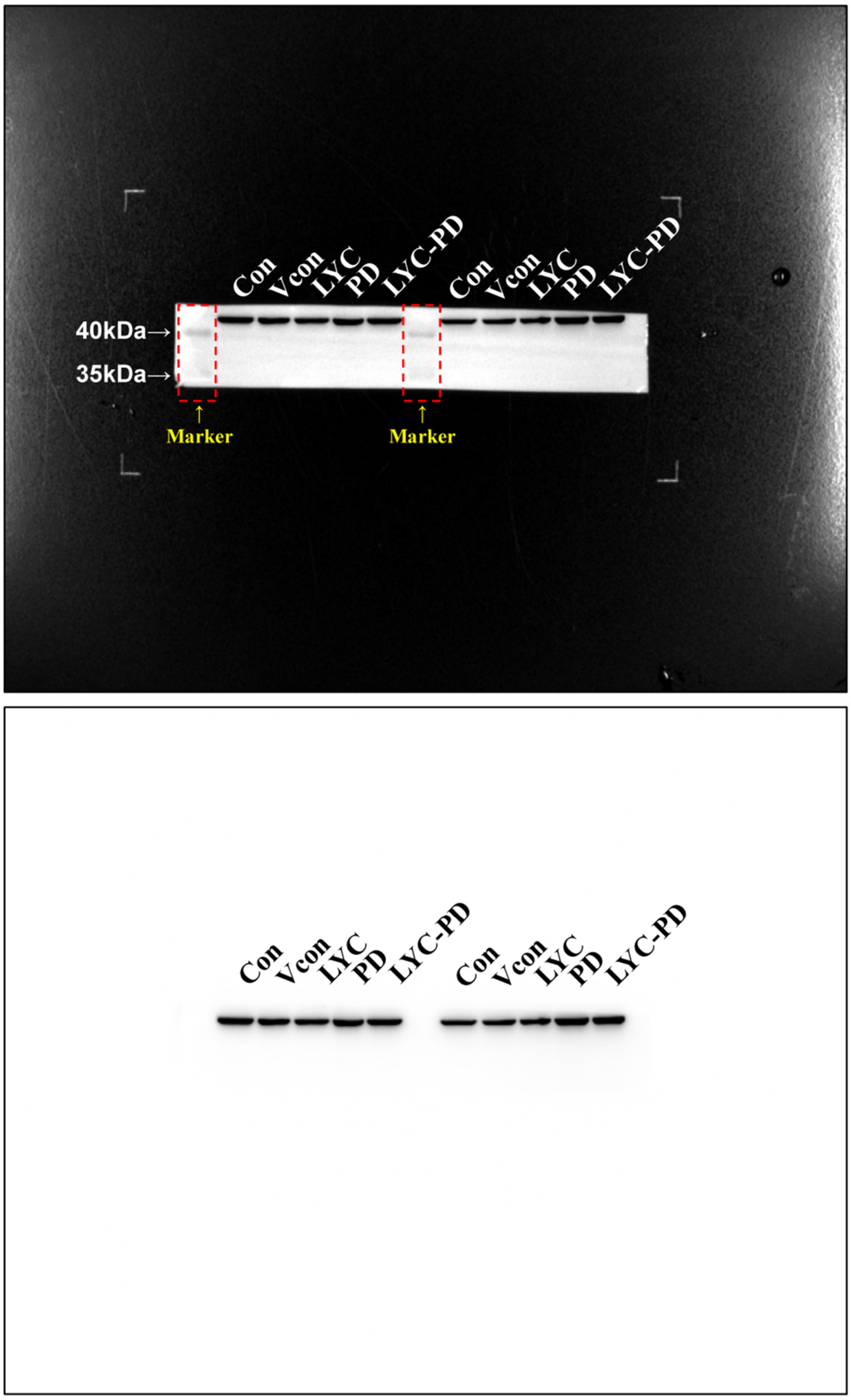

WB_Rep1_DAT/SLC6A3 and WB_Rep2_DAT/SLC6A3

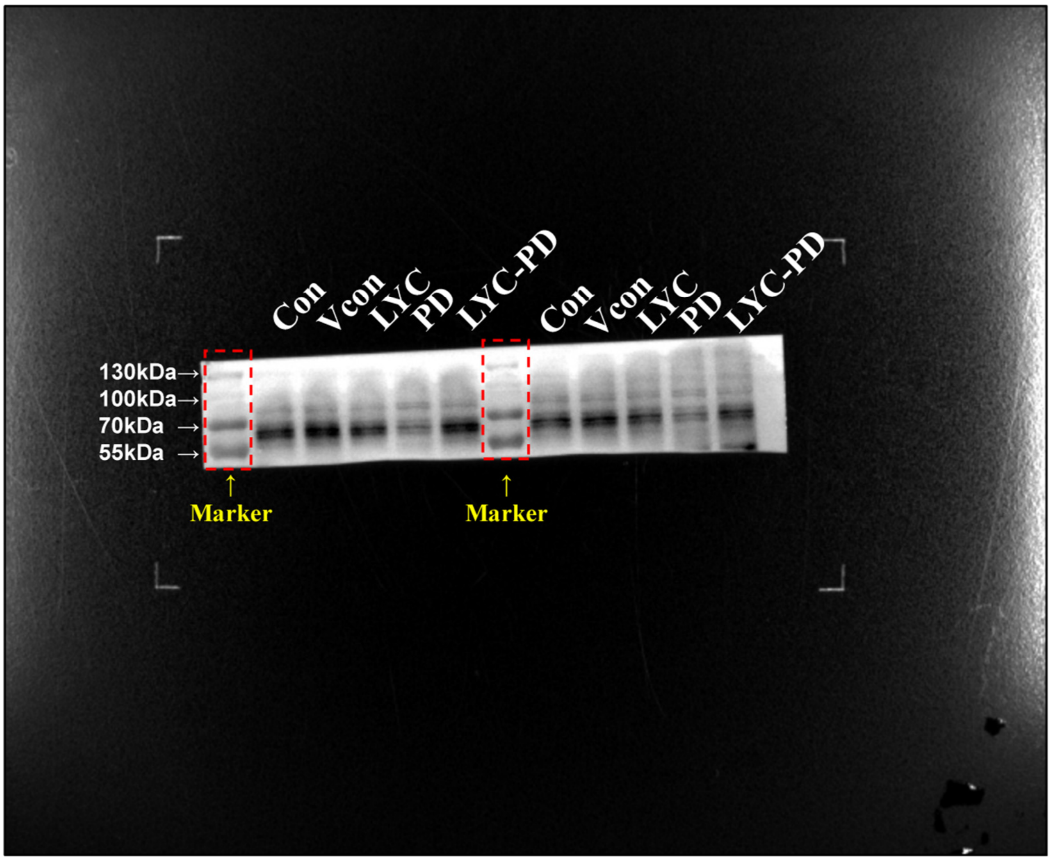

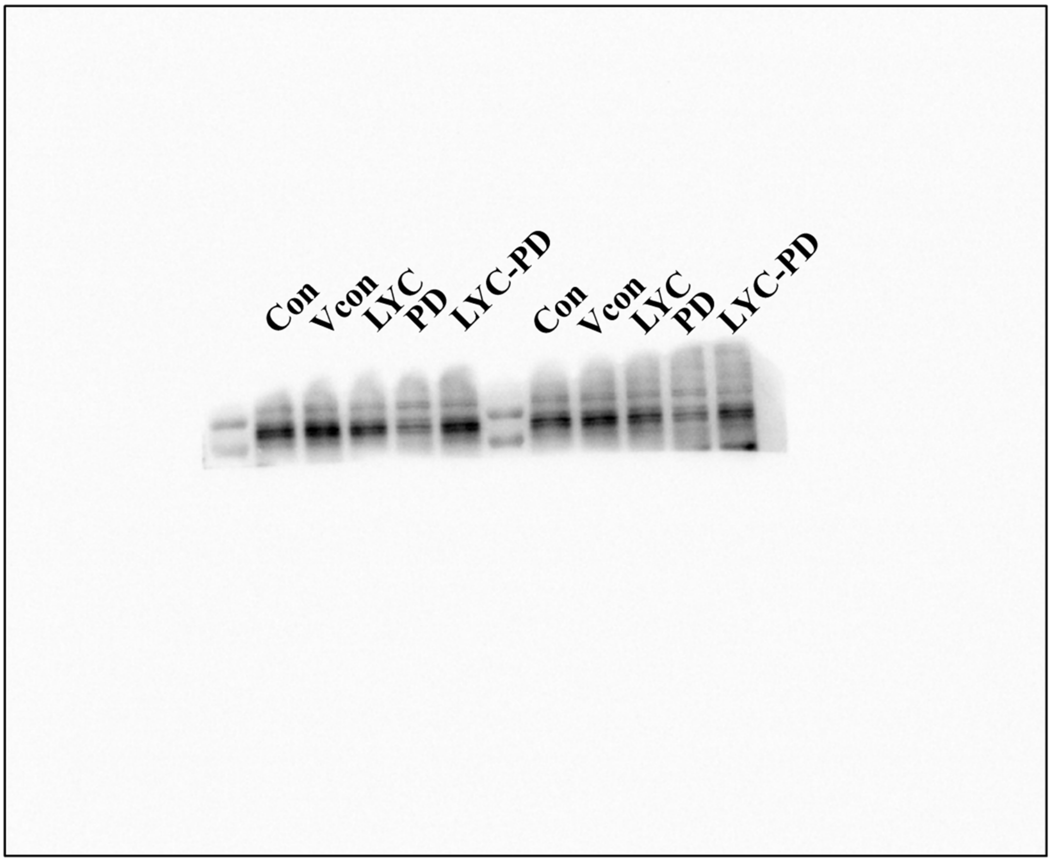

Supplementary Figure S4: Schematic layout of the 15-well SDS-PAGE gel

SDS-PAGE was performed using a 15-well comb. Lanes 1, 2 and 15 were loaded only with 1× protein loading buffer for edge protection to minimize electrophoresis edge effects. Lanes 3 and 9 were loaded with PageRuler Prestained Protein Ladder (Thermo Fisher Scientific, Cat. No. 26616). The molecular weights of ladder bands from top to bottom are 180 kDa, 130 kDa, 100 kDa, 70 kDa, 55 kDa, 40 kDa, 35 kDa, 25 kDa, 15 kDa and 10 kDa. Lanes 4, 5, 6, 7, 8 contained samples from the first batch of experimental groups, and Lanes 10, 11, 12, 13, 14 contained samples from the second batch of experimental groups. The theoretical migration positions of TH, β-actin and α-synuclein on the gel are marked in this diagram. After electrotransfer, the intact membrane was physically cut between Lane 8 and Lane 9 into two strips for separate antibody incubation No cropping or artificial post-processing was performed on all original files.

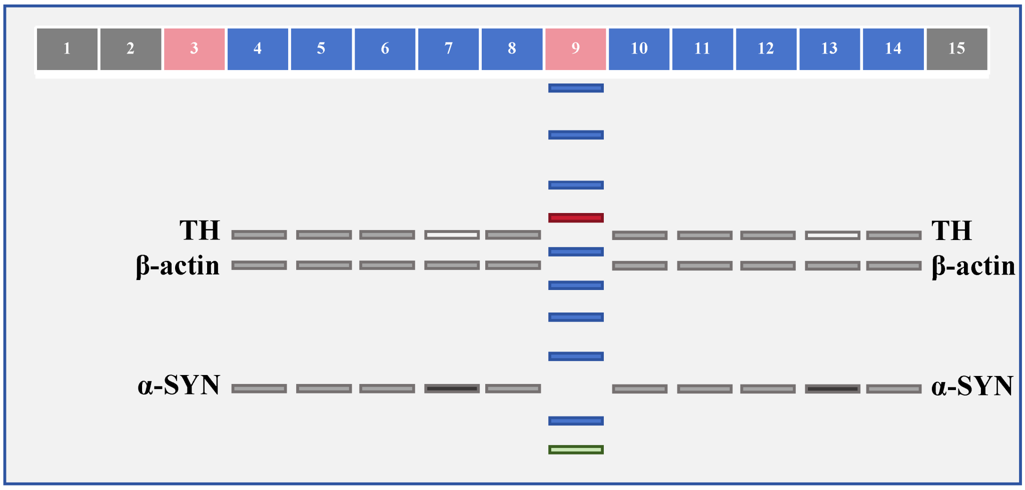

lane 1: 1× loading buffer
lane 2: 1×loading buffer
lane 3: Protein Ladder
lane 4-land 8: Con、Vcon、LYC、PD and LYC-PD group
lane 9: Protein Ladder
lane 10-land 14: Con、Vcon、LYC、PD、LYC-PD group
lane 15: 1×loading buffer

Supplementary Figure S5: Surface plasmon resonance detection of global dynamics fitting curve and repeatability of LYC-SLC6A3

(a) The global 1:1 Langmuir kinetic fitting curves for the binding of lycopene to the SLC6A3 protein at six concentration gradients are shown. Experiments were conducted with lycopene concentrations set at 0.625, 1.25, 2.5, 5, 10, and 20 μM. The entire molecular interaction process was recorded and divided into three phases: the association phase (0–120 s), the equilibrium plateau phase (120–240 s), and the dissociation phase (240–400 s). The observed response signals (RU) at different concentrations aligned well with the global fitting standard curves, confirming that the interaction conforms to a 1:1 single-site binding model.

(b) The sensorgrams for the binding of 10 μM lycopene from three independent replicates are presented. The association, equilibrium, and dissociation trajectories of the three parallel tests largely overlapped, demonstrating high stability and excellent reproducibility of the SPR detection system.

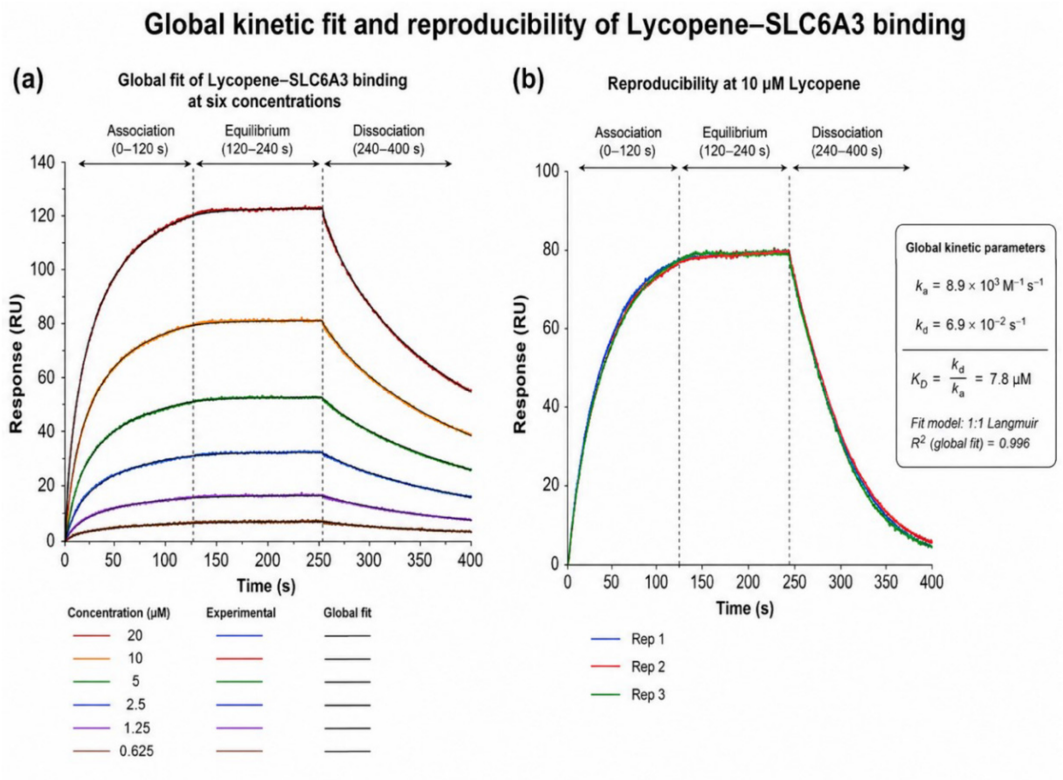

Supplementary Table S2: Statistical results of all features/metabolites in untargeted metabolome differential analysis.

Index	Compounds	P-value	FDR
MW0124741	Maltol	0.002914386	0.112422473
MW0123478	Cyromazine	0.013431896	0.199484821
MEDN1024	Piperidine acid	0.042049139	0.324968356
MW0108967	O-Acetyl-L-homoserine	0.001024539	0.085183134
MW0108676	Guanidinosuccinic acid	0.009599509	0.184562636
MW0001619	1-Hydroxypyrene	0.000753256	0.077947398
MEDP0322	L-Homoserine	0.00683349	0.16135485
MEDL01891	3-Hydroxycinnamic acid	0.00077766	0.077947398
MW0115265	Sedoheptulose 7-phosphate	0.001710663	0.091720393
MEDN0048	L-Homocystine	0.002888802	0.112422473
MEDP1785	Methyl vanillate	0.017934483	0.231778148
MEDP1589	Theonylleucine	0.047465984	0.339562291
MW0109155	Phe-Asn	0.008103976	0.173383303
ZINC895703	D-Homocysteine	0.00693105	0.16135485
MEDN0054	N-Acetylcysteine	0.03585333	0.307306191
MEDP0362	Neopterin	0.003073138	0.112422473
MW0011902	1,2-Dioleoyl-sn-Glycero-3-Phosphocholine	0.000261894	0.050229258
MW0005010	4-Formyl-2-hydroxybenzoic acid	0.04764763	0.339562291
MW0126135	Picolinamide	0.046120062	0.33552345
MW0141122	Dioleoylphosphatidylcholine	0.002228154	0.094764379
MW0124161	Formononetin	0.003856822	0.124703917
MW0126223	Praziquantel	0.034769352	0.307306191
MW0012084	11beta-PGE2	0.0473562	0.339562291
MW0168069	L-galactopyranose	0.001437217	0.086032808
MW0103576	Glycinamide ribonucleotide	0.006919507	0.16135485
MW0160175	[1,1'-Biphenyl]-2,2',3,3'-tetrol	0.004839796	0.136342195
MW0161563	1H-Indole-3-acetonitrile, N-oxide	0.004386177	0.136342195
MW0142223	2-Amino-4-oxopentanoate	0.03270151	0.303330525
MW0122775	5-Hydroxy-2-methyl-4H-pyran-4-one	0.010588385	0.184562636
MW0002160	Biphenyl-2,3-diol	0.02599368	0.273403408
MEDN0560	Marmesin	0.033095765	0.303330525
MW0109750	S-Phenyl-N-acetylcysteine	0.030840082	0.297352355
MW0139590	Kavain	0.017807368	0.231778148
MW0126737	Tetrahydropapaveroline	0.001888125	0.092124799
MW0006742	Desmethylsildenafil	0.011099128	0.184562636

MW0157398	Terpendole E	0.003155041	0.112422473
MW0004669	Aminosalicylic Acid	0.000601748	0.075046622
MW0012825	1-O-Hexadecyl-sn-glycero-3-phosphocholine	0.004630117	0.136342195
MW0141883	2-(Acetamidomethylidene)succinic acid	0.030871796	0.297352355
MW0135191	6',7'-Dihydroxybergamottin	0.017650459	0.231778148
MEDL02772	D-Erythrose 4-phosphate	0.016637719	0.223457358
MW0000801	(Z)-N-[(2R)-1-hydroxy-3-(4-hydroxyphenyl)propan-2-yl]octadec-9-enamide	0.00362779	0.121810024
MEDP1230	Glycidyl oleate	0.042063487	0.324968356
MW0008524	Thionalide	0.04513468	0.331803452
MW0003799	3-Amino-2-naphthoic acid	0.018845454	0.238435959
MW0006361	Binapacryl	0.018053739	0.231778148
MW0004825	4-Chloro-3,5-dimethoxybenzyl alcohol	0.021936779	0.253006983
MW0055335	N-octanoylsphingosine 1-phosphate	0.030082053	0.2935035
MW0125561	N-[3-(1H-imidazol-4-yl)propyl]-N'-methylthiourea	0.034713078	0.307306191
MW0169318	Isofraxidin	0.023718217	0.260452875
MW0002391	2',6'-Dihydroxy-4'-methoxyacetophenone	0.022881341	0.25511303
MW0139685	Scutellarein	0.010143037	0.184562636
MW0053756	Polyoxyethylene 40 monostearate	0.042292361	0.325297188
MW0103994	L-2-Hydroxyglutaric acid	0.044107753	0.331803452
MW0143074	Nonaprenyl-4-hydroxybenzoate	0.043284973	0.331471769
FDATN00665	Diacerein	0.005028943	0.136342195
MW0137464	Axillarin	0.007061094	0.162219339
MW0127450	Diisopropyl sulfide	0.005555591	0.142647981
MW0158014	Treosulphan	0.00189948	0.092124799
MW0000285	Homotropine	0.000805735	0.077947398
MW0115365	Tiaprofenic acid	0.009203174	0.184562636
MW0015341	9S,11R-dihydroxy-15-oxo-5Z,13E-prostadienoic acid-cyclo[8S,12R]	0.020501298	0.24909545
MEDP0060	Methionine sulfoxide	0.017991497	0.231778148
MW0156992	Serylarginine	0.002185682	0.094764379
MW0057016	1-Oleoyl-2-palmitoyl-sn-glycero-3-phosphocholine	0.021592626	0.253006983
MW0011795	1-(1Z-octadecenyl)-sn-glyce	0.024846926	0.267822995

	ro-3-phosphocholine		
MW0054541	LPC(20:2/0:0)	0.02308583	0.25511303
MW0153840	2-Methylene-3-methylsuccinic acid	0.04899087	0.342152235
MW0012818	Arachidonoyl PAF C-16	0.01300053	0.199484821
MW0114351	Psicose	0.035583935	0.307306191
MW0008848	(R)-Naproxen	0.011788745	0.190584713
MW0109704	2-[(2-Amino-3-hydroxypropanoyl)amino]-3-sulfanylpropanoic acid	0.00507574	0.136342195
MW0169694	Proadifen	0.027247296	0.281501646
MW0150088	Glu-Met-Gln	0.020686621	0.24909545
MW0157814	Thr-Thr-Gly	0.003105148	0.112422473
MW0120902	5-(N,N-Hexamethylene)amiloride	0.001167389	0.086032808
MW0123346	cis,trans-5'-Hydroxythalidomide	0.00582008	0.147273328
MW0154083	N(alpha)-gamma-L-Glutamylhistamine	0.031506406	0.297352355
MW0013614	1-Hexadecanoyl-2-(9Z-octadecenoyl)-sn-glycero-3-phosphoserine	0.04093741	0.323424065
MEDP1449	MG(0:0/22:6/0:0)	0.027562253	0.283080549
FDATN00641	Chromocarb	0.031053038	0.297352355
MW0012247	12-OxoETE	4.60211E-05	0.027140944
MW0003649	3,4-Dimethylbenzoic acid	0.048279707	0.339904714
MW0142177	Nordeoxycholic acid	0.033611739	0.305656749
MEDN0751	(±)12-HETE	0.011989561	0.192052962
MW0117082	Indole-3-propionic acid	0.02019948	0.24909545
MW0009246	O-Desmethylnaproxen	0.000955712	0.083433633
MW0122844	Ammeline	0.035117445	0.307306191
MEDN1421	(±)8-HETE	0.021537523	0.253006983
MW0108240	Methoxyacetic acid	0.013458918	0.199484821
MW0103706	Uridine-5'-diphosphate	0.030089992	0.2935035
MEDN1375	3-Phenylbutyric acid	0.048058175	0.339714876
MEDL02553	Piperine	0.011258365	0.185444386
MW0055441	Caprylic acid	0.003108469	0.112422473
ZINC1562127	10-Hydroxy-2-decenoic acid	0.022315622	0.253006983
MW0054385	Lipoxin A4	0.004723492	0.136342195
MEDN0622	Porphobilinogen	0.002159621	0.094764379
MW0002614	Tyrphostin A51	0.00999263	0.184562636
MEDL01839	8-Methyl-6-nonenoic acid	0.032356306	0.303330525
MW0104774	2-Hydroxyethanesulfonic	0.035149554	0.307306191

	acid		
MEDN0682	Octadecanamide	0.037038091	0.311015637
MEDN0684	Methyl beta-D-galactopyranoside	0.000317603	0.05041219
MW0131370	Desaminotyrosine	0.001996779	0.094226395
MW0000921	[2-(Dimethoxymethyl)-1-heptenyl]benzene	0.040765552	0.323424065
MW0002412	2,6-Di-tert-butylhydroquinone	0.016609636	0.223457358
MEDP1130	Eicosa-8,11,14-trien-5-ynoic acid	0.007527568	0.170690043
MW0169291	Idebenone	0.017220779	0.229522744
MEDN1485	Glycerophospho-N-Arachidonoyl Ethanolamine	0.008859272	0.180086789
MW0054909	Methyl hexadecanoic acid	0.000435061	0.063301331
MW0115704	Perfluorooctanesulfonic acid	0.001766229	0.091720393
MW0161153	(2S)-2-azaniumylpropanoate	0.013529243	0.199484821
MW0052794	Behenic acid	0.009812621	0.184562636
MW0058256	PE(18:1(9Z)/18:2(9Z,12Z))	0.000746377	0.077947398
MW0113880	Allose	0.013694066	0.199484821
MW0168835	beta-D-Galactose	0.028187007	0.285212259
MW0014523	4-Methoxy-3-geranylgeranyl-1,2-dihydroxybenzene	0.044621212	0.331803452
MW0011918	1,2-Dipalmitoyl-sn-glycero-3-phosphoethanolamine	0.02223961	0.253006983
MW0161223	1-octadecanoyl-2-(9Z-octadecenoyl)-sn-glycero-3-phosphoethanolamine	0.000226438	0.049420099
MW0194046	1,2-Dimyristoyl-sn-glycero-3-phosphocholine	0.044020005	0.331803452
ZINC5849312	8-Desoxygartanin	0.038719649	0.312983831
MW0104010	(S)-3-Hydroxyisobutyric acid	0.01100664	0.184562636
MW0142221	(S)-2-amino-4-chloro-4-pentenoic acid	0.007983113	0.173383303
MW0126018	N-[2-(5-hydroxy-1H-indol-3-yl)ethyl]hexadecanamide	0.012366676	0.194524467
MW0122783	Aloxistatin	0.045273609	0.331803452
MW0103999	2,3,4,5-Tetrahydro-2-pyridinecarboxylic acid	0.010143661	0.184562636
MW0139616	Rutamarin	0.029681822	0.292793563
MW0108755	1-Methyl-3-(1-methyl-1H-in	0.013267842	0.199484821

	dol-3-yl)-4-(pentylamino)-1H-pyrrole-2,5-dione		
MW0011987	10-Acetylpanaxytriol	0.038119901	0.311015637
MW0114405	Erythritol	0.024929547	0.267822995
MW0006997	Estragole	0.023974774	0.261624722
MW0169281	Hycanthone	0.001565502	0.088173134
MW0105413	Acetic anhydride	0.018274567	0.232900691
MW0062149	Prednisolone	0.008242162	0.173383303
MW0153846	N-Nitroso-N-methylurea	0.021415447	0.253006983
MW0137080	7-Hydroxyflavanone	0.005034946	0.136342195
MW0147860	Cucurbitacin A	0.049444183	0.343942408
MW0123530	Deisopropylhydroxyatrazine	0.031208023	0.297352355
MW0152829	Lycodine	0.032770977	0.303330525
MW0148548	Dimethyl telluride	0.037683513	0.311015637
MW0168654	7(S),17(S)-dihydroxy-8(E),10(Z),13(Z),15(E),19(Z)-Docosapentaenoic Acid	0.012295391	0.194524467
MW0120038	4,5-Dihydro-2-methyl-3-thiophenethiol	0.028878226	0.288122185
MW0157518	Carboxymethanesulfinyl-acetic acid	0.008870254	0.180086789
MW0015412	5-trans U-44069	0.001445512	0.086032808
MW0146836	Botrydial	0.045398664	0.331803452
MW0012359	(5S,6Z,8E,10E,12R)-5,12-dihydroxyicosa-6,8,10-trien-14-ynoic acid	0.014702487	0.208703592
MW0063233	Pubescenol	0.015626059	0.214827545
MW0012723	19,20-DiHDPA	0.011694717	0.190584713
MW0000472	Tropine	0.015625842	0.214827545
MW0140208	4-Carboxy-2-hydroxy-cis,cis-muconic acid	0.001198942	0.086032808
MW0012382	Isopalmitic acid	0.021943762	0.253006983
MW0152653	L-Galactonic acid	0.007840636	0.173383303
MW0001372	1,4-Dihydroxynaphthalene	0.008050697	0.173383303
MW0124469	Isoguvacine	0.001786079	0.091720393
MW0011920	PE-NMe(16:0/16:0)	0.015336794	0.214224341
MW0109253	Demanyl phosphate	0.033182205	0.303330525
MW0106047	Biuret	0.000287682	0.050229258
MW0105615	2-Aminoadipic acid	0.014514902	0.207729662
MW0062470	(2S)-2-amino-3-[[(2R)-2-[(Z)-hexadec-9-enoyl]oxy-3-[(Z)-octadec-11-enoyl]oxypropoxy]-hydroxyphosphoryl]o	0.005256633	0.139061824

	xypropanoic acid		
MW0056551	[(2R)-1-(10-methylundecanoyloxy)-3-phosphonooxypropan-2-yl] 10-methyldodecanoate	0.013450027	0.199484821
MW0063070	PS(22:6(4Z,7Z,10Z,13Z,16Z,19Z)/22:4(7Z,10Z,13Z,16Z))	0.008223269	0.173383303
MW0110954	Chlorsulfuron	0.036036453	0.307306191
MW0142116	2,6-Dioxo-6-phenylhexanoic acid	9.63877E-06	0.016829295
MW0155460	Phenanthrene-3,4-diol	0.036081197	0.307306191
MW0013328	25-Hydroxyvitamin D3-26,23-lactol	0.025156341	0.267822995
MW0157815	Thr-Thr-Gly-Leu-Ile	0.006689686	0.16135485
MW0148399	Diacetyl-10-gingerdiol	0.004120948	0.130821377
MW0049869	1-(9Z-hexadecenoyl)-2-(5Z,8Z,11Z,14Z,17Z-eicosapentaenoyl)-sn-glycerol	0.016414187	0.223457358

Supplementary Table S3: Statistical results of all detected metabolites in targeted metabolome differential analysis.

Compounds	Pvalue	FDR
Glutathione	5.079548493289338e-4	0.004825571068624871
5-Hydroxy-Tryptophan	0.07298294480406776	0.10666738086748365
Homogentisic Acid	5.959594144236001e-5	0.0011323228874048402
Gamma-Aminobutyric Acid	0.010707663295979358	0.025430700327950976
Ethanolamine	0.013339993378408266	0.027122723696947904
Arginine	9.519917555625637e-4	0.007235137342275484
Aspartic Acid	0.016523153017589987	0.03006577141855482
Betaine aldehyde chloride	0.04436188175289432	0.07329354376565149
Choline	0.012389564108122766	0.027122723696947904
Glutamine	0.0025814811633882028	0.012262035526093963
Glycine	0.0021098471909457724	0.011453456179419908
Histidine	4.9586017283487524e-5	0.0011323228874048402
Leucine	0.008990255817501082	0.022775314737669406
Epinephrine	0.008182986623662201	0.022210963692797406
Lysine	0.0061493262829491815	0.017974953750159145
Ornithine	0.004038842822326363	0.013952366113491073
Phenylalanine	0.0035652371237112675	0.013587127402725658
Sarcosine	0.0035755598428225416	0.013587127402725658
Serine	3.3134715316413004e-4	0.00419706394007898
Threonine	0.001330213377405655	0.008424684723569147
3,4-Dihydroxyphenylacetic Acid	0.07069089374650561	0.10666738086748365

Supplementary Table S4: Statistical results of all genes in transcriptome differential analysis.

geneID	gene_name	log2FoldChange	pvalue	padj
ENSMUSG00000026805	Barhl1	-1.3026	6.01502E-17	9.63787E-13
ENSMUSG00000042638	Gucy2c	3.23796	6.81275E-15	5.45803E-11
ENSMUSG00000006476	Nsmf	0.70539	4.22849E-14	1.80662E-10
ENSMUSG00000020732	Rab37	1.39798	4.51007E-14	1.80662E-10
ENSMUSG00000032548	Slco2a1	2.57623	7.38081E-13	2.36526E-09
ENSMUSG00000039087	Rreb1	1.4913	1.96213E-12	4.4913E-09
ENSMUSG00000048376	F2r	0.937	1.94518E-12	4.4913E-09
ENSMUSG00000049892	Rasd1	1.8274	9.31078E-12	1.86483E-08
ENSMUSG00000070570	Slc17a7	5.02327	1.11775E-11	1.98998E-08
ENSMUSG00000024747	Aldh1a7	4.26771	8.34795E-11	1.33759E-07
ENSMUSG00000078816	Prkcg	0.83524	9.25987E-11	1.34883E-07
ENSMUSG00000010080	Epn3	0.93555	1.50132E-10	2.00464E-07
ENSMUSG00000010175	Prox1	0.89565	2.0103E-10	2.47778E-07
ENSMUSG00000027577	Chrna4	0.6169	2.19704E-10	2.51451E-07
ENSMUSG00000048826	Dact2	-0.74574	3.2951E-10	3.51983E-07
ENSMUSG00000038530	Rgs4	0.63156	6.39348E-10	6.40267E-07
ENSMUSG00000015484	Fam163a	1.20518	8.63511E-10	8.13885E-07
ENSMUSG00000022044	Stmn4	0.4856	1.37537E-09	1.22431E-06
ENSMUSG00000037843	Vstm2l	-0.84763	1.91691E-09	1.59223E-06
ENSMUSG00000049001	Ndnf	1.22397	1.98743E-09	1.59223E-06
ENSMUSG00000034825	Nrip3	0.77281	2.34214E-09	1.78705E-06
ENSMUSG00000043448	Gjc2	0.92951	3.68891E-09	2.6867E-06
ENSMUSG00000051111	Sv2c	1.1367	3.93984E-09	2.7447E-06
ENSMUSG00000068859	Sp9	2.57129	7.53464E-09	5.03032E-06
ENSMUSG00000038936	Sccpdh	0.60894	7.93562E-09	5.0861E-06
ENSMUSG00000038173	Enpp6	1.11288	9.91885E-09	6.11268E-06
ENSMUSG00000009216	Fam163b	-0.86353	1.27881E-08	7.58904E-06
ENSMUSG00000022696	Sidt1	-0.64858	1.51065E-08	8.64467E-06
ENSMUSG00000032554	Trf	0.80177	1.84972E-08	1.022E-05
ENSMUSG00000026335	Pam	-0.68109	1.97718E-08	1.05601E-05
ENSMUSG00000035314	Gdpd5	0.6909	2.45804E-08	1.26243E-05
ENSMUSG00000053025	Sv2b	0.9648	2.52123E-08	1.26243E-05
ENSMUSG00000050063	Klk6	1.47447	2.74874E-08	1.33464E-05
ENSMUSG00000035486	Plk5	-1.33498	2.90194E-08	1.36758E-05
ENSMUSG00000032854	Ugt8a	1.12202	3.26784E-08	1.49602E-05
ENSMUSG00000053279	Aldh1a1	1.04983	3.77375E-08	1.67963E-05
ENSMUSG00000041607	Mbp	0.74402	3.90535E-08	1.69123E-05
ENSMUSG00000000214	Th	4.23326	5.2873E-08	2.22943E-05
ENSMUSG00000025229	Pitx3	3.33002	6.70818E-08	2.75603E-05
ENSMUSG00000013523	Bcas1	0.61408	7.01962E-08	2.77946E-05
ENSMUSG00000047797	Gjb1	0.86335	7.11215E-08	2.77946E-05
ENSMUSG00000035277	Arx	4.16819	7.84255E-08	2.99193E-05

ENSMUSG00000038065	Mturn	0.45507	8.8069E-08	3.2817E-05
ENSMUSG00000063531	Sema3e	1.21205	1.00899E-07	3.67432E-05
ENSMUSG00000035168	Tanc1	0.93025	1.13404E-07	4.03794E-05
ENSMUSG00000017978	Cadps2	1.04481	1.68715E-07	5.847E-05
ENSMUSG00000032517	Mobp	0.75772	1.71509E-07	5.847E-05
ENSMUSG00000014030	Pax5	1.0486	1.99643E-07	6.42525E-05
ENSMUSG00000026259	Ngef	-0.92686	2.00501E-07	6.42525E-05
ENSMUSG00000034413	Neurl1b	1.08089	1.92841E-07	6.42525E-05
ENSMUSG00000020599	Rgs9	-0.75311	2.27999E-07	7.1632E-05
ENSMUSG00000038375	Trp53inp2	0.57148	2.42071E-07	7.45905E-05
ENSMUSG00000044252	Osbpl1a	0.49503	2.63856E-07	7.97693E-05
ENSMUSG00000047021	Cfap65	-1.144	3.12333E-07	9.26762E-05
ENSMUSG00000004151	Etv1	0.66663	3.27837E-07	9.38025E-05
ENSMUSG00000025795	Rassf3	0.85884	3.24123E-07	9.38025E-05
ENSMUSG00000027495	Fam210b	0.71743	3.66288E-07	0.000102965
ENSMUSG00000030739	Myh14	0.55063	4.01356E-07	0.000110878
ENSMUSG00000045345	9530056K15Rik	2.18604	4.58857E-07	0.000124615
ENSMUSG00000021696	Elovl7	0.75654	4.81772E-07	0.000126548
ENSMUSG00000036634	Mag	0.61239	4.74982E-07	0.000126548
ENSMUSG00000053930	Shisa6	0.71352	5.10922E-07	0.00013204
ENSMUSG00000058740	Kcnt1	-0.45191	5.28894E-07	0.000134515
ENSMUSG00000056947	Mab21l1	-1.01541	6.26804E-07	0.000156926
ENSMUSG00000026519	Tmem63a	0.68154	6.97289E-07	0.000171887
ENSMUSG00000000794	Kcnn3	0.65253	7.10027E-07	0.00017208
ENSMUSG00000032060	Cryab	0.53872	7.19549E-07	0.00017208
ENSMUSG00000020902	Ntn1	0.92188	8.15015E-07	0.000192044
ENSMUSG00000027568	Ntsr1	2.72123	9.00718E-07	0.000209162
ENSMUSG00000063659	Zbtb18	0.57768	1.04415E-06	0.000239005
ENSMUSG00000028456	Unc13b	-0.78543	1.08436E-06	0.000244714
ENSMUSG00000037606	Osbpl5	0.6945	1.15928E-06	0.000257988
ENSMUSG00000032128	Robo3	-1.66321	1.19807E-06	0.000262969
ENSMUSG00000011884	Gltp	0.61711	1.2177E-06	0.000263664
ENSMUSG00000031997	Trpc6	2.09949	1.33235E-06	0.000284644
ENSMUSG00000031425	Plp1	0.67378	1.37013E-06	0.000288864
ENSMUSG00000026170	Cyp27a1	0.7136	1.57376E-06	0.000327485
ENSMUSG00000056427	Slit3	1.24372	1.62826E-06	0.000334482
ENSMUSG00000026028	Trak2	0.59353	1.89971E-06	0.000385304
ENSMUSG00000058070	Eml1	0.49551	2.13152E-06	0.000426917
ENSMUSG00000033060	Lmo7	0.87693	2.22022E-06	0.000439192
ENSMUSG00000090061	Nwd2	0.83451	2.48901E-06	0.000486359
ENSMUSG00000062647	Rpl7a	-0.97577	2.54747E-06	0.000491785
ENSMUSG00000034796	Cpne7	2.5101	2.63441E-06	0.000502513
ENSMUSG00000033579	Fa2h	0.75238	2.76755E-06	0.000521699
ENSMUSG00000026443	Lrrn2	0.44544	3.21061E-06	0.000598181

ENSMUSG00000035594	Chrna5	2.21934	3.44573E-06	0.000634608
ENSMUSG00000030110	Ret	2.26813	3.7043E-06	0.0006669
ENSMUSG00000041957	Pkp2	0.99034	3.68432E-06	0.0006669
ENSMUSG00000021609	Slc6a3	5.22914	4.14605E-06	0.000738134
ENSMUSG00000055945	Prr18	0.60463	4.20304E-06	0.000740059
ENSMUSG00000027859	Ngf	2.93067	4.49309E-06	0.00078253
ENSMUSG00000056296	Synpr	-0.79551	4.58329E-06	0.000789657
ENSMUSG00000002055	Spag5	-0.87904	4.69966E-06	0.000795277
ENSMUSG00000045659	Plekha7	0.46955	4.71518E-06	0.000795277
ENSMUSG00000039607	Rbms3	0.56789	4.86358E-06	0.000811761
ENSMUSG00000057766	Ankrd29	0.58129	5.27265E-06	0.000870965
ENSMUSG00000026830	Ermn	0.82275	5.38225E-06	0.000879999
ENSMUSG00000110929	Gm47757	-1.8291	5.5807E-06	0.000903227
ENSMUSG00000006782	Cnp	0.5294	6.11885E-06	0.000970717
ENSMUSG00000057606	Colq	2.19169	6.1033E-06	0.000970717
ENSMUSG00000022103	Gfra2	-0.74433	6.50486E-06	0.001021837
ENSMUSG00000026826	Nr4a2	2.84423	6.63407E-06	0.001022093
ENSMUSG00000037166	Ppp1r14a	0.69846	6.587E-06	0.001022093
ENSMUSG00000105265	Sox2ot	0.66266	6.83509E-06	0.001043034
ENSMUSG00000033006	Sox10	0.51882	6.99924E-06	0.001058007
ENSMUSG00000020696	Rffl	0.623	7.23332E-06	0.001073143
ENSMUSG00000033316	Galnt9	0.39172	7.17332E-06	0.001073143
ENSMUSG00000032841	Prr5l	0.7084	7.3936E-06	0.001086859
ENSMUSG00000046743	Fat4	-0.59524	7.55657E-06	0.001100718
ENSMUSG00000006301	Tmbim1	0.51339	7.91961E-06	0.00112545
ENSMUSG00000024008	Cpne5	-1.04878	7.80223E-06	0.00112545
ENSMUSG00000061762	Tac1	-1.16445	7.93708E-06	0.00112545
ENSMUSG00000052301	Doc2a	-0.85583	8.243E-06	0.001158576
ENSMUSG00000039103	Nexn	1.41237	9.00148E-06	0.00125418
ENSMUSG00000076439	Mog	0.63704	9.09746E-06	0.001256625
ENSMUSG00000032259	Drd2	0.7993	9.48888E-06	0.001288478
ENSMUSG00000051065	Mb21d2	0.62286	9.41996E-06	0.001288478
ENSMUSG00000020099	Unc5b	0.47843	9.68179E-06	0.001291471
ENSMUSG00000041235	Chd7	0.68754	9.70176E-06	0.001291471
ENSMUSG00000073424	Cyp4f15	-0.81503	9.75273E-06	0.001291471
ENSMUSG00000026435	Slc45a3	1.40525	1.02766E-05	0.001349682
ENSMUSG00000003518	Dusp3	0.44264	1.05413E-05	0.001357518
ENSMUSG00000006930	Hap1	-0.43061	1.05904E-05	0.001357518
ENSMUSG00000030701	Plekhb1	0.51624	1.04957E-05	0.001357518
ENSMUSG00000062372	Otof	-0.76291	1.0713E-05	0.001362332
ENSMUSG00000023017	Asic1	-0.58332	1.11746E-05	0.001409843
ENSMUSG00000019986	Ahi1	-0.48527	1.1406E-05	0.00141673
ENSMUSG00000071203	Naip5	1.35649	1.13785E-05	0.00141673
ENSMUSG00000062380	Tubb3	0.48831	1.17397E-05	0.001446964

ENSMUSG00000000078	Klf6	0.50028	1.21562E-05	0.001464497
ENSMUSG00000021684	Pde8b	-0.55519	1.20354E-05	0.001464497
ENSMUSG00000039904	Gpr37	0.51588	1.20684E-05	0.001464497
ENSMUSG00000021752	Kctd6	0.64376	1.2572E-05	0.001482724
ENSMUSG00000031775	Pllp	0.58681	1.2603E-05	0.001482724
ENSMUSG00000033615	Cplx1	0.59796	1.24868E-05	0.001482724
ENSMUSG00000038732	Mboat1	1.08159	1.26776E-05	0.001482724
ENSMUSG00000024597	Slc12a2	0.5109	1.28652E-05	0.001493742
ENSMUSG00000032303	Chrna3	-0.89233	1.29583E-05	0.001493742
ENSMUSG00000020905	Usp43	2.17296	1.30824E-05	0.001497285
ENSMUSG00000030605	Mfge8	-0.46412	1.32467E-05	0.001505337
ENSMUSG00000021143	Pacs2	0.44252	1.3442E-05	0.001516774
ENSMUSG00000048218	Amigo2	-0.78518	1.357E-05	0.001520504
ENSMUSG00000063430	Wscd2	-0.77143	1.377E-05	0.001532196
ENSMUSG00000033208	S100b	0.65496	1.3936E-05	0.001539978
ENSMUSG00000055435	Maf	-0.8056	1.42507E-05	0.001563961
ENSMUSG00000020701	Tmem132e	-0.59628	1.44847E-05	0.001578833
ENSMUSG00000021685	Otp	4.13414	1.51344E-05	0.001632289
ENSMUSG00000035783	Acta2	0.80914	1.51789E-05	0.001632289
ENSMUSG00000061451	Tmem151a	0.47475	1.5454E-05	0.001640098
ENSMUSG00000062591	Tubb4a	0.45561	1.54562E-05	0.001640098
ENSMUSG00000029122	Evc	-0.62956	1.58485E-05	0.001648966
ENSMUSG00000037295	Ldlrap1	1.34445	1.58409E-05	0.001648966
ENSMUSG00000060402	Chst8	0.56006	1.57869E-05	0.001648966
ENSMUSG00000037625	Cldn11	0.56058	1.67407E-05	0.001708515
ENSMUSG00000047507	Baiap3	-0.60306	1.67395E-05	0.001708515
ENSMUSG00000048251	Bcl11b	-0.71138	1.6544E-05	0.001708515
ENSMUSG00000075012	Fjx1	0.59934	1.72173E-05	0.001746026
ENSMUSG00000032500	Dclk3	-0.67978	1.77026E-05	0.001772807
ENSMUSG00000035441	Myo1d	0.64802	1.76066E-05	0.001772807
ENSMUSG00000022416	Cacna1i	-0.63235	1.86644E-05	0.001835158
ENSMUSG00000046167	Gldn	1.95612	1.87834E-05	0.001835158
ENSMUSG00000048100	Taf13	0.60232	1.86218E-05	0.001835158
ENSMUSG00000087658	Hotairm1	2.72508	1.87745E-05	0.001835158
ENSMUSG00000036111	Lmo1	-0.84888	1.96827E-05	0.001911366
ENSMUSG00000047228	A2ml1	1.54975	2.02046E-05	0.001938551
ENSMUSG00000100147	1700047M11Rik	0.61473	2.01803E-05	0.001938551
ENSMUSG00000056596	Trnp1	0.35688	2.04465E-05	0.001950083
ENSMUSG00000021991	Cacna2d3	0.6298	2.06062E-05	0.001953687
ENSMUSG00000015968	Cacna1d	-0.68539	2.0883E-05	0.001968284
ENSMUSG00000026655	Fam107b	0.95419	2.10229E-05	0.00196988
ENSMUSG00000029576	Radil	-0.51762	2.14461E-05	0.001997856
ENSMUSG00000028785	Hpca	-0.66354	2.21107E-05	0.002047859
ENSMUSG00000036098	Myrf	0.52501	2.43681E-05	0.002243968

ENSMUSG00000028691	Prdx1	0.44526	2.48698E-05	0.002277076
ENSMUSG00000010825	Grid2ip	1.64549	2.53483E-05	0.002307705
ENSMUSG00000045087	S1pr5	0.58438	2.59622E-05	0.002337031
ENSMUSG00000070802	Pnma8b	-0.41	2.58298E-05	0.002337031
ENSMUSG00000026879	Gsn	0.72586	2.63369E-05	0.002346633
ENSMUSG00000032418	Me1	0.40158	2.65082E-05	0.002346633
ENSMUSG00000037996	Slc24a2	0.63964	2.64201E-05	0.002346633
ENSMUSG00000063296	Tmem117	0.62379	2.68097E-05	0.002360284
ENSMUSG00000013089	Etv5	0.50325	2.69826E-05	0.00236253
ENSMUSG00000037984	Neurod6	2.17708	2.7848E-05	0.002425048
ENSMUSG00000034156	Tspoap1	-0.44394	2.93064E-05	0.002538247
ENSMUSG00000028370	Pappa	1.14051	3.14029E-05	0.002705207
ENSMUSG00000020672	Sntg2	-1.21709	3.2229E-05	0.002761522
ENSMUSG00000073680	Tmem88b	0.56013	3.29392E-05	0.002807369
ENSMUSG00000020027	Socs2	0.69333	3.40485E-05	0.002871361
ENSMUSG00000070407	Hs3st3b1	-1.61039	3.39097E-05	0.002871361
ENSMUSG00000071856	Mcc	-0.49841	3.42648E-05	0.002874474
ENSMUSG00000026834	Acvr1c	1.16886	3.49349E-05	0.002915423
ENSMUSG00000046321	Hs3st2	-0.83777	3.5176E-05	0.002920335
ENSMUSG00000027199	Gatm	0.39736	3.61372E-05	0.002977894
ENSMUSG00000031007	Atp6ap2	0.3818	3.6241E-05	0.002977894
ENSMUSG00000036545	Adamts2	1.068	3.65657E-05	0.002989243
ENSMUSG00000050854	Tmem125	0.66545	3.83019E-05	0.003115288
ENSMUSG00000026688	Mgst3	0.6332	3.93284E-05	0.003182624
ENSMUSG00000031750	Il34	-0.55258	3.96992E-05	0.003196484
ENSMUSG00000022899	Slc15a2	0.59867	4.03677E-05	0.00323406
ENSMUSG00000038668	Lpar1	0.60203	4.08124E-05	0.003253417
ENSMUSG00000019232	Etnppl	-0.63059	4.12574E-05	0.003272613
ENSMUSG00000001911	Nfix	0.64677	4.19427E-05	0.003297611
ENSMUSG00000032666	1700025G04Rik	-0.40991	4.19842E-05	0.003297611
ENSMUSG00000020758	Itgb4	0.65788	4.39253E-05	0.003423497
ENSMUSG00000055240	Zfp101	0.65826	4.40142E-05	0.003423497
ENSMUSG00000006218	Fam131c	0.76822	4.42579E-05	0.003425816
ENSMUSG00000000142	Axin2	-0.95466	4.4853E-05	0.003436502
ENSMUSG00000027375	Mal	0.50831	4.50393E-05	0.003436502
ENSMUSG00000037946	Fgd3	0.60558	4.4777E-05	0.003436502
ENSMUSG00000050447	Lypd6	-1.0399	4.58255E-05	0.003479914
ENSMUSG00000018427	Ypel2	0.47355	4.71407E-05	0.003562905
ENSMUSG00000000247	Lhx2	-0.6885	4.82812E-05	0.003565025
ENSMUSG00000024661	Fth1	0.63035	4.82533E-05	0.003565025
ENSMUSG00000040860	Crocc	-0.50436	4.81303E-05	0.003565025
ENSMUSG00000042035	Igsf3	-0.74004	4.75947E-05	0.003565025
ENSMUSG00000045136	Tubb2b	0.63046	4.76202E-05	0.003565025
ENSMUSG00000005125	Ndrg1	0.56911	4.8701E-05	0.003579523

ENSMUSG00000032268	Tmprss5	1.15569	4.97601E-05	0.003640667
ENSMUSG00000046961	Gpr156	-0.94793	5.1155E-05	0.003725713
ENSMUSG00000026976	Pax8	1.5179	5.17722E-05	0.003745884
ENSMUSG00000039714	Cplx3	-1.91815	5.18995E-05	0.003745884
ENSMUSG00000020183	Cpm	0.56375	5.26024E-05	0.003779587
ENSMUSG00000048537	Phldb1	0.42785	5.33125E-05	0.003813512
ENSMUSG00000097789	Ctxnd1	0.79643	5.45548E-05	0.003885027
ENSMUSG00000079179	Rab10os	-0.36363	5.48206E-05	0.003886683
ENSMUSG00000032355	Mlip	1.16424	5.56818E-05	0.003930348
ENSMUSG00000038526	Car14	0.75554	5.83962E-05	0.004103869
ENSMUSG00000022090	Pdlim2	0.63374	5.92659E-05	0.004146803
ENSMUSG00000053963	Stum	0.59019	5.96557E-05	0.004155927
ENSMUSG00000015714	Cers2	0.44308	6.04088E-05	0.00417211
ENSMUSG00000041309	Nkx6-2	0.68344	6.02863E-05	0.00417211
ENSMUSG00000027858	Tspan2	0.43471	6.11925E-05	0.004190117
ENSMUSG00000038775	Vill	1.88315	6.10938E-05	0.004190117
ENSMUSG00000042425	Frmpd3	-0.47679	6.16286E-05	0.004202023
ENSMUSG00000029832	Nfe2l3	0.81788	6.31818E-05	0.00428967
ENSMUSG00000112666	Gm48485	-1.17043	6.35528E-05	0.004296653
ENSMUSG00000073910	Mob3b	1.24137	6.51849E-05	0.00438848
ENSMUSG00000030730	Atp2a1	1.79896	6.70567E-05	0.004495603
ENSMUSG00000000861	Bcl11a	-0.48587	6.82862E-05	0.004518514
ENSMUSG00000022306	Zfpm2	-0.55422	6.87086E-05	0.004518514
ENSMUSG00000026255	Efhd1	0.40268	6.89002E-05	0.004518514
ENSMUSG00000032452	Clstn2	0.44918	6.78745E-05	0.004518514
ENSMUSG00000040856	Dlk1	1.83076	6.90904E-05	0.004518514
ENSMUSG00000062296	Trank1	-0.30795	6.82697E-05	0.004518514
ENSMUSG00000022548	Apod	0.67742	6.98556E-05	0.004549983
ENSMUSG00000060126	Tpt1	0.34346	7.07618E-05	0.004590352
ENSMUSG00000019873	Reep3	0.4163	7.18281E-05	0.004622094
ENSMUSG00000039257	Vstm2b	-0.54055	7.16611E-05	0.004622094
ENSMUSG00000033209	Ttc28	-0.64027	7.24283E-05	0.004642074
ENSMUSG00000026103	Gls	0.60846	7.31499E-05	0.004669643
ENSMUSG00000092627	D130058E05Rik	3.15637	7.70998E-05	0.004902264
ENSMUSG00000027030	Stk39	0.40034	7.84809E-05	0.004966182
ENSMUSG00000030306	Tmtc1	-0.43729	7.89081E-05	0.004966182
ENSMUSG00000033949	Trim36	0.58393	7.90349E-05	0.004966182
ENSMUSG00000036777	Anln	0.73135	8.0323E-05	0.005027401
ENSMUSG00000041771	Slc24a4	-0.76966	8.18322E-05	0.005101933
ENSMUSG00000032087	Dscaml1	-0.70158	8.30176E-05	0.005155778
ENSMUSG00000020411	Nipal4	0.83368	8.41301E-05	0.00518468
ENSMUSG00000026610	Esrrg	0.8121	8.40763E-05	0.00518468
ENSMUSG00000120732	ENSMUSG00000120732	0.39854	8.54975E-05	0.005248761
ENSMUSG00000027168	Pax6	1.30604	8.72167E-05	0.00533387

ENSMUSG00000063626	Unc5d	-0.7371	8.87704E-05	0.005408245
ENSMUSG00000031170	Slc38a5	1.07008	9.24715E-05	0.005612392
ENSMUSG00000045404	Kcnk13	0.66051	9.3118E-05	0.005630303
ENSMUSG00000047842	Diras2	-0.56692	9.37039E-05	0.005644428
ENSMUSG00000033249	Hsf4	-0.52648	9.46439E-05	0.0056797
ENSMUSG00000079304	Tex52	0.95506	9.64925E-05	0.005769029
ENSMUSG00000053477	Tcf4	0.5551	9.80443E-05	0.005840014
ENSMUSG00000023267	Gabrr2	-1.00253	0.0001003	0.005930267
ENSMUSG00000031176	Dynlt3	0.55504	0.000100219	0.005930267
ENSMUSG00000040938	Slc16a11	-0.56825	0.000101337	0.005969581
ENSMUSG00000040759	Cmtm5	0.48361	0.000102526	0.00599554
ENSMUSG00000110633	Gm32122	1.92887	0.000102514	0.00599554
ENSMUSG00000049538	Adamts16	-0.63645	0.000103005	0.006001619
ENSMUSG00000112112	Gm48508	1.36172	0.0001038	0.006026066
ENSMUSG00000017057	Il13ra1	0.94711	0.000105231	0.006087087
ENSMUSG00000034336	Ina	0.37973	0.000106221	0.006122207
ENSMUSG00000015202	Cnksr3	0.67904	0.000111831	0.006422464
ENSMUSG00000027562	Car2	0.42137	0.000114418	0.006455351
ENSMUSG00000028412	Slc44a1	0.4152	0.000114177	0.006455351
ENSMUSG00000035095	Fam167a	2.54779	0.000113659	0.006455351
ENSMUSG00000091475	Cerox1	0.55118	0.000113689	0.006455351
ENSMUSG00000095041	ENSMUSG00000095041	-1.48559	0.000113446	0.006455351
ENSMUSG00000029659	Medag	0.95714	0.000120805	0.006768024
ENSMUSG00000041669	Prima1	0.81951	0.00012054	0.006768024
ENSMUSG00000052609	Plekhg3	0.50462	0.0001214	0.006777685
ENSMUSG00000031385	Plxnb3	0.56959	0.000122355	0.00680725
ENSMUSG00000032766	Gng11	0.76634	0.000124048	0.006830779
ENSMUSG00000034570	Inpp5j	0.49746	0.00012407	0.006830779
ENSMUSG00000039936	Pik3cd	0.46097	0.00012426	0.006830779
ENSMUSG00000049721	Gal3st1	0.53075	0.000124483	0.006830779
ENSMUSG00000017734	Dbndd2	0.52661	0.000126276	0.006882066
ENSMUSG00000028249	Sdcbp	0.51775	0.00012599	0.006882066
ENSMUSG00000023913	Pla2g7	0.45515	0.000126816	0.006888041
ENSMUSG00000054871	Tmem158	-0.46759	0.00012749	0.006901253
ENSMUSG00000058669	Nkx2-9	1.65236	0.000128108	0.006911356
ENSMUSG00000021303	Gng4	-0.55619	0.000130965	0.007034977
ENSMUSG00000049493	Pls1	0.51456	0.000131277	0.007034977
ENSMUSG00000008575	Nfib	0.54962	0.000132019	0.007051112
ENSMUSG00000025867	Cplx2	-0.36716	0.000133224	0.007091867
ENSMUSG00000044317	Gpr4	0.99734	0.00013723	0.007280931
ENSMUSG00000026207	Speg	-0.34341	0.000142536	0.007522279
ENSMUSG00000027506	Tpd52	0.37838	0.000142718	0.007522279
ENSMUSG00000005611	Irag1	1.37937	0.000147287	0.007737642
ENSMUSG00000021567	Nkd2	-0.92256	0.000150654	0.007862946

ENSMUSG00000094626	Tmem121b	-0.77515	0.000150342	0.007862946
ENSMUSG00000004894	Hapln2	0.66399	0.000152018	0.007908414
ENSMUSG00000003934	Efnb3	0.53499	0.000152776	0.007922078
ENSMUSG00000018965	Ywhah	0.27772	0.000154361	0.007952816
ENSMUSG00000037062	Sh3glb1	0.3518	0.000154089	0.007952816
ENSMUSG00000035187	Nkx6-1	2.20811	0.000155684	0.007995283
ENSMUSG00000032349	Elovl5	0.4483	0.000156888	0.008031348
ENSMUSG00000031297	Slc7a3	-0.42615	0.000159486	0.008112513
ENSMUSG00000033287	Kctd17	0.31801	0.000159089	0.008112513
ENSMUSG00000052852	Reep1	0.32721	0.000163266	0.008278492
ENSMUSG00000091264	Smim13	0.43703	0.000165537	0.008367202
ENSMUSG00000028280	Gabrr1	-1.82766	0.000167324	0.00840448
ENSMUSG00000037679	Inf2	0.48743	0.000167152	0.00840448
ENSMUSG00000022180	Slc7a8	0.54883	0.000169577	0.008489262
ENSMUSG00000024897	Apba1	0.42628	0.000170923	0.008489262
ENSMUSG00000035296	Sgcg	3.39722	0.000171131	0.008489262
ENSMUSG00000098682	Otx2os1	-2.19278	0.000170139	0.008489262
ENSMUSG00000000037	Scml2	-1.56037	0.000175249	0.008647792
ENSMUSG00000025503	Taldo1	0.48873	0.000175406	0.008647792
ENSMUSG00000046159	Chrm3	-0.49832	0.000176854	0.00867111
ENSMUSG00000052681	Rap1b	0.40567	0.000176961	0.00867111
ENSMUSG00000025272	Tro	-0.30995	0.000180321	0.008747849
ENSMUSG00000033740	St18	0.73258	0.000180331	0.008747849
ENSMUSG00000055692	Tmem191	-0.5017	0.000180711	0.008747849
ENSMUSG00000056445	Hoxaas2	4.0003	0.000180411	0.008747849
ENSMUSG00000036402	Gng12	0.39021	0.000183175	0.008840393
ENSMUSG00000000305	Cdh4	-0.77802	0.000185647	0.008879458
ENSMUSG00000011256	Adam19	0.52123	0.000185425	0.008879458
ENSMUSG00000029223	Uchl1	0.5551	0.000185253	0.008879458
ENSMUSG00000036585	Fgf1	0.5462	0.000187471	0.008913479
ENSMUSG00000040640	Erc2	0.48386	0.000187046	0.008913479
ENSMUSG00000006403	Adamts4	0.59807	0.000191358	0.009071384
ENSMUSG00000005672	Kit	-0.612	0.000194016	0.009116461
ENSMUSG00000031492	Chrnb3	0.9336	0.000193661	0.009116461
ENSMUSG00000074794	Arrdc3	0.70938	0.000193988	0.009116461
ENSMUSG00000024659	Anxa1	1.52997	0.00019526	0.009148085
ENSMUSG00000004936	Map2k1	-0.28876	0.000202893	0.009443131
ENSMUSG00000034327	Kctd9	0.62232	0.000203325	0.009443131
ENSMUSG00000052516	Robo2	-0.62941	0.000202931	0.009443131
ENSMUSG00000041193	Pla2g5	-1.9439	0.000205834	0.009532026
ENSMUSG00000022514	Il1rap	0.5607	0.000206819	0.009550047
ENSMUSG00000002900	Lamb1	-0.71557	0.000208181	0.009585285
ENSMUSG00000026668	Ucma	2.52003	0.000209338	0.009610965
ENSMUSG00000027009	Itga4	1.07497	0.000213936	0.009784791

ENSMUSG00000032502	Stac	-1.03189	0.000214346	0.009784791
ENSMUSG00000044734	Serpinb1a	0.83559	0.000215842	0.009825111
ENSMUSG00000029651	Mtus2	-0.44989	0.000219242	0.009951619
ENSMUSG00000032936	Camkv	-0.71674	0.000223836	0.010112053
ENSMUSG00000056966	Gjc3	0.4862	0.000224039	0.010112053
ENSMUSG00000004035	Gstm7	0.60254	0.000226436	0.010191545
ENSMUSG00000029348	Asphd2	-0.36129	0.000227915	0.010229376
ENSMUSG00000027200	Sema6d	0.52394	0.000232474	0.010404831
ENSMUSG00000020458	Rtn4	0.45268	0.000234386	0.010444726
ENSMUSG00000059182	Skap2	0.51466	0.000234669	0.010444726
ENSMUSG00000054934	Kcnmb4	1.23732	0.000236666	0.01050088
ENSMUSG00000078771	Evi2a	0.50326	0.000237241	0.01050088
ENSMUSG00000053519	Kcnip1	-0.56661	0.000240033	0.010595177
ENSMUSG00000028717	Tal1	-0.69423	0.000243082	0.010700265
ENSMUSG00000081225	Cyp2j12	0.90218	0.000245968	0.010797645
ENSMUSG00000025020	Slit1	-0.4908	0.000252347	0.011047413
ENSMUSG00000068876	Cgn	-0.64613	0.000255043	0.011135013
ENSMUSG00000031298	Adgrg2	-0.86495	0.000256546	0.011170206
ENSMUSG00000090121	Abhd12b	4.0331	0.000262468	0.011397104
ENSMUSG00000020396	Nefh	0.64796	0.000268454	0.011625527
ENSMUSG00000026872	Zeb2	0.40412	0.000271048	0.011688467
ENSMUSG00000047773	Ankfn1	-1.08085	0.000271367	0.011688467
ENSMUSG00000022197	Pdzd2	-0.73593	0.000273836	0.011700468
ENSMUSG00000050248	Evc2	-0.6549	0.000273498	0.011700468
ENSMUSG00000085828	Gm15612	-0.85148	0.000272459	0.011700468
ENSMUSG00000020261	Slc36a1	0.33222	0.000274772	0.011709225
ENSMUSG00000020090	Npffr1	-1.19075	0.000276015	0.011731003
ENSMUSG00000078532	Nkain1	0.53093	0.000278038	0.011785724
ENSMUSG00000060716	Plekhh1	0.50775	0.000281284	0.011879723
ENSMUSG00000064043	Trerf1	-0.54667	0.000281738	0.011879723
ENSMUSG00000014226	Cacybp	0.34988	0.000283854	0.011937532
ENSMUSG00000003657	Calb2	0.54467	0.000290044	0.012165897
ENSMUSG00000022270	Retreg1	0.37547	0.00029171	0.012183047
ENSMUSG00000038695	Josd2	0.3845	0.000292734	0.012183047
ENSMUSG00000091712	Sec14l5	0.64178	0.000292637	0.012183047
ENSMUSG00000022500	Litaf	0.53156	0.000300257	0.012463787
ENSMUSG00000072235	Tuba1a	0.41298	0.000307104	0.012715041
ENSMUSG00000018340	Anxa6	0.45437	0.000314456	0.012952512
ENSMUSG00000028804	Csmd2	-0.43275	0.000314298	0.012952512
ENSMUSG00000049191	Rtl5	-0.51183	0.000316592	0.013007046
ENSMUSG00000004231	Pax2	2.13802	0.000327835	0.013264904
ENSMUSG00000027712	Anxa5	0.57152	0.000327245	0.013264904
ENSMUSG00000028955	Vamp3	0.38594	0.00032731	0.013264904
ENSMUSG00000032532	Cck	0.56343	0.000327304	0.013264904

ENSMUSG00000036564	Ndrg4	0.30801	0.000324513	0.013264904
ENSMUSG00000051335	Gfod1	0.55157	0.000327111	0.013264904
ENSMUSG00000026888	Grb14	0.36999	0.000329174	0.013285548
ENSMUSG00000025981	Coq10b	0.77578	0.000337616	0.013592032
ENSMUSG00000039910	Cited2	0.40959	0.000340929	0.013690983
ENSMUSG00000068798	Rap1a	0.50621	0.000342661	0.01372613
ENSMUSG00000020151	Ptprr	-0.55796	0.000346223	0.013829259
ENSMUSG00000055717	Slain1	0.41466	0.000346961	0.013829259
ENSMUSG00000025959	Klf7	0.41902	0.000350728	0.013944712
ENSMUSG00000023473	Celsr3	-0.34969	0.000352404	0.013976671
ENSMUSG00000020604	Arsg	0.44251	0.000354773	0.014008747
ENSMUSG00000037653	Kctd8	-0.57314	0.000354962	0.014008747
ENSMUSG00000062309	Rpp25	-0.47765	0.000357847	0.014087903
ENSMUSG00000020331	Hcn2	0.36043	0.000364374	0.014309705
ENSMUSG00000045648	Vwc2l	0.60242	0.000366555	0.014360161
ENSMUSG00000025348	Itga7	-0.64287	0.000368941	0.014418411
ENSMUSG00000046500	Tafa4	1.87624	0.000384135	0.014975648
ENSMUSG00000000126	Wnt9a	1.51797	0.000390936	0.015203809
ENSMUSG00000023031	Cela1	1.25702	0.00039427	0.015222639
ENSMUSG00000042686	Jph1	0.80985	0.000394084	0.015222639
ENSMUSG00000100837	1700063D05Rik	0.95085	0.000394208	0.015222639
ENSMUSG00000036422	Pcdh8	-0.56783	0.000399922	0.015403719
ENSMUSG00000045962	Wnk1	0.47225	0.000401455	0.015425693
ENSMUSG00000058099	Nfam1	-0.91678	0.000408465	0.015657488
ENSMUSG00000021721	Htr1a	-0.44609	0.000409603	0.015663665
ENSMUSG00000006522	Itih3	-0.28049	0.000414383	0.015808721
ENSMUSG00000003575	Crtc1	-0.3581	0.0004191	0.0159494
ENSMUSG00000045007	Tubg2	-0.34352	0.000420062	0.0159494
ENSMUSG00000038400	Pmepa1	0.47425	0.000421216	0.01595544
ENSMUSG00000068735	Trp53i11	-0.5725	0.000422997	0.015985106
ENSMUSG00000061048	Cdh3	-2.55821	0.000424633	0.016009165
ENSMUSG00000019055	Plod1	0.48495	0.000427802	0.016090771
ENSMUSG00000015806	Qdpr	0.37798	0.000433759	0.016200757
ENSMUSG00000023972	Ptk7	-0.69115	0.000432712	0.016200757
ENSMUSG00000043843	Tmem145	-0.37752	0.00043325	0.016200757
ENSMUSG00000042429	Adora1	0.32144	0.000437379	0.016297968
ENSMUSG00000096257	Ccer2	-0.9778	0.00044319	0.016476195
ENSMUSG00000100706	Gm19744	-1.40558	0.00044564	0.016528896
ENSMUSG00000032679	Cd59a	0.75909	0.000447444	0.01655749
ENSMUSG00000021972	Hmbox1	0.41404	0.000464319	0.017142357
ENSMUSG00000092274	Neat1	0.49948	0.000468387	0.01725279
ENSMUSG00000027335	Adra1d	1.40259	0.000478391	0.017580867
ENSMUSG00000118667	Ahnak2	0.93176	0.000483501	0.017727996
ENSMUSG00000020570	Sypl	0.38033	0.000492652	0.01789386

ENSMUSG00000020841	Cpd	0.45046	0.000493144	0.01789386
ENSMUSG00000024064	Galnt14	-0.79309	0.000493024	0.01789386
ENSMUSG00000024816	Frmd8	0.53155	0.000491839	0.01789386
ENSMUSG00000049571	Cfap46	-0.49262	0.000493608	0.01789386
ENSMUSG00000026730	Pter	0.96771	0.000502944	0.018191148
ENSMUSG00000035451	Foxa1	4.24168	0.000510412	0.018404733
ENSMUSG00000075289	Carns1	0.66078	0.000511147	0.018404733
ENSMUSG00000023827	Agpat4	0.58896	0.000512931	0.018427551
ENSMUSG00000021508	Cxcl14	-0.45706	0.000518959	0.018602433
ENSMUSG00000002012	Pnck	-0.43548	0.000523253	0.018714472
ENSMUSG00000021278	Amn	-0.78077	0.000527581	0.018827236
ENSMUSG00000037624	Kcnk2	-0.55764	0.000533279	0.018988295
ENSMUSG00000109847	Gm45278	-1.98187	0.00053462	0.018993829
ENSMUSG00000109233	Gm44866	0.70239	0.000560588	0.019872361
ENSMUSG00000022687	Boc	-0.48664	0.000570024	0.019942141
ENSMUSG00000031137	Fgf13	0.28536	0.000565199	0.019942141
ENSMUSG00000041889	Shisa4	0.32363	0.000569364	0.019942141
ENSMUSG00000043673	Kcns3	0.78732	0.000567167	0.019942141
ENSMUSG00000043811	Rtn4r	0.53051	0.000564644	0.019942141
ENSMUSG00000044783	Hjurp	-0.32745	0.00056888	0.019942141
ENSMUSG00000043456	Zfp536	0.53681	0.000573565	0.020022308
ENSMUSG00000026991	Pkp4	0.42447	0.000577818	0.020126901
ENSMUSG00000022742	Cpox	0.43054	0.000580746	0.020166685
ENSMUSG00000056972	Magel2	-0.50484	0.000581477	0.020166685
ENSMUSG00000020950	Foxg1	5.76142	0.000590013	0.020406196
ENSMUSG00000022678	Nde1	0.58949	0.00059093	0.020406196
ENSMUSG00000056121	Fez2	0.43374	0.000595894	0.020489278
ENSMUSG00000095407	Tmem200c	-0.51222	0.000595284	0.020489278
ENSMUSG00000021036	Sptlc2	0.33032	0.000601179	0.020626742
ENSMUSG00000027347	Rasgrp1	1.35595	0.000606853	0.020776929
ENSMUSG00000059456	Ptk2b	1.53549	0.000609047	0.020807574
ENSMUSG00000001833	Septin7	0.4477	0.000611923	0.020861362
ENSMUSG00000030340	Scnn1a	1.08169	0.000616486	0.020972294
ENSMUSG00000015850	Adamtsl4	0.55475	0.000618735	0.021004219
ENSMUSG00000031860	Pbx4	-0.53386	0.00062258	0.021090048
ENSMUSG00000004933	Matk	-0.2994	0.000624478	0.021109716
ENSMUSG00000008658	Rbfox1	-0.62496	0.000629811	0.021245175
ENSMUSG00000055471	Alk	-0.63379	0.000632827	0.021302062
ENSMUSG00000061462	Obscn	1.75839	0.000645802	0.021693253
ENSMUSG00000015619	Gata3	-0.62776	0.000665786	0.022274365
ENSMUSG00000037280	Galnt6	0.56632	0.000668553	0.022274365
ENSMUSG00000037463	Fbxo27	-0.47862	0.000669727	0.022274365
ENSMUSG00000042810	Krba1	-0.34566	0.000670052	0.022274365
ENSMUSG00000086228	Ubap1l	-0.43181	0.000667043	0.022274365

ENSMUSG00000024063	Lbh	0.37908	0.000674949	0.022390696
ENSMUSG00000025283	Sat1	0.35314	0.000676809	0.022405998
ENSMUSG00000031932	Gpr83	-0.65317	0.000679282	0.022441532
ENSMUSG00000024942	Capn1	0.4003	0.000685936	0.022614731
ENSMUSG00000031066	Usp11	-0.32261	0.000691935	0.022765656
ENSMUSG00000043659	Npsr1	-1.34995	0.000698107	0.022921657
ENSMUSG00000014786	Slc9a5	-0.33525	0.000700209	0.022943649
ENSMUSG00000026482	Rgl1	-0.47563	0.000703239	0.022995905
ENSMUSG00000034780	B3galt1	0.4872	0.000705097	0.023009702
ENSMUSG00000026765	Lypd6b	-0.76785	0.000708486	0.023073323
ENSMUSG00000023809	Rps6ka2	-0.31586	0.000720524	0.023417767
ENSMUSG00000015341	Golga7	0.32041	0.000724463	0.02345066
ENSMUSG00000042564	Fam227a	-0.54503	0.000723913	0.02345066
ENSMUSG00000030987	Stim1	-0.31714	0.000727482	0.02349764
ENSMUSG00000033039	Micall1	0.39702	0.000728848	0.02349764
ENSMUSG00000112168	Gm34776	0.80934	0.000731773	0.023544585
ENSMUSG00000001552	Jup	0.46313	0.000744301	0.023804264
ENSMUSG00000018217	Pmp22	0.48911	0.000743606	0.023804264
ENSMUSG00000020848	Doc2b	-0.4742	0.000741745	0.023804264
ENSMUSG00000005357	Slc1a6	-0.61351	0.000753615	0.023958678
ENSMUSG00000026675	Hsd17b7	0.70401	0.000752772	0.023958678
ENSMUSG00000036580	Spg20	0.38872	0.000753344	0.023958678
ENSMUSG00000047181	Samd14	-0.35237	0.000757395	0.024031159
ENSMUSG00000032002	Dcun1d5	0.4564	0.000772376	0.024458062
ENSMUSG00000032788	Pdxk	-0.36806	0.000776308	0.024534092
ENSMUSG00000006235	Epor	-0.77826	0.000778277	0.024547906
ENSMUSG00000030788	Rnf141	0.40596	0.000783094	0.024602977
ENSMUSG00000053626	Tll1	1.15939	0.000781929	0.024602977
ENSMUSG00000045193	Cirbp	-0.428	0.000785411	0.024627476
ENSMUSG00000053166	Cdh22	-0.40239	0.000788286	0.024669339
ENSMUSG00000066510	Ankdd1a	-1.13138	0.000791345	0.024679693
ENSMUSG00000073982	Rhog	0.48486	0.000791697	0.024679693
ENSMUSG00000034317	Trim59	0.97375	0.000797719	0.024819132
ENSMUSG00000046324	Ermp1	0.34111	0.000801617	0.024892074
ENSMUSG00000022419	Deptor	0.59615	0.000811289	0.025048444
ENSMUSG00000026576	Atp1b1	0.30423	0.000808563	0.025048444
ENSMUSG00000041534	Rbp3	-2.38192	0.000811343	0.025048444
ENSMUSG00000024517	Grp	1.17947	0.000824354	0.025401189
ENSMUSG00000001891	Ugp2	0.51636	0.000845194	0.025827115
ENSMUSG00000020524	Gria1	-0.48288	0.000845047	0.025827115
ENSMUSG00000035456	Prdm8	1.81511	0.00084411	0.025827115
ENSMUSG00000054196	Cthrc1	0.67447	0.000841072	0.025827115
ENSMUSG00000110080	Gm6145	0.34142	0.000846236	0.025827115
ENSMUSG00000043398	Gpr135	-0.52236	0.000850132	0.025896707

ENSMUSG00000002007	Srpk3	0.52499	0.000863829	0.026263993
ENSMUSG00000032589	Bsn	0.24756	0.000876882	0.026610364
ENSMUSG00000037846	Rtkn2	0.78162	0.000893631	0.027047629
ENSMUSG00000059434	Gckr	1.95165	0.000894667	0.027047629
ENSMUSG00000020032	Nuak1	0.51788	0.000900943	0.027134978
ENSMUSG00000032549	Rab6b	0.2763	0.000899318	0.027134978
ENSMUSG00000027204	Fbn1	-0.57349	0.000907844	0.027291537
ENSMUSG00000040424	Hipk4	-0.527	0.00092145	0.027648669
ENSMUSG00000023961	Enpp4	0.51136	0.000925714	0.027708429
ENSMUSG00000032356	Rasgrf1	-0.31344	0.0009269	0.027708429
ENSMUSG00000020486	Septin4	0.27865	0.000931016	0.02777964
ENSMUSG00000019790	Stxbp5	-0.33628	0.000944835	0.02813958
ENSMUSG00000056306	Sertm1	0.50858	0.000952508	0.028315456
ENSMUSG00000031659	Adcy7	-0.49961	0.0009588	0.028449738
ENSMUSG00000041828	Abca8a	0.59432	0.000962445	0.028452491
ENSMUSG00000085830	Grin1os	-0.5942	0.000962024	0.028452491
ENSMUSG00000021948	Prkcd	2.65463	0.000965135	0.028479492
ENSMUSG00000079055	Slc8a3	-0.30047	0.000969701	0.02856162
ENSMUSG00000021536	Adcy2	-0.5016	0.000977417	0.028683419
ENSMUSG00000039865	Slc44a3	3.52087	0.000976296	0.028683419
ENSMUSG00000053475	Tnfaip6	0.48619	0.000986242	0.028889507
ENSMUSG00000029868	Trpv6	-0.68117	0.000988243	0.028895289
ENSMUSG00000027297	Ltk	-0.51427	0.000993636	0.029000041
ENSMUSG00000025407	Gli1	-0.58553	0.001002449	0.029133272
ENSMUSG00000038156	Spon1	0.35466	0.001003415	0.029133272
ENSMUSG00000039801	Cplane1	-0.28339	0.001003655	0.029133272
ENSMUSG00000017740	Slc12a5	-0.40972	0.00101393	0.029325281
ENSMUSG00000034295	Fhod3	-0.43861	0.001012649	0.029325281
ENSMUSG00000033491	Prss35	1.1638	0.001041915	0.030080371
ENSMUSG00000036006	Ripor2	-0.54342	0.001051659	0.030252658
ENSMUSG00000101356	Gm28876	1.88424	0.001049801	0.030252658
ENSMUSG00000004626	Stxbp2	0.61361	0.001059517	0.030424081
ENSMUSG00000023015	Racgap1	-0.50296	0.001066387	0.030512007
ENSMUSG00000068267	Cenpb	0.31665	0.001064838	0.030512007
ENSMUSG00000040268	Plekha1	0.33237	0.001070658	0.030522474
ENSMUSG00000041879	Ipo9	-0.33012	0.001073752	0.030522474
ENSMUSG00000051067	Lingo3	-0.49365	0.001074373	0.030522474
ENSMUSG00000058420	Syt17	1.5064	0.001072024	0.030522474
ENSMUSG00000033174	Mgll	-0.53062	0.001081314	0.030665309
ENSMUSG00000027339	Rassf2	0.35469	0.001084499	0.030701278
ENSMUSG00000024516	Sec11c	0.41706	0.001094036	0.030916651
ENSMUSG00000025813	Homer2	0.5114	0.001097727	0.030966334
ENSMUSG00000059361	Nrsn2	-0.33999	0.001104485	0.031102229
ENSMUSG00000075254	Heg1	0.41301	0.001109971	0.031201879

ENSMUSG00000030849	Fgfr2	0.37069	0.001115804	0.031291688
ENSMUSG00000056158	Car10	-0.47506	0.001117072	0.031291688
ENSMUSG00000022055	Nefl	0.46382	0.001120492	0.031332708
ENSMUSG00000024899	Papss2	0.50741	0.00112954	0.03149425
ENSMUSG00000045994	B3gat1	-0.28409	0.0011302	0.03149425
ENSMUSG00000025318	Jph3	-0.3144	0.001133902	0.031542566
ENSMUSG00000044681	Cnpy1	0.93337	0.001143579	0.031756604
ENSMUSG00000048899	Rimkla	0.44604	0.001146629	0.031786212
ENSMUSG00000008475	Arpc5	0.37944	0.001153836	0.031877394
ENSMUSG00000028876	Epha10	-0.3161	0.001153897	0.031877394
ENSMUSG00000050511	Oprd1	1.15574	0.001156705	0.031899983
ENSMUSG00000078307	AI593442	0.57044	0.001177505	0.0324178
ENSMUSG00000032733	Snx33	0.41897	0.001182038	0.032481596
ENSMUSG00000048616	Nog	-0.82546	0.001183876	0.032481596
ENSMUSG00000027236	Eif3j1	0.59252	0.001199886	0.032864573
ENSMUSG00000038677	Scube3	-0.66328	0.001204369	0.032931062
ENSMUSG00000047759	Hs3st3a1	-0.84262	0.001226645	0.033483021
ENSMUSG00000028546	Elavl4	0.43059	0.00123477	0.033508459
ENSMUSG00000035722	Abca7	0.46797	0.001235942	0.033508459
ENSMUSG00000041923	Nol4	-0.42117	0.001233894	0.033508459
ENSMUSG00000049303	Syt12	-0.44424	0.00123014	0.033508459
ENSMUSG00000046613	Vwa5b2	-0.32573	0.001241781	0.033609891
ENSMUSG00000035770	Dync1li2	0.25364	0.001268733	0.034281461
ENSMUSG00000022199	Slc22a17	-0.23429	0.001281942	0.034580053
ENSMUSG00000010476	Ebf3	-0.66754	0.001287899	0.034682357
ENSMUSG00000027840	Wnt2b	-0.73946	0.001292918	0.034707004
ENSMUSG00000095562	Gm55594	-7.25458	0.001293146	0.034707004
ENSMUSG00000028064	Sema4a	-0.35761	0.001303251	0.034919723
ENSMUSG00000002578	Ikzf4	-0.54764	0.001312975	0.035073398
ENSMUSG00000022816	Fstl1	0.57688	0.001313364	0.035073398
ENSMUSG00000094441	Zfp955a	0.72627	0.001321936	0.035243575
ENSMUSG00000031990	Jam3	0.48558	0.001325761	0.035286812
ENSMUSG00000029219	Slc10a4	3.18044	0.001356096	0.035915255
ENSMUSG00000029314	Gpat3	0.61363	0.001355389	0.035915255
ENSMUSG00000033985	Tesk2	0.53851	0.001354599	0.035915255
ENSMUSG00000015377	Dennd6b	-0.31953	0.001364409	0.03607578
ENSMUSG00000024940	Ltbp3	-0.32206	0.00136764	0.036101641
ENSMUSG00000021198	Unc79	-0.37136	0.001380418	0.036378997
ENSMUSG00000030770	Parva	-0.26571	0.001388541	0.036492169
ENSMUSG00000042662	Dusp15	0.48408	0.001389267	0.036492169
ENSMUSG00000030270	Cpne9	0.54512	0.001395539	0.036596933
ENSMUSG00000018909	Arrb1	-0.33319	0.001409925	0.036853552
ENSMUSG00000036198	Arhgap36	0.64807	0.001408137	0.036853552
ENSMUSG00000003469	Phyhip	-0.2836	0.001422245	0.036953102

ENSMUSG00000027860	Vangl1	0.60016	0.001422958	0.036953102
ENSMUSG00000047712	Ust	0.48734	0.001422204	0.036953102
ENSMUSG00000070372	Capza1	0.33469	0.001422854	0.036953102
ENSMUSG00000050211	Pla2g4e	-0.6891	0.0014296	0.037065515
ENSMUSG00000025962	Fastkd2	0.43854	0.001432305	0.037075639
ENSMUSG00000000942	Hoxa4	8.00797	0.001450468	0.03748524
ENSMUSG00000007030	Vwa7	2.11112	0.001474566	0.037924512
ENSMUSG00000037824	Tspan14	0.49754	0.001472801	0.037924512
ENSMUSG00000053310	Nrgn	-0.6579	0.001472068	0.037924512
ENSMUSG00000020287	Mpg	-0.54074	0.00149841	0.038475997
ENSMUSG00000049336	Tenm2	-0.41331	0.001515132	0.038843148
ENSMUSG00000068263	Efcc1	-0.54473	0.001518544	0.038868407
ENSMUSG00000025571	Tnrc6c	-0.3181	0.001543472	0.039443471
ENSMUSG00000036185	Sapcd1	1.5926	0.001557366	0.039735144
ENSMUSG00000079658	Eloc	0.49106	0.00156536	0.039812334
ENSMUSG00000091586	Cyp4f17	-0.51762	0.001564228	0.039812334
ENSMUSG00000022864	D16Ertd472e	0.66649	0.00158238	0.040114547
ENSMUSG00000055067	Smyd3	-0.32418	0.001584754	0.040114547
ENSMUSG00000117864	Gm19500	0.65351	0.001583041	0.040114547
ENSMUSG00000015672	Mrpl32	0.6228	0.001592577	0.040248992
ENSMUSG00000026959	Grin1	-0.39568	0.001623986	0.040978142
ENSMUSG00000045733	Sprn	0.32504	0.001632697	0.041133182
ENSMUSG00000044674	Fzd1	-0.65947	0.001646589	0.041418048
ENSMUSG00000030276	Ttll3	-0.41435	0.001653469	0.041525917
ENSMUSG00000036503	Rnf13	0.33201	0.001662213	0.041644505
ENSMUSG00000066189	Cacng3	-0.41074	0.001663389	0.041644505
ENSMUSG00000038034	Igsf8	-0.30782	0.001667736	0.041688184
ENSMUSG00000079508	Apoo	0.78565	0.001678167	0.041883601
ENSMUSG00000002489	Tiam1	0.48465	0.00168291	0.041936648
ENSMUSG00000021998	Lcp1	0.48817	0.001728274	0.04300022
ENSMUSG00000062044	Lmtk3	-0.31984	0.001734085	0.043077897
ENSMUSG00000009739	Pou6f1	-0.34384	0.001746411	0.043316939
ENSMUSG00000050621	Rps27rt	0.97102	0.001761715	0.043561677
ENSMUSG00000057897	Camk2b	-0.34784	0.001759791	0.043561677
ENSMUSG00000038495	Otud7b	0.38869	0.001765414	0.04358587
ENSMUSG00000025572	Tmc6	0.59037	0.001772552	0.043694783
ENSMUSG00000015143	Actn1	-0.30236	0.001777032	0.043717224
ENSMUSG00000031841	Cdh13	-0.43091	0.001781648	0.043717224
ENSMUSG00000049796	Crh	1.23465	0.001779059	0.043717224
ENSMUSG00000029376	Mthfd2l	-0.46761	0.001792646	0.043919835
ENSMUSG00000058665	En1	1.69463	0.001796342	0.043943186
ENSMUSG00000039298	Cdk5rap2	0.30705	0.00181051	0.044222247
ENSMUSG00000038860	Garnl3	-0.28551	0.001820587	0.044400713
ENSMUSG00000001773	Folh1	0.63124	0.001829223	0.044462814

ENSMUSG00000032855	Pkd1	-0.2486	0.001828048	0.044462814
ENSMUSG00000036737	Oxsr1	0.32695	0.001831458	0.044462814
ENSMUSG00000024053	Emilin2	0.73006	0.001843516	0.044687843
ENSMUSG00000097233	Gm17552	1.03561	0.001851815	0.044821186
ENSMUSG00000004849	Ap1s1	0.30148	0.001873413	0.04526976
ENSMUSG00000018634	Crhr1	0.57746	0.001875998	0.04526976
ENSMUSG00000038301	Snx10	0.37854	0.00188973	0.045532543
ENSMUSG00000081534	Slc48a1	0.2936	0.001906091	0.045857791
ENSMUSG00000042961	Egflam	-0.54381	0.001909435	0.045869389
ENSMUSG00000045532	C1ql1	0.71203	0.001923052	0.046126433
ENSMUSG00000085564	Gm12198	1.81022	0.001925893	0.046126433
ENSMUSG00000004099	Dnmt1	-0.33686	0.001931082	0.046174742
ENSMUSG00000028864	Hgf	-1.26691	0.001933674	0.046174742
ENSMUSG00000021798	Ldb3	0.39413	0.001946354	0.04633943
ENSMUSG00000031026	Trim66	-0.42119	0.001945522	0.04633943
ENSMUSG00000026058	Khdrbs2	-0.60228	0.001955614	0.046429614
ENSMUSG00000028082	Sh3d19	0.58564	0.001955938	0.046429614
ENSMUSG00000038534	Osbpl7	0.36487	0.001969562	0.046683857
ENSMUSG00000022723	Crybg3	0.61859	0.001976879	0.046719079
ENSMUSG00000035407	Kank4	0.51533	0.001975118	0.046719079
ENSMUSG00000087075	Lbhd2	-1.16386	0.001984731	0.04683557
ENSMUSG00000025372	Baiap2	-0.39162	0.00198796	0.046842769
ENSMUSG00000023232	Serinc2	1.36058	0.00199395	0.046914928
ENSMUSG00000026811	St6galnac6	-0.26831	0.002005312	0.047113068
ENSMUSG00000073434	Wdr90	-0.46584	0.002009837	0.047150255
ENSMUSG00000027428	Rbbp9	0.368	0.002018219	0.047277677
ENSMUSG00000034382	AI661453	1.67003	0.002021761	0.04728838
ENSMUSG00000053141	Ptprt	-0.59261	0.002024579	0.04728838
ENSMUSG00000048782	Insc	0.71973	0.002038957	0.047554879
ENSMUSG00000030342	Cd9	0.37377	0.002042605	0.047570724
ENSMUSG00000069171	Nr2f1	0.345	0.002052929	0.047741778
ENSMUSG00000034818	Celf5	-0.36156	0.00205787	0.047787326
ENSMUSG00000032178	Ilf3	-0.23523	0.002067408	0.047870056
ENSMUSG00000097767	Miat	-0.38932	0.002066179	0.047870056
ENSMUSG00000033009	Ogfod1	-0.40281	0.002077408	0.047898406
ENSMUSG00000075415	Fnbp1	0.3157	0.002072058	0.047898406
ENSMUSG00000109901	Chmp1b	0.4751	0.0020776	0.047898406
ENSMUSG00000026737	Pip4k2a	0.35734	0.002094438	0.04821721
ENSMUSG00000015396	Cd83	-0.52682	0.002102058	0.048323209
ENSMUSG00000031224	Magee2	-0.55109	0.002106276	0.048350799
ENSMUSG00000026447	Pik3c2b	0.3657	0.002125334	0.048718489
ENSMUSG00000022337	Emc2	0.43007	0.002137985	0.048938481
ENSMUSG00000033590	Myo5c	1.067	0.002149843	0.049139698
ENSMUSG00000021708	Rasgrf2	-0.42006	0.002153533	0.049153935

ENSMUSG00000039037	St6galnac5	0.61603	0.002163318	0.04923699
ENSMUSG00000070498	Tmem132b	0.35617	0.002162799	0.04923699
ENSMUSG00000020150	Gamt	0.48661	0.002166802	0.049246348
ENSMUSG00000037613	Tnfrsf23	1.22173	0.002183309	0.049551213
ENSMUSG00000029999	Tgfa	0.41314	0.002188251	0.04959313

Supplementary Table S5: Transcriptomic profile of LYC intervention in a PD model: Upregulation of key transporter genes implicated in neuroprotective potential.

PD-1_fpkm, PD-2_fpkm, and PD-3_fpkm are the FPKM values of the gene in three independent biological replicates of the PD group. LYC-PD-1_fpkm, LYC-PD-2_fpkm, and LYC-PD-3_fpkm are the FPKM values of the gene in three independent biological replicates of the lycopene intervention (LYC-PD) group. p-value represents the significance p-value for the differential expression of the gene. padj is the adjusted p-value following multiple testing correction. The results indicate that the significant upregulation of Slc6a3 and Slc17a7 may be closely associated with dopaminergic neuron function and neuroprotective mechanisms.

**gene_name**	**PD-3_fpkm**	**PD-1_fpkm**	**PD-2_fpkm**	**LYC-PD-1_fpkm**	**LYC-PD-2_fpkm**	**LYC-PD-3_fpkm**	**pvalue**	**padj**
Slco2a1	0.1156	0.1121	0.1208	0.7554	0.9462	0.3321	7.38081E-13	2.36526E-09
Slc17a7	1.9619	0.8208	0.9934	59.4047	49.2551	10.9847	1.11775E-11	1.98998E-08
Slc6a3	0.5632	7.4967	1.2486	167.8354	139.2629	39.5656	4.14605E-06	0.000738134
Slc45a3	0.475	0.7979	0.4594	1.919	1.4084	1.2225	1.02766E-05	0.001349682
Slc12a2	10.2416	12.8155	11.1187	16.56	16.6251	14.3887	1.28652E-05	0.001493742
